# Supplementary material for: PhysiCell: An open source physics-based cell simulator for 3-D multicellular systems
Source: PLoS Comput Biol. 2018 Feb 23;14(2):e1005991. doi: 10.1371/journal.pcbi.1005991 (PMC5841829; doi:10.1371/journal.pcbi.1005991)
Supplement: S1 Text — Extensive supplemental information including: full mathematical model details, supporting literature, and reference parameter values for breast epithelial cells; expanded numerical implementation details; convergence and validation testing results; full parameter values for the main tests; and an expanded feature comparison of PhysiCell and other 3-D multicellular simulation platforms. (PDF) [file pcbi.1005991.s001.pdf]

Supplementary Materials for:

Ghaffarizadeh et al., PhysiCell: an Open Source Physics-Based Cell Simulator for 3-D Multicellular Systems, PLoS Comput. Biol. (2018, accepted)

Ahmadreza Ghaffarizadeh Randy Heiland Samuel H. Friedman Shannon M. Mumenthaler Paul Macklin

January 23, 2018

Abstract

These are the supplementary materials for [19].

Contents

1 Mathematical model details **2**
1.1 Cell volume . . . 2
1.1.1 Reference parameter values . . . 3
1.2 Cell cycle models . . . 4
1.2.1 General representation of a cell cycle model . . . 4
1.2.2 Cell division . . . 4
1.2.3 Ki67 Advanced Model . . . 5
1.2.4 Ki67 Basic Model . . . 6
1.2.5 Live Cells model . . . 7
1.3 Cell death models . . . 7
1.3.1 Apoptosis . . . 7
1.3.2 Necrosis . . . 8
1.4 Cell mechanics and motion . . . 12
1.4.1 Mechanics potential functions . . . 12
1.4.2 Cell-cell mechanics . . . 13
1.4.3 Cell-BM mechanics . . . 13
1.4.4 Motility . . . 13
1.4.5 Cell orientation . . . 14
1.4.6 Reference parameter values . . . 14

2 Numerical algorithm details **15**
2.1 Decoupling Velocity and Position Updates . . . 15
2.2 ODE solutions . . . 15
2.3 Biotransport . . . 15
2.4 Pseudorandom number generator and probabilities . . . 16
2.5 Choice of time step values . . . 16
2.6 Interaction testing data structure (expanded detail) . . . 16
2.7 Computational cost estimates (expanded detail) . . . 16

3 Convergence and validation testing **17**
3.1 Cell volume . . . 17
3.1.1 Test 1: cell volume in the Ki67 advanced model (deterministic variant) . . . 17
3.1.2 Test 2: cell volume in the apoptosis model . . . 18
3.1.3 Test 3: cell volume in the necrosis model . . . 19
3.1.4 Running the code for the volume tests . . . 19
3.2 Cell mechanics and motion . . . 20

3.2.1 Test 1: pairwise cell mechanics . . . 20
3.2.2 Test 2: compressed spheroid example . . . 20
3.2.3 Running the code for the mechanics tests . . . 21
3.3 Cell phenotype changes . . . 21
3.3.1 Analytical solutions . . . 22
3.3.2 Validation testing . . . 23
3.3.3 Running the phenotype testing code . . . 23

4 Parameter values for the main examples **23**
4.1 Main example 1: hanging drop spheroids . . . 23
4.2 Main example 2: ductal carcinoma in situ (DCIS) . . . 23
4.3 Running the code for the main examples . . . 23

5 Comparison of PhysiCell with other major 3-D multicellular simulators **26**
5.1 Notes . . . 26

Bibliography **28**

1 Mathematical model details

In this section, we define the mathematical models currently implemented in PhysiCell. Moreover, we provide reference parameter values corresponding to human cancer cells. We note that both the models and the reference parameter values may be updated over time. Users citing these models or reference parameter values should note the version number of PhysiCell (currently PhysiCell Version 1.2.2).

1.1 Cell volume

Updating prior work [35], each cell agent has a total volume V, which is divided into fluid volume V_{F} and solid biomass volume V_{S}. The solid biomass volume is divided into nuclear solids V_{NS} and cytoplasmic solids V_{CS}. We also track the total nuclear volume ($V_{\mathcal{N}}$) and total cytoplasmic volume ($V_{\mathcal{C}}$). We model these volumes with a simple system of ODEs

$$\frac{dV_{\mathrm{F}}}{dt} = r_{\mathrm{F}}\left(V_{\mathrm{F}}^*(t) - V_{\mathrm{F}}\right) \tag{1}$$

$$\frac{dV_{\mathrm{NS}}}{dt} = r_{\mathrm{N}}\left(V_{\mathrm{NS}}^*(t) - V_{\mathrm{NS}}\right) \tag{2}$$

$$\frac{dV_{\mathrm{CS}}}{dt} = r_{\mathrm{C}}\left(V_{\mathrm{CS}}^*(t) - V_{\mathrm{CS}}\right), \tag{3}$$

where r_{F}, r_{N}, and r_{C} are rate constants, and V_F^*, V_{NS}^* and V_{NC}^* are "target" volumes. The remaining volumes are calculated by:

$$V_{\mathrm{CS}} = \left(1 - \frac{V_{\mathrm{F}}}{V}\right) V_{\mathrm{C}} \tag{4}$$

$$V_{\mathrm{NS}} = \left(1 - \frac{V_{\mathrm{F}}}{V}\right) V_{\mathrm{N}} \tag{5}$$

$$V_{\mathrm{S}} = V_{\mathrm{NS}} + V_{\mathrm{CS}}. \tag{6}$$

We use the constitutive relations

$$V_{\mathrm{CS}}^*(t) = f_{\mathrm{CN}} V_{\mathrm{NS}}^*(t) \tag{7}$$

$$V_{\mathrm{F}}^*(t) = f_{\mathrm{F}} V(t), \tag{8}$$

where, $f_{\mathrm{CN}} = 1/f_{\mathrm{NC}}$ is the target cytoplasmic to nuclear volume ratio. (f_{NC} is the target nuclear:cytoplasmic volume ratio, a more common measure in experimental biology, although most experiments compute this based upon cross-sectional areas rather than 3-D volumes.) f_{F} is the cell's target water fraction. V_{NS}^* will be specified in the cell cycle and death models below. See Sections 1.2 and 1.3. Volume plots during the Ki-67 Advanced cell cycle model are shown in Figure 1, using the reference parameter values in Sections 1.1.1 and 1.2.3.

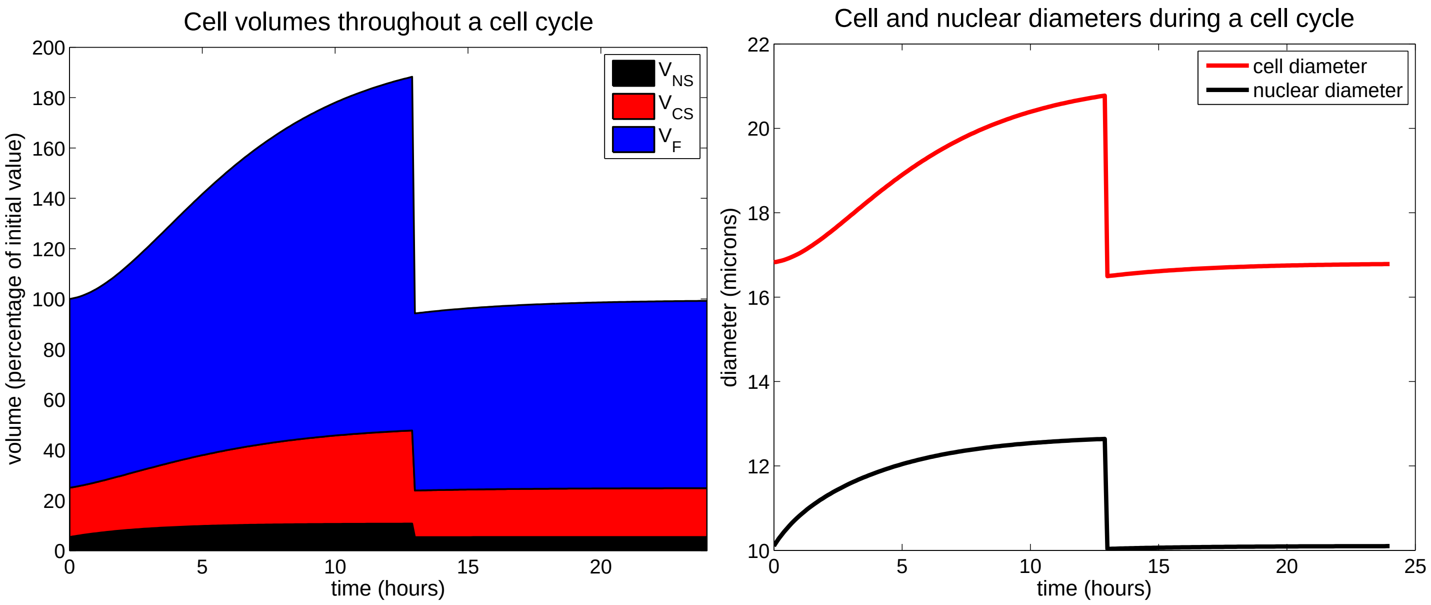

Figure 1. Cell volume during a (Ki67 Advanced) cell cycle: left: The fluid (blue), cytoplasmic solid (red), and nuclear solid (black) volumes increase prior to cell division, (here at 13 hours). The daughter cells continue growing after division until they reach their target volumes. right: The nuclear diameter (black curve) and total cell diameter (red curve) change throughout the cycle as well.

1.1.1 Reference parameter values

We estimate parameter values for breast epithelial cells (e.g,. similar to MCF-10A: a widespread, moderately malignant human breast cancer line), to be of comparable size to mammalian epithelial cells. For MCF-10A cells, $V_{\mathrm{N}} \approx 540\ \mu\mathrm{m}^3$, $V \approx 2494\ \mu\mathrm{m}^3$ [49], and so $V_{\mathrm{C}} \approx 1954\ \mu\mathrm{m}^3$, and $f_{\mathrm{CN}} \sim 3.6$.

Next, we estimate the water fraction f_{F}. A typical cell water mass fraction f_{M} is 70% (1 - dry mass / wet mass) [1, 3]. (See BNID 105938, 103960 [40].) To get the cell water volume fraction f_{F}, let ρ be the mass density of water, and ρ_{S} be the (relative) mass density of the solid cell fraction. Let V_{S} denote the total solid cell volume, so $V = V_{\mathrm{F}} + V_{\mathrm{S}}$. Typically, $\rho_{\mathrm{S}} \sim 1.3\rho$ [25, 39]. (See BNID 104272, 103206 [40].) Using these numbers, we can solve for V_{S}. If M_{F} is a cell's total fluid mass, and M_{S} is its total solid mass, then

$$f_{\mathrm{M}} = \frac{M_{\mathrm{F}}}{M_{\mathrm{F}} + M_{\mathrm{S}}} = \frac{\rho V_{\mathrm{F}}}{\rho V_{\mathrm{F}} + \rho_S V_{\mathrm{S}}} = \frac{\rho V_{\mathrm{F}}}{\rho V_{\mathrm{F}} + 1.3\rho V_{\mathrm{S}}} \Longrightarrow V_{\mathrm{S}} = \frac{1 - f_{\mathrm{M}}}{1.3 f_{\mathrm{M}}} V_{\mathrm{F}}. \tag{9}$$

Next,

$$f_{\mathrm{F}} = \frac{V_{\mathrm{F}}}{V_{\mathrm{F}} + V_{\mathrm{S}}} \Longrightarrow f_{\mathrm{F}} = \frac{1}{1 + \frac{1 - f_{\mathrm{M}}}{1.3 f_{\mathrm{M}}}}. \tag{10}$$

Using $f_{\mathrm{M}} = 0.70$, this gives $f_{\mathrm{F}} \sim 0.75$. Moreover we calculate $V_{\mathrm{NS}}^* = (1 - f_{\mathrm{F}})V_{\mathrm{N}} \approx 135\ \mu\mathrm{m}^3$

For the rate parameters, we set r_{N} so that most, or 95%, (we define "most" or "mostly" to mean reaching 95% of a target or goal, in the range of 90% to 99%) of nuclear solid doubling is complete within a typical 9 hour S-phase [8, 23, 40, 47]:

$$r_{\mathrm{N}} = -\frac{\ln 0.05}{9\ \mathrm{hour}} \approx 0.33\ \mathrm{hour}^{-1}. \tag{11}$$

To estimate the cytoplasmic solid biomass creation rate r_{C}, we set it sufficiently large to ensure that the biomass is 95% ("mostly") doubled within a short cell cycle. For consistency, we use reference values from the Ki-67 Advanced model; see Section 1.2.3. In that model, the cycle duration is at least 15.5 hours (the time during which cells stain positive for the Ki-67 protein; see Section 1.2.3). Because cytoplasmic biomass creation lags nuclear biomass creation in this model (which happens primarily in S phase), we set r_{C} fast enough to ensure that 95% of the biomass is created within the last half of the S-phase and the remainder of this minimal cell cycle duration. If T_{C} denotes this time scale, we set $T_{\mathrm{C}} = 15.5 - \frac{1}{2}T_{\mathrm{S}} = 11$ hours.

The analytical solution to Equation 3 (with $V_{\mathrm{CS}}^* = 2$ and $V_{\mathrm{CS}}(0) = 1$) gives

$$1 + .95 = 2 - (2 - 1)\mathrm{e}^{-r_{\mathrm{C}} T_{\mathrm{C}}} \Longrightarrow r_{\mathrm{C}} \approx -\frac{\ln(0.05)}{T_{\mathrm{C}}} \approx 0.27\ \mathrm{hour}^{-1}. \tag{12}$$

Table 1. Reference parameter values for the volume model, for breast epithelial cells.

Parameter	Biophysical meaning	Reference value
V	total cell volume	2494 μm^3
$V_{\rm N}$	total nuclear volume	540 μm^3
$r_{\rm F}$	rate of water intake (or release)	3.0 hour^{-1}
$r_{\rm C}$	rate of cytoplasmic solid biomass creation	0.27 hour^{-1}
$r_{\rm N}$	rate of nuclear biomass creation	0.33 hour^{-1}
$V^*_{\rm NS}$	"target" nuclear solid volume	135 μm^3
$f_{\rm CN}$	"target" cytoplasmic:nuclear volume ratio	3.6
$f_{\rm F}$	"target' water fraction	0.75

Lastly, we set the water transport parameter $r_{\rm F}$ sufficiently large that it "keeps pace" with biomass creation with a time scale $T_{\rm F}$. Since the time scales for biomass creation are on the order of 10 hours (see the discussion of $r_{\rm C}$ and $r_{\rm N}$ above), we estimate $T_{\rm F}$ to be one order of magnitude faster, or $T_{\rm F} \sim 1$ hour. If we let $W = V^*_{\rm F} - V_{\rm F}(0)$ and assume that $V^*_{\rm F}$ changes relatively slowly compared to $V_{\rm F}$, then the solution to Equation 1 can be approximated as $W(t) \approx W(0)e^{-r_{\rm F}t}$. Assuming that $W(T_{\rm F}) = 0.05W(0)$ (so that 95% of the targeted water flux occurs within the time scale $T_{\rm F}$),

$$0.05 = e^{-r_{\rm F}T_{\rm F}} \Longrightarrow r_{\rm F} = -\frac{\log(0.05)}{T_{\rm F}} \approx 3.0\,\text{hour}^{-1}. \tag{13}$$

We note that this parameter could be lower while still ensuring that the cell successfully doubles its total volume within a cell cycle of duration T. The smaller $r_{\rm F}$ is, the greater the variation in the water fraction (and hence the cell's density relative to water) throughout the cycle. This could be used to further constrain $r_{\rm F}$ using experimental techniques such as Byun et al. [7].

1.2 Cell cycle models

In the first release of PhysiCell, we provide cell cycle models for use with Ki-67 proliferation data (the "Ki-67 Advanced" and "Ki-67 Basic" models), and overall cell tracking ("Live Cells" model). Cells in any of these models are permitted to undergo one or more types of death according to user-supplied death rates. See Section 1.3 for supported cell death models.

1.2.1 General representation of a cell cycle model

In PhysiCell, a cell cycle model is a collection of phases $\{X_1, \ldots, X_n\}$ and transition rates $\{r_{ij}\}^n_{i,j=1}$, where r_{ij} is the transition rate from the $i^{\rm th}$ phase to the $j^{\rm th}$ phase. (Note that r_{ij} may be zero for many pairs of phases.) Some phase transitions may change $V^*_{\rm NS}$ and the sub-volume values. Each cycle model must include a cell division instruction during one phase-to-phase transition. Each cell agent k has a phenotypic phase $\mathcal{S}_k(t) \in \{X_i\}^n_{i=1}$, and t_k: the cumulative time spent in its current phase. As in our prior work [35], in any time interval $[t, t + \Delta t]$ a cell with $\mathcal{S}_k(t) = X_i$ has a probability of exiting the X_i phase and entering the X_j phase given by

$$\text{Prob}\Big(\mathcal{S}_k(t+\Delta t) = X_j | \mathcal{S}_k(t) = X_i\Big) \quad = \quad 1 - \exp\left(-r_{ij}\Delta t\right) \approx r_{ij}\Delta t. \tag{14}$$

Users can change r_{ij} at any time, in part based upon microenvironmental conditions. For example, r_{ij} may increase with oxygenation, and decrease with cell contact. If a phase X_i has only one nonzero r_{ij}, then $1/r_{ij}$ is the mean time spent in X_i [35]. In some models, cells can deterministically transition from X_i to X_j when $t_k \geq 1/r_{ij}$.

As of Version 1.2.0, users can specify "arrest conditions" for any transition rate r_{ij}, such that when the arrest condition is true (e.g., a cell volume checkpoint), transitions from X_i to X_j are not permitted. Moreover, users can assign any phase X_i an "entry function" (e.g., parameter mutations) that is evaluated whenever the cell enters phase X_i. See the user manual for more details.

1.2.2 Cell division

Common to all the cell cycle models is the process of cell division. When a cell agent k divides, we:

1. Divide all its sub-volumes in half.
2. Reset its elapsed phase time t_k to 0.

3. Duplicate the cell (including all state and parameter values).
4. **Place the cell and its duplicate:** For this step, let $0 \le p \le 1$ be the degree of polarization (fully polarized if $p = 1$, completely random orientation if $p = 0$), and let $\boldsymbol{\theta}$ be the cell's unit orientation vector (directed from cell base to cell apex). Let $\mathbf{r} \in [-1,1] \times [-1,1] \times [-1,1]$ be a random vector. Define

$$\mathbf{d} = \frac{\overbrace{\mathbf{r} - (\mathbf{r}\cdot\boldsymbol{\theta})\,\boldsymbol{\theta}}^{\text{perp. to }\boldsymbol{\theta}} + \overbrace{((1-p)\,(\mathbf{r}\cdot\boldsymbol{\theta}))\,\boldsymbol{\theta}}^{\text{parallel to }\boldsymbol{\theta}}}{||\mathbf{r} - (\mathbf{r}\cdot\boldsymbol{\theta})\,\boldsymbol{\theta} + ((1-p)\,(\mathbf{r}\cdot\boldsymbol{\theta}))\,\boldsymbol{\theta}||} \tag{15}$$

Notice that if $p = 1$ (a fully polarized cell), then $\mathbf{d}$ is a random unit vector perpendicular to $\boldsymbol{\theta}$, and if $p = 0$ (an unpolarized cell), then $\mathbf{d}$ is a random unit vector in 3-dimensional space. 2-D simulations (e.g., monolayer growth) should set $\boldsymbol{\theta} = [0, 0, 1]$.

If V is the volume of the parent cell at the time of division, with equivalent radius R, then the daughter cells have equivalent radius $\frac{1}{\sqrt[3]{2}}R$. Let $\mathbf{x}_{\text{parent}}$ be the center of the parent cell. Then we place the two daughter cells at

$$\mathbf{x}_{\text{daughters}} = \mathbf{x}_{\text{parent}} \pm \left(R - \frac{1}{\sqrt[3]{2}} R \right) \mathbf{d}. \tag{16}$$

1.2.3 Ki67 Advanced Model

This cell cycle model, which refines our prior work [13, 35, 27], was designed for matching simulations with clinical and experimental pathology data using Ki-67, a nuclear stain for cell proliferation [5, 50, 2, 37]. In this model, cycling cells (in phase K_1 with mean duration T_1) stain positive for Ki67 prior to proliferation, and for a short time after mitosis (in phase K_2 with mean duration T_2). Cells in the K_2 phase return to a Ki67 negative, quiescent phase (Q, with mean duration T_{Q}). Any of the cell cycle time scales can be adjusted (e.g., to reflect microenvironmental influences) at any time, on an individual cell basis.

Table 2. Ki67 Advanced cell cycle model: Reference parameter values.

Parameter	Biophysical meaning	Reference value
T_1	duration of K_1	13 hours
T_2	duration of K_2	2.5 hours
T_{Q}	duration of Q	3.62 hours

Q phase: In any time interval $[t, t + \Delta t]$, cell k has a probability of entering the K_1 phase given by

$$\text{Prob}\left(\mathcal{S}_k(t+\Delta t) = K_1 | \mathcal{S}_k(t) = Q\right) = 1 - e^{-\frac{1}{T_{\text{Q}}}\Delta t} \approx \frac{\Delta t}{T_{\text{Q}}}. \tag{17}$$

Upon entering the K_1 phase, we reset the phase time t_k, double V^*_{NS}, and leave all other parameters unchanged. For any death type D with death rate r_{D}, the probability of entering that death phase is given by

$$\text{Prob}\left(\mathcal{S}_k(t+\Delta t) = D | \mathcal{S}_k(t) = Q\right) = 1 - e^{-r_{\text{D}}\Delta t} \approx r_{\text{D}}\Delta t. \tag{18}$$

In PhysiCell, we use the linear approximations.

K_1 phase: In any time interval $[t, t + \Delta t]$, cell k has a probability of leaving the K_1 phase and entering the K_2 phase given by

$$\text{Prob}\left(\mathcal{S}_k(t+\Delta t) = K_2 | \mathcal{S}_k(t) = K_1\right) = 1 - e^{-\frac{1}{T_1}\Delta t} \approx \frac{\Delta t}{T_1}. \tag{19}$$

Upon exiting the K_1 phase, we halve V^*_{NS}, set $\mathcal{S}_k = K_2$, and divide the cell as in Section 1.2.2. For any death type D with death rate r_{D}, the probability of entering that death state is given by

$$\text{Prob}\left(\mathcal{S}_k(t+\Delta t) = D | \mathcal{S}_k(t) = K_1\right) = 1 - e^{-r_{\text{D}}\Delta t} \approx r_{\text{D}}\Delta t. \tag{20}$$

In PhysiCell, we use the linear approximations.

K_2 phase: In any time interval $[t, t+\Delta t]$, cell k has a probability of leaving the K_2 phase and entering the Q phase given by

$$\text{Prob}\left(\mathcal{S}_k(t+\Delta t) = Q | \mathcal{S}_k(t) = K_2\right) = 1 - e^{-\frac{1}{T_2}\Delta t} \approx \frac{\Delta t}{T_2}. \tag{21}$$

Upon exiting K_2 state, we set $\mathcal{S}_k = Q$ and reset the elapsed phase time t_k. For any death type D with death rate r_{D}, the probability of entering that death state is given by

$$\text{Prob}\left(S(t+\Delta t) = D | S(t) = K_2\right) = 1 - e^{-r_{\mathrm{D}}\Delta t} \approx r_{\mathrm{D}}\Delta t. \tag{22}$$

In PhysiCell, we use the linear approximations.

Reference parameter values: Ki-67 is expressed in the S, G_2, and M phases (the K_1 state in this model) [5], and to a lesser extent in the G_1 phase [50]. Ki-67 is seen in post-mitotic daughter cells (state K_2) [2], but it is not produced in these cells [5]. Instead, any remaining Ki-67 protein in post-mitotic cells is degraded quickly, with a half-life of 60-90 minutes [5]. Thus, we set T_1 to be the combined duration of S, G_2, and M, which are relatively fixed compared to the duration of G_0/G_1 ($T_2 + T_{\mathrm{Q}}$ in the Ki-67 advanced model) [32, 33]. As an estimate, we set $T_1 = 13$ hours, based upon typical estimates for the S, G_2, and M phases for eukaryotic cells [8]. ([8] estimates $S + G_2 + M$ is 9 hours, [47] estimates $S + G_2 + M$ is 12.3 hours, and [23] estimates $S + G_2 + M$ is approximately 13.1 hours.) We set T_2 to be on the order of two Ki-67 half-lives (we use an intermediate estimate of a 75 minute half-life), or 2.5 hours. We set $T_{\mathrm{Q}} = 3.62$ hours for consistency with the Live cycle model (below), which was calibrated to match MCF-10A measurements with a 2% apopotic fraction. See the user manual for more.

Variant model (1): We also provide the "Ki67 Advanced-Deterministic" model, where K_1 has fixed duration T_1, K_2 has fixed duration T_2, and Q has fixed duration T_{Q}. Note that if users do not initialize cells in Q with a distribution of elapsed times t_k, and if the durations are not microenvironment-dependent, then cells using this model will remain synchronized.

Variant model (2): To maintain compatibility with our older models [35, 27], we also provide the "Ki67 Advanced-Legacy" model, where K_1 has fixed duration T_1, K_2 has fixed duration T_2, but exiting the Q phase is a stochastic process with mean waiting time (duration) T_{Q}.

1.2.4 Ki67 Basic Model

To address circumstances where cell segmentation software or scientific instruments cannot distinguish between pre-mitotic and post-mitotic Ki67 positive cells, we provide a cell cycle model that combines K_1 and K_2 cells into a single Ki-67 positive phase K with mean duration T_{K}. Cells leaving the K phase divide into two Ki67 negative cells in the Q phase. Those cells remain in the Q phase for an average T_{Q} time. Any of the cell cycle time scales can be adjusted (e.g., to reflect microenvironmental influences) at any time, on an individual cell basis.

Table 3. Ki-67 Basic cell cycle model: Reference parameter values.

Parameter	Biophysical meaning	Reference value
T_{K}	duration of K	15.5 hours
T_{Q}	duration of Q	4.59 hours

Q phase: In any time interval $[t, t+\Delta t]$, cell k has a probability of entering the K phase given by

$$\text{Prob}\left(\mathcal{S}_k(t+\Delta t) = K | \mathcal{S}_k(t) = Q\right) = 1 - e^{-\frac{1}{T_{\mathrm{Q}}}\Delta t} \approx \frac{\Delta t}{T_{\mathrm{Q}}}. \tag{23}$$

Upon entering the K phase, we set $\mathcal{S}_k = K$, reset the state time $t_k = 0$, double V^*_{NS}, and leave all other parameters unchanged. For any death type D with death rate r_{D}, the probability of entering that death state is given by

$$\text{Prob}\left(\mathcal{S}_k(t+\Delta t) = D | \mathcal{S}_k(t) = Q\right) = 1 - e^{-r_{\mathrm{D}}\Delta t} \approx r_{\mathrm{D}}\Delta t. \tag{24}$$

In PhysiCell, we use the linear approximations.

K phase: In any time interval $[t, t+\Delta t]$, cell k has a probability of exiting the K phase and entering the Q phase given by

$$\text{Prob}\left(\mathcal{S}_k(t+\Delta t) = Q | \mathcal{S}_k(t) = K\right) = 1 - e^{-\frac{1}{T_\text{K}}\Delta t} \approx \frac{\Delta t}{T_\text{K}}. \tag{25}$$

Upon exiting K phase, we halve V^*_NS, set $\mathcal{S}_k = Q$ and $t_k = 0$, and divide the cell as in Section 1.2.2. For any death type D with death rate r_D, the probability of entering that death phase is given by

$$\text{Prob}\left(S(t+\Delta t) = D | S(t) = K\right) = 1 - e^{-r_\text{D}\Delta t} \approx r_\text{D}\Delta t. \tag{26}$$

In PhysiCell, we use the linear approximations.

Reference parameter values: For consistency with the Ki67 Advanced model, we set $T_K = T_1 + T_2 = 13.5$ hours, and we set $T_\text{Q} = 4.59$ for consistency with the Live cycle model (below), which was calibrated to match MCF-10A measurements with a 2% apopotic fraction. See the user manual for more.

Variant model (1): We also provide the "Ki67 Basic-Deterministic" model, where K has fixed duration T_K, and Q has fixed duration T_Q. Note that if users do not initialize cells in Q with a distribution of elapsed times t_k, and if the durations are not microenvironment-dependent, then cells using this model will remain synchronized.

Variant model (2): To maintain compatibility with our older models [35, 27], we also provide the "Ki67 Basic-Legacy" model, where K has fixed duration T_K, but exiting the Q phase is a stochastic process with mean waiting time (duration) T_Q.

1.2.5 Live Cells model

In this simpler model, we simply track live cells with phase L. In any $[t, t+\Delta t]$, a live cell can proliferate with probability

$$\text{Prob}\left(\text{division during } [t, t+\Delta t]\right) = 1 - e^{-b\Delta t} \approx b\Delta t. \tag{27}$$

We divide the cell as in Section 1.2.2. For any death type D with death rate r_D, the probability of entering that death phase is given by

$$\text{Prob}\left(S(t+\Delta t) = D | S(t) = L\right) = 1 - e^{-r_\text{D}\Delta t} \approx r_\text{D}\Delta t. \tag{28}$$

In PhysiCell, we use the linear approximations.

Table 4. Live cell cycle model: Reference parameter values.

Parameter	Biophysical meaning	Reference value
b	cell birth (division) rate	0.0432 hour^{-1}

Reference parameter values: In the user manual, we fitted $b = 0.0432\ \text{hr}^{-1}$ to match experimental values for MCF-10A with a 2% apoptotic index.

1.3 Cell death models

PhysiCell has been designed to support multiple models of cell death. In its current release, we support apoptosis and necrosis. Autophagy may be supported in a future release.

1.3.1 Apoptosis

Upon entering the apoptosis model (phase A), we set $V^*_\text{NS} = 0$, $f_\text{CN} = 0$, and $f_\text{F} = 0$. The rate parameters r_N, r_C, and r_F should be reset with apoptosis-specific rates to reflect the timescales of nuclear degradation, water loss, and cytoplasmic blebbing. See Reference parameter values: below. The cell should be removed from the simulation after T_A, the duration of apoptosis, or once a cell reaches a threshold volume. The model is illustrated in Figure 2 using the reference parameter values below.

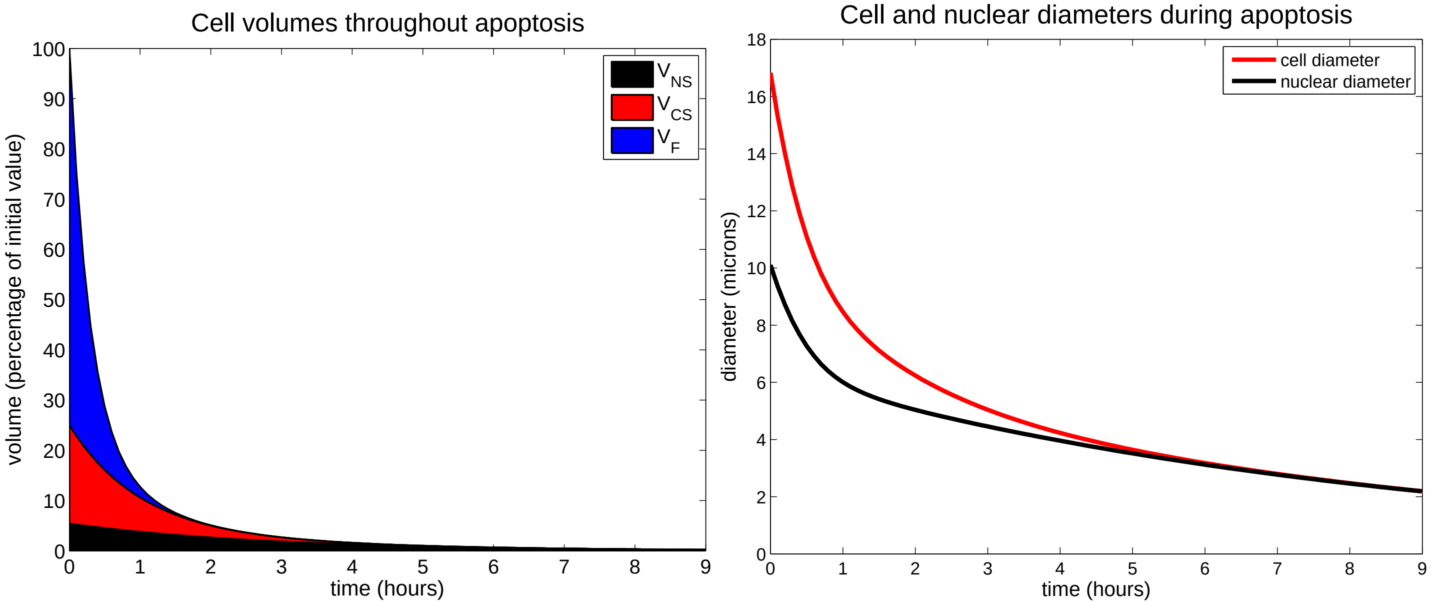

Figure 2. Cell volume during apoptosis: left: The fluid (blue), cytoplasmic solid (red), and nuclear solid (black) volumes decrease according to different time scales, giving an overall volume decrease that does not fit a simple exponential decrease. right: The nuclear diameter (black curve) and total cell diameter (red curve) initially decrease rapidly as water is removed from the cell. Later, the nuclear diameter dominates the cell after much of the cytoplasm has been lost (e.g., by blebbing).

Reference parameter values: In [35, 36], we estimated the time scales for apoptosis. We continue to use $T_{\mathrm{A}} = 8.6$ hour. Water loss occurs relatively quickly, with most water actively removed from the cell on the order of 1 hour. We set r_{F} such that 95% of water is removed by 1 hour:

$$r_{\mathrm{F}} = -\frac{\ln(0.05)}{1 \text{ hour}} \approx 3.0 \text{ hour}^{-1}. \tag{29}$$

Cytoplasmic blebbing is also relatively fast, occurring on the order of 3 hours. We therefore set

$$r_{\mathrm{C}} = -\frac{\ln(0.05)}{3 \text{ hour}} \approx 1.0 \text{ hour}^{-1}. \tag{30}$$

The nucleus is degraded more slowly over the duration T_{A} of apoptosis, and so we set

$$r_{\mathrm{N}} = -\frac{\ln(0.05)}{8.6 \text{ hour}} \approx 0.35 \text{ hour}^{-1}. \tag{31}$$

We note that *in vitro* time course measurements for apoptotic cells are increasingly available. [29] found that apoptotic KB cells lose approximately one third of their volume in 45-60 minutes, and 60% of their volume in less than 4 hours. [42] observed a 50% cell volume loss in apoptotic NIH 3T3 mouse embryonic fibroblasts 100 minutes after exposure to 10 μM of cadmium, with most of this volume loss in the last 20 minutes. These time scales are of comparable magnitude to these reference parameter values.

Lastly, we note that cell apoptosis may demonstrate different behaviors when triggered by therapeutic agents than by intrinsic processes (e.g., failing a cell checkpoint). See, for instance, the radical cell swelling reported in [14] following etopside exposure, reminiscent of necrotic death (see Section 1.3.2). Future versions of PhysiCell may include additional apoptosis models to account for multiple types of cell death.

1.3.2 Necrosis

Expanding the necrosis model in Macklin et al. [35], any cell (in any cell cycle model) can enter the necrotic state N with rate r_{nec}. In any time interval $[t, t + \Delta t]$, the probability of becoming necrotic is

$$\text{Prob}\left(S(t+\Delta t) = N | S(t) \neq N\right) = 1 - e^{-r_{\mathrm{nec}}\Delta t} \approx r_{\mathrm{necrosis}}\Delta t. \tag{32}$$

The necrosis death rate parameter should be set dynamically, according to the cell's energy status (e.g., as in [17]) or its microenvironmental conditions. (See below.) Necrotic cells initially swell by oncosis, rupture, and slowly degrade. Necrotic cells may optionally calcify, but this option is not active by default.

Oxygen-dependent necrotic death rate: In PhysiCell, by default we provide the following constitutive law for the necrotic death rate:

$$r_{\text{necrosis}} = \begin{cases} r_{\text{nec}}^{\text{crit}} & \text{if } \mathrm{pO}_2 < \mathrm{pO}_{2,\text{crit}} \\ r_{\text{nec}}^{\text{crit}} \left(\frac{\mathrm{pO}_{2,\text{thresh}} - \mathrm{pO}_2}{\mathrm{pO}_{2,\text{thresh}} - \mathrm{pO}_{2,\text{crit}}} \right) & \text{if } \mathrm{pO}_{2,\text{crit}} \le \mathrm{pO}_2 \le \mathrm{pO}_{2,\text{thresh}} \\ 0 & \text{if } \mathrm{pO}_{2,\text{thresh}} < \mathrm{pO}_2 \end{cases} \tag{33}$$

Early necrosis (oncosis): Upon entering the necrotic state, set the cell phase elapsed time to 0, set the phenotypic cycle/death phase to N, and change $f_{\text{F}} = 1$ (for cell swelling by osmosis). Set $f_{\text{CN}} = 0$ (for cytoplasmic degradation) and $V_{\text{NS}}^* = 0$ (for nuclear degradation). The rate parameters r_{F}, r_{N}, and r_{C} should be set to necrosis-specific values; see the reference values below. Lastly, set $V_{\text{rupture}} > V(0)$, where $V(0)$ is the cell volume at the start of necrosis. Once $V > V_{\text{rupture}}$, set the Boolean variable to `is_lysed` to true, and proceed to late necrosis (below). This model is plotted in Figure 3 with reference parameter values (see Reference parameter values).

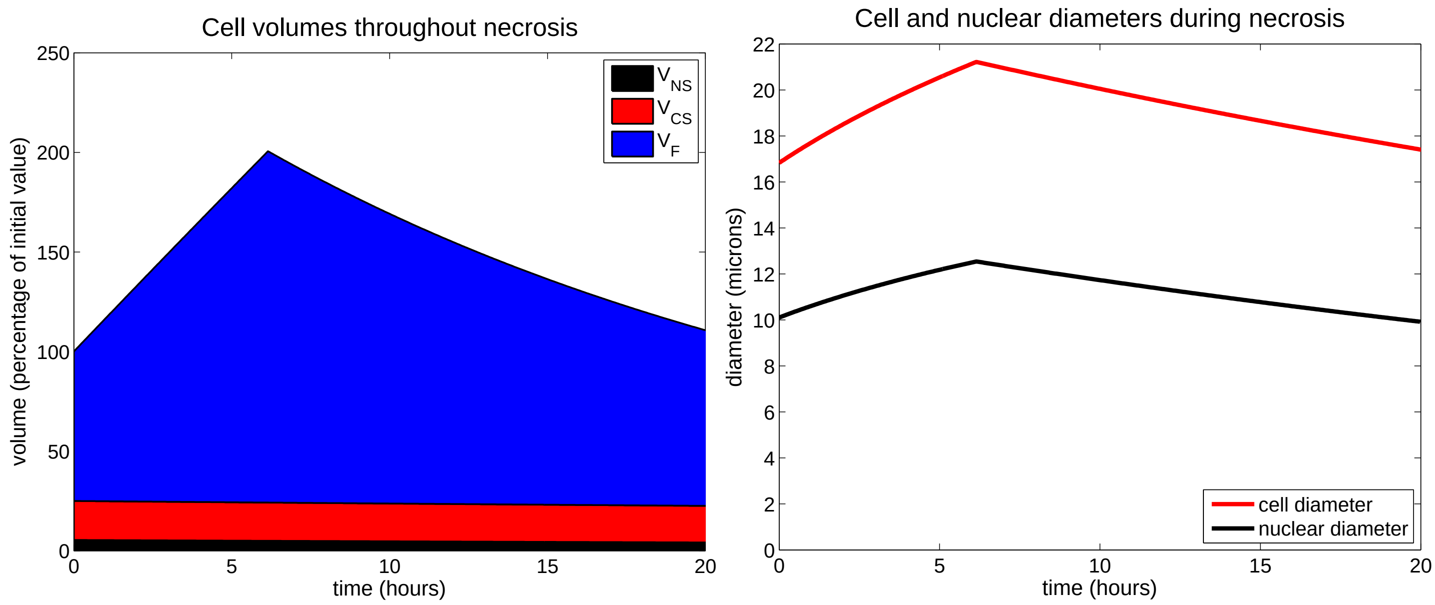

Figure 3. Cell volume during early necrosis: left: The fluid volume (blue) initially swells due to osmosis (oncosis), until the cell bursts around 6 hours. After that time, fluid slowly leaks out, while the nuclear solids (black) degrade (pyknosis), and the cytoplasmic solids (red) degrade and (optionally) calcify (yellow). right: The nuclear diameter (black curve) and total cell diameter (red curve) during early necrosis.

Late necrosis (after oncosis and lysis): Set $f_{\text{F}} = 0$, and reset r_{F} as discussed below. Optionally remove the cell from the simulation if its volume falls below a threshold (e.g., 1 μm^3). This model is plotted in Figure 4 with reference parameter values (see Reference parameter values).

(Dystrophic) cell calcification In [35], we introduced a simple model of cell calcification, where necrotic cells transform to calcified necrotic cell debris at a constant rate. We now improve the biorealism of that model. In calcification, necrotic debris interacts with calcium in the microenvironment to form calcium phosphate or calcium oxalte crystals in the cytoplasm [31, 26]. We denote calcified cytoplasmic solid volume by V_{CC}, and we model this conversion of uncalcified cytoplasmic material into calcified material as a set of reaction equations

(which modify Eqation 3 while setting $f_{\mathrm{CN}} = 0$ so that $V^*_{\mathrm{CS}} = 0$):

$$\frac{d}{dt} V_{\mathrm{CS}} = -r_{\mathrm{C}} V_{\mathrm{CS}} - r_{\mathrm{calc}} V_{\mathrm{CS}} \quad (34)$$

$$\frac{d}{dt} V_{\mathrm{CC}} = r_{\mathrm{calc}} V_{\mathrm{CS}} - r_{\mathrm{C}} V_{\mathrm{CC}}. \quad (35)$$

(Here, we have assumed that calcified and uncalcified necrotic cytoplasmic debris are degraded at the same rate.) Let $V_{\mathrm{C}} = V_{\mathrm{CS}} + V_{\mathrm{CC}}$, and define $f_{\mathrm{C}} = V_{\mathrm{CC}}/V_{\mathrm{C}}$ to be calcified fraction of the cytoplasmic solid biomass. Then

$$\frac{df_{\mathrm{C}}}{dt} = \frac{V_C \frac{d}{dt} V_{\mathrm{CC}} - V_{\mathrm{CC}} \frac{d}{dt} V_C}{V_{\mathrm{C}}^2} \quad (36)$$

$$= r_{\mathrm{calc}} \left(1 - f_{\mathrm{C}}\right). \quad (37)$$

The model is plotted in Figure 4 with reference parameter values (see Reference parameter values).

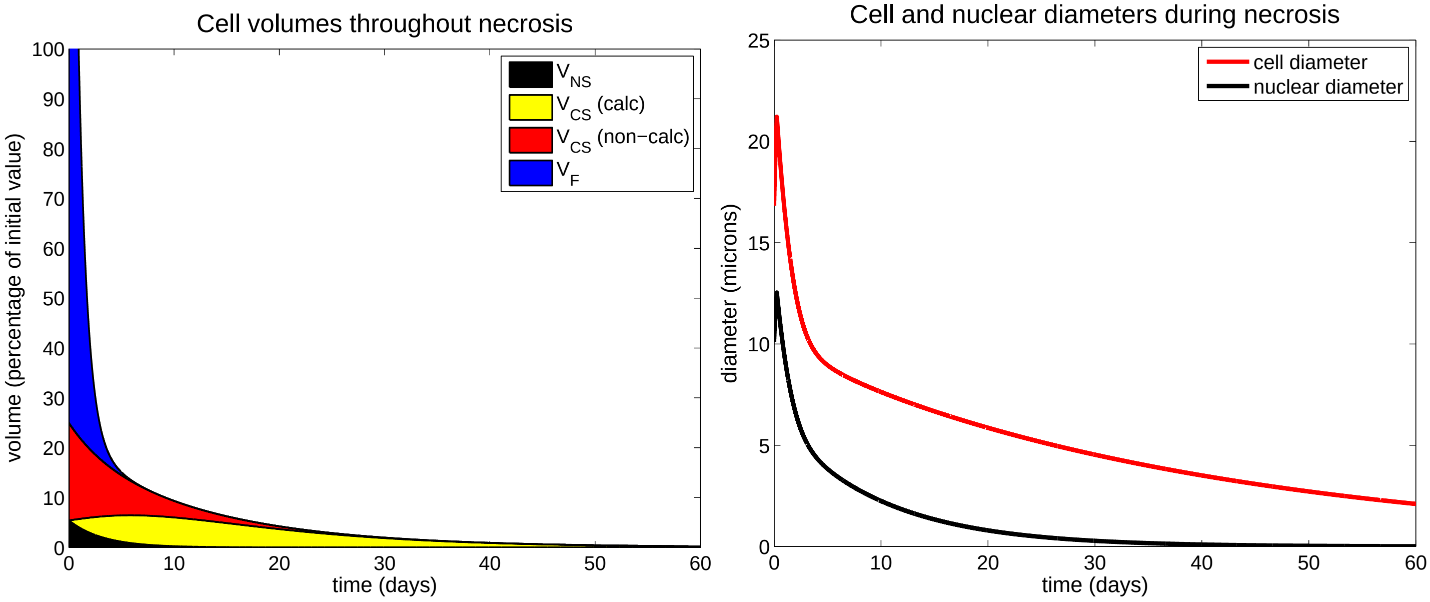

Figure 4. Cell volume during late necrosis: left: The fluid volume (blue) initially swells due to osmosis (oncosis), until the cell bursts around 6 hours. After that time, fluid slowly leaks out, while the nuclear solids (black) degrade (pyknosis), and the cytoplasmic solids (red) degrade and (optionally) calcify (yellow). right: The nuclear diameter (black curve) and total cell diameter (red curve) during early necrosis.

Reference parameter values In [35, 36], we estimated the magnitude for several key necrosis and calcification time scales. Let us summarize and extend those estimates here. To begin, we define the following time scales:

T_{swell}: The time scale for cell swelling during early necrosis (oncosis).

T_{shrink}: The time scale for water loss after cell lysis.

T_{CD}: The time scale for cytoplasmic degradation.

T_{ND}: The time scale for nuclear degradation (pyknosis).

T_{calc}: The time scale for significant calcification.

The early time scale estimates in Macklin et al. [35, 36] were based in part upon observations from hematoxylin and eosin (H&E) stained necrotic (and sometimes calcified) tissues, such as ductal carcinoma in situ. (See [11, 20, 22, 35, 36, 44] for some sample images of comedonecrosis in breast, prostate, and other carcinomas.) Intensely-stained pink (eosin) necrotic cells are observed near the outer edges of necrotic cores with the newest necrotic material, suggesting significant water loss to concentrate the cytoplasm early in necrosis. These cells generally have intact purple (hematoxylin) nuclei, suggesting that $T_{\mathrm{shrink}} < T_{\mathrm{ND}}$. Farther from the edges of

necrotic tissues, bright pink tissues with increasingly small or absent nuclei are observed, leading us to estimate that nuclear degradation is faster than cytoplasmic degradation ($T_{\rm ND} < T_{\rm CD}$). Moreover, calcified materials are generally observed within the most central, oldest portions of necrotic tissues where no nuclei are observed, and so we further estimate $T_{\rm ND} < T_{\rm calc} < T_{\rm CD}$. Thus,

$$T_{\rm swell} < T_{\rm shrink} < T_{\rm ND} < T_{\rm calc} < T_{\rm CD}. \tag{38}$$

As in prior work Macklin et al. [35], we estimate $T_{\rm swell} \approx 6$ hours, and that the cell fluid volume doubles in this time:

$$\frac{d}{dt}V_{\rm F} = r_{\rm F}\left(V_{\rm F}^{*}(t) - V_{\rm F}(t)\right). \tag{39}$$

Because the cell is swelling by oncosis, $f_{\rm F} = 1$, and so $V_{\rm F}^{*}(t) = 100\% V(t) = V_{\rm NS}(t) + V_{\rm CS}(t) + V_{\rm F}(t)$. By the estimates above, the time scales of necrotic and cytoplasmic degradation are slower than oncotic swelling. Therefore, we approximate (for early necrosis) $V_{\rm CS}(t) \approx V_{\rm CS}(0)$, $V_{\rm NS}(t) \approx V_{\rm NS}(0)$, and

$$\begin{aligned} V_{\rm F}^{*} &\approx V_{\rm CS}(0) + V_{\rm NS}(0) + V_{\rm F} && (40)\\ &= (1 - f_{\rm F}(0))V(0) + V_{\rm F}, && (41) \end{aligned}$$

and thus Equation 39 simplifies to

$$\begin{aligned} \frac{dV_{\rm F}}{dt} &= r_{\rm F}\left((1 - f_{\rm F}(0))\,V(0) + V_{\rm F} - V_{\rm F}\right) && (42)\\ &= r_{\rm F}\left(1 - f_{\rm F}(0)\right)V(0) && (43)\\ \Longrightarrow V_{\rm F}(t) &\approx V_{\rm F}(0) + r_{\rm F}\left(1 - f_{\rm F}(0)\right)V(0)t. && (44) \end{aligned}$$

Because $V_{\rm NS}$ and $V_{\rm CS}$ are approximately constant in early necrosis, we thus have

$$\frac{dV}{dt} \approx \frac{dV_{\rm F}}{dt} \quad \Longrightarrow \quad V(t) \approx V_{\rm NS}(0) + V_{\rm CS}(0) + V_{\rm F}(0) + r_{\rm F}\left(1 - f_{\rm F}(0)\right)V(0)t, \tag{45}$$

or after normalization by $V(0)$,

$$\frac{V(t)}{V(0)} \approx 1 + r_{\rm F}\left(1 - f_{\rm F}(0)\right)t. \tag{46}$$

Notice that the cells grow linearly, consistent with observations of apoptotic and necrotic blebbing [4]. We use this expression to estimate $r_{\rm F}$ prior to lysis, assuming that cell volume doubles after $T_{\rm swell} = 6$ hours' time ($V\left(T_{\rm swell}\right) = 2V(0)$) and using the reference parameter value $f_{\rm F}(0) = 0.75$ (see Reference parameter values):

$$r_{\rm F} = \frac{1}{\left(1 - f_{\rm F}(0)\right)T_{\rm swell}} \approx 0.67\ \text{hour}^{-1}, \tag{47}$$

We estimate that fluid loss after lysis is at least one order of magnitude slower than fluid gain during oncosis, and so $T_{\rm shrink} \sim 60$ hours. Solving Equation 1 with $V_F^{*} = 0$ and choosing $r_{\rm F}$ such that $V_{\rm F}(T_{\rm shrink}) = 0.05 V_{\rm F}(0)$, we have

$$r_{\rm F} = -\frac{\log 0.05}{60\ \text{hours}} \approx 0.050\ \text{hour}^{-1}. \tag{48}$$

To choose a reference value for the rate of nuclear degradation, we note that DNA has a 54-hour half-life in conditions of oxidative stress [53]. If we assume the nuclear structures degrade at similar rates, then

$$r_{\rm N} = \frac{\ln 2}{54\ \text{hours}} \approx 0.013\ \text{hour}^{-1}. \tag{49}$$

This rate gives approximately 95% degradation of nuclear solids in 10 days, and 99% within 15 days–consistent with our earlier estimates [35, 36] and the time scale estimates above.

In Macklin et al. [35], we discussed the time scale of calcification at length. We choose $r_{\rm calc}$ so that the cell is "mostly" (95%) calcified by 30 days:

$$r_{\rm calc} = -\frac{\log 0.05}{30 \cdot 24\ \text{hours}} \approx 0.0042\ \text{hour}^{-1}. \tag{50}$$

With this parameter value, the cell is significantly (50%) calcified in 7 days, 75% calcified by 14 days, 87% calcified by 21 days, and 95% calcified by 30 days. This is consistent with the parameter estimates in Macklin et al. [36].

To choose a reference value for the rate of cytoplasmic degradation, the literature reports that phospholipids (a major component of cell and organelle membranes) have a half-life of 100-300 hours [30]. This led us to further estimate that $T_{\mathrm{CD}} \sim 60$ days. We choose r_{C} such that 99% of the cytoplasm is degraded by T_{C} time. (If we use the 95% degradation standard for this parameter estimate, the remaining cell diameter at T_{C} days is over 3 μm–too large to regard as degraded within the specified time scale.) Thus, we estimate:

$$r_C = -\frac{\log 0.01}{60 \text{ days}} \approx 0.0032 \text{ hour}^{-1}. \tag{51}$$

This rate gives approximately 40% degradation of cytoplasmic solids in 10 days, 80% within 30 days, and 95% within approximately 55 days–consistent with our earlier estimate of 60 days in Macklin et al. [36].

1.4 Cell mechanics and motion

We model cell mechanics and motion as in our prior work [35]: each cell's position is updated based upon the balance of cell-cell and cell-microenvironment forces acting upon it. For cell i at position $\mathbf{x}_i(t)$, with velocity $\mathbf{v}_i(t)$, and with a set $\mathcal{N}(i)$ of nearby cells, we model

$$m_i \dot{\mathbf{v}}_i = \sum_{j \in \mathcal{N}(i)} \left(\mathbf{F}^{ij}_{\mathrm{cca}} + \mathbf{F}^{ij}_{\mathrm{ccr}} \right) + \mathbf{F}^{i}_{\mathrm{cba}} + \mathbf{F}^{i}_{\mathrm{cbr}} + \mathbf{F}^{i}_{\mathrm{drag}} + \mathbf{F}^{i}_{\mathrm{loc}} \tag{52}$$

where $\mathbf{F}_{\mathrm{cca}}$ and $\mathbf{F}_{\mathrm{ccr}}$ are cell-cell adhesive and "repulsive" forces, $\mathbf{F}_{\mathrm{cba}}$ and $\mathbf{F}_{\mathrm{cbr}}$ are cell-basement membrane adhesive and repulsive forces, $\mathbf{F}_{\mathrm{drag}}$ collects dissipative, drag-like forces (e.g., fluid drag and cell-matrix adhesion), and $\mathbf{F}_{\mathrm{loc}}$ is the net locamotive (motile) force. ("Repulsive" forces model elastic resistance to deformation.) As in [15, 34, 35],

$$\mathbf{F}^{i}_{\mathrm{drag}} \quad = \quad -\left(\nu + \nu_1 E_i\right) \mathbf{v}_i = -\nu_i \mathbf{v}_i, \tag{53}$$

where $E_i = E\left(\mathbf{x}_i\right)$ is the ECM density (or volume fraction) around the cell, and ν is a (fluid) drag coefficient. As in prior cell-centered models [12, 16, 35], we apply the inertialess assumption ($m_i \dot{\mathbf{v}}_i \approx \mathbf{0}$), which assumes that forces equilibrate at relatively fast time scales relative to the time scales of cell cycling, death, volume changes, and multicellular patterning. This allows us to explicitly solve for $\mathbf{v}_i$:

$$\mathbf{v}_i \quad = \quad \frac{1}{\nu_i} \left(\sum_{j \in \mathcal{N}(i)} \left(\mathbf{F}^{ij}_{\mathrm{cca}} + \mathbf{F}^{ij}_{\mathrm{ccr}} \right) + \mathbf{F}^{i}_{\mathrm{cba}} + \mathbf{F}^{i}_{\mathrm{cbr}} + \mathbf{F}^{i}_{\mathrm{loc}} \right). \tag{54}$$

We model $\mathbf{F}_{\mathrm{cca}}$, $\mathbf{F}_{\mathrm{cba}}$, $\mathbf{F}_{\mathrm{ccr}}$, and $\mathbf{F}_{\mathrm{cbr}}$ with interaction potentials that depend upon each cell's volume, maximum adhesion distance, adhesion and repulsion parameters, and distance to other cells. See Section 1.4.1.

1.4.1 Mechanics potential functions

Building upon our prior work in [35], we use potential functions ϕ for adhesive interactions, and potential functions ψ for "repulsive" interactions. For a good discussion of potential functions see [6]. For computing forces in PhysiCell, we only need to define the gradients of ϕ and ψ. For adhesion,

$$\nabla \phi_{n,R_{\mathrm{A}}}(\mathbf{r}) \quad = \quad \begin{cases} \left(1 - \frac{|\mathbf{r}|}{R_{\mathrm{A}}}\right)^{n+1} \frac{\mathbf{r}}{|\mathbf{r}|} & \text{if } |\mathbf{r}| \le R_{\mathrm{A}} \\ \mathbf{0} & \text{otherwise.} \end{cases} \tag{55}$$

Here, R_{A} is a maximum adhesive interaction distance, and n is an integer power (typically 1) chosen for the smoothness of the force's behavior as $r \to R_{\mathrm{A}}$; $n = 0$ gives minimal smoothness: continuity of the force itself, but not of any derivatives.

For replusion,

$$\nabla \psi_{n,R}(r) \quad = \quad \begin{cases} -\left(1 - \frac{|\mathbf{r}|}{R}\right)^{n+1} \frac{\mathbf{r}}{|\mathbf{r}|} & \text{if } |\mathbf{r}| \le R \\ \mathbf{0} & \text{otherwise.} \end{cases} \tag{56}$$

where n again gives the smoothness at the edge of interaction.

1.4.2 Cell-cell mechanics

Following our work in [35] and [9], we model the cell-cell adhesive force between cells i and j with individual adhesion parameters $R_{i,\mathrm{A}}$ and $R_{j,\mathrm{A}}$ as

$$\mathbf{F}_{\mathrm{cca}}^{ij} = -C_{\mathrm{cca}} A_i A_j \nabla \phi_{n_{\mathrm{cca}}, R_{i,\mathrm{A}}+R_{j,\mathrm{A}}} \left(\mathbf{x}_j - \mathbf{x}_i \right), \tag{57}$$

where C_{cca} is the cell-cell adhesion parameter, A_i and A_j are the cells' relative adhesiveness parameters ($0 \leq A_i \leq 1$), and n_{cca} is the cell-cell adhesion exponent parameter. By default $A_i = 1$ for all cells.

If cells i and j have radii R_i and R_j, respectively, then we model the cell-cell repulsive force as

$$\mathbf{F}_{\mathrm{ccr}}^{ij} = -C_{\mathrm{ccr}} \nabla \psi_{n_{\mathrm{cca}}, R_{i,\mathrm{A}}+R_{j,\mathrm{A}}} \left(\mathbf{x}_j - \mathbf{x}_i \right), \tag{58}$$

where C_{ccr} is the cell-cell repulsion parameter, and n_{ccr} is the cell-cell repulsion exponent parameter.

Note that Equation 58 does not explicitly account for differences in the cells' elastic moduli, whereby cell i and cell j should experience differing restorative forces. In a coming release, we shall include a slightly extended model that can account for interactions between cells with differing elastic moduli.

We note that these simple forces are selected primarily to model basic adhesion and repulsion, and they can be tuned to a target cell density; see the discussion in [35]. However, they neglect cell-cell frictional, tangential forces, and they have no hysteresis: they do not model the fact that the force of cell-cell adhesion likely varies in time, and in particular, cell-cell adhesions are likely created at shorter cell-cell distances while maintained at longer cell-cell distances prior to breaking. See the excellent discussion in [51]. PhysiCell's default cell-cell mechanics functions can be replaced with more biorealistic functions, and future releases will include a larger library of implemented forms.

1.4.3 Cell-BM mechanics

PhysiCell includes basic functions for mechanical interactions with the basement membrane. Like cell-cell mechanical interactions, they also neglect tangential effects. If the user supplies a function $d(\mathbf{x})$ which gives the distance from $\mathbf{x}$ to the basement membrane, as well as $\mathbf{n}(\mathbf{x}) = \nabla d(\mathbf{x})$: the unit normal vector facing away from the basement membrane, then we model the cell-BM interaction forces as in [35]:

$$\mathbf{F}_{\mathrm{cba}}^{i} = -C_{\mathrm{cba}} \nabla \phi_{n_{\mathrm{cba}}, R_{i,\mathrm{A}}} \Big(-d\left(\mathbf{x}_i\right) \mathbf{n}\left(\mathbf{x}_i\right) \Big) \tag{59}$$

$$\mathbf{F}_{\mathrm{cbr}}^{i} = -C_{\mathrm{cbr}} \nabla \psi_{n_{\mathrm{cba}}, R_{i}} \Big(-d\left(\mathbf{x}_i\right) \mathbf{n}\left(\mathbf{x}_i\right) \Big), \tag{60}$$

where n_{cba} and n_{cbr} are the cell-BM adhesion and repulsion exponents, and C_{cba} and C_{cbr} give the strength of the adhesive and repulsive interactions.

1.4.4 Motility

In addition to the forces due to interaction with other cells and BM, cells may demonstrate a net locomotive force to become motile. For example, in the case of chemotaxis, cells move in response to chemical stimuli, e.g., moving towards the areas with more oxygen or nutrients. PhysiCell currently implements biased random migration along $\mathbf{d}_{\mathrm{bias}}$ with bias $b \leq 0 \leq 1$. ($b = 0$ gives completely random motility, and $b = 1$ is completely deterministic motility along $\mathbf{d}_{\mathrm{bias}}$.) To make the motility function mathematically consistent across multiple values of Δt, we use the following procedure in each time interval $[t, t + \Delta t]$ to determine the contribution $\mathbf{F}_{\mathrm{loc}}$ to the cell's velocity:

1. Choose a random number (with uniform distribution) $0 \leq u \leq 1$
2. If $\xi \leq \frac{\Delta t}{T_{\mathrm{persistence}}}$ (the probability of changing direction), then choose a new direction $\mathbf{u}$, where $\mathbf{u}_{\mathrm{loc}}$ is a unit vector:
   (a) Choose a random unit vector $\boldsymbol{xi}$.
   (b) Set
   $$\mathbf{u}_{\mathrm{loc}} = \frac{b\mathbf{d}_{\mathrm{bias}} + (1-b)\boldsymbol{\xi}}{||b\mathbf{d}_{\mathrm{bias}} + (1-b)\boldsymbol{\xi}||} \tag{61}$$

   If $u > \frac{\Delta t}{T_{\mathrm{persistence}}}$, leave $\mathbf{u}_{\mathrm{loc}}$ unchanged. ($\mathbf{u}_{\mathrm{loc}}$ is initialized to $\mathbf{0}$ for each cell as it is created.)
3. Set
   $$\mathbf{F}_{\mathrm{loc}} = s_{\mathrm{loc}} \mathbf{u}_{\mathrm{loc}}, \tag{62}$$

   where s_{loc} is the cell's current (user-set) migration speed.

See the user manual for more information.

1.4.5 Cell orientation

In the initial release of PhysiCell, we include a placeholder for cell orientation: each cell has a unit vector $\boldsymbol{\theta}$ that gives the direction from its basal side to its apical side, and a polarity scalar $0 \le p \le 1$, where $p = 0$ is an unpolarized cell, and $p = 1$ is a polarized cell.

We do not at the present time include functions to maintain or update a cell's orientation; these are left to the users (e.g., see [43, 45]). Future releases of PhysiCell will include further default functionality.

However, PhysiCell does use $\boldsymbol{\theta}$ when placing daughter cells after cell division; see Section 1.2.2. Thus, users should set $\boldsymbol{\theta} = [0, 0, 1]$ and $p = 1$ for 2-D simulations, to ensure that daughter cells do not divide into the (non-physical) third dimension.

1.4.6 Reference parameter values

Following our work in [27], we include reference mechanics parameter values for breast epithelial cells, chosen to enforce a 90% confluent tissue (both 2D and 3D). The mechanics parameter values below are consistent with the reference volume parameters given in Section Reference parameter values.

Cell-cell mechanics Following [27], we estimate the cell density for an approximately 90% confluent tissue:

$$\rho = \frac{0.9}{A_{\mathrm{cell}}} \quad \Longrightarrow \quad \rho \approx 4.0 \times 10^{-3} \mu\mathrm{m}^{-2}, \tag{63}$$

where we used

$$A_{\mathrm{cell}} = \left(\frac{3\sqrt{\pi}}{4} V_{\mathrm{cell}} \right)^{\frac{2}{3}} \approx 222.3\,\mu\mathrm{m}^2, \tag{64}$$

and

$$R_{\mathrm{cell}} = \left(\frac{3\,V_{\mathrm{cell}}}{4\pi} \right)^{\frac{1}{3}} \approx 8.4\,\mu\mathrm{m}. \tag{65}$$

Next, we obtain the mean cell-cell spacing s for a tissue in (biomechanical) homeostasis. As in [27],

$$s \quad = \quad \sqrt{\frac{2 \cdot 0.9}{\sqrt{3}\rho}} \approx 16.0\,\mu\mathrm{m}. \tag{66}$$

We continue to use $C_{\mathrm{ccr}} = 10.0\nu\,\mu\mathrm{m/min}$ as in [35]. Similar to [35], we set

$$R_{\mathrm{A}} = 1.25\,R_{\mathrm{cell}} \approx 10.5\,\mu\mathrm{m} \tag{67}$$

to allow cell-cell interaction over slightly larger distances than in [35]; this value is consistent with cell deformation measurements for breast epithelial cells in [21].

Finally, as in [27], we set the cell-cell adhesive and repulsive forces at equilibrium at a separation of s to obtain C_{cca}:

$$\begin{aligned} C_{\mathrm{cca}} \left(1 - \frac{s}{2R_{\mathrm{A}}} \right)^2 &= C_{\mathrm{ccr}} \left(1 - \frac{s}{2R_{\mathrm{cell}}} \right)^2 \\ \Longrightarrow \quad C_{\mathrm{cca}} &= \left(\frac{1 - \frac{s}{2R_{\mathrm{cell}}}}{1 - \frac{s}{2R_{\mathrm{A}}}} \right)^2 C_{\mathrm{ccr}} \approx 0.04 C_{\mathrm{ccr}} \approx 0.4\nu\,\mu\mathrm{m/min}. \end{aligned} \tag{68}$$

We use the same parameters in 2D and in 3D, noting that the 2-D hexagonal close packing used to tie the (planar) cell density to the equilibrium cell spacing can be used for an analogous close cell packing in 3D with the same cell-cell spacing.

Cell-BM mechanics As in [35], we set $C_{\mathrm{cbr}} = C_{\mathrm{ccr}}$ and $C_{\mathrm{cba}} = 10\,C_{\mathrm{cca}}$. See Table 6.

Cell motility In [52], it was determined that $T_{\mathrm{persistence}} \sim 3$ to 30 minutes, so we set $T_{\mathrm{persistence}} = 15$ min. For the migration speed, [10, 38] found that breast epithelial cells migrate at speeds of $s_{\mathrm{loc}} \sim 0.3$ to 1.0 μm/min, so we set $s_{\mathrm{loc}} = 0.5\ \mu$m/min. See Table 7.

Cell polarity By default, PhysiCell works in 3D with unpolarized cells, so $p = 0$. In 2D, $p = 1$, and $\boldsymbol{\theta} = [0, 0, 1]$.

Table 5. Reference cell-cell mechanics parameters for a breast epithelial cell. Here, ν is the cell drag parameter in Equation 53, whose specific value is not needed in PhysiCell.

Parameter	Biophysical meaning	Reference value
n_{cca}	smoothness of cell-cell adhesive force	1
n_{ccr}	smoothness of cell-cell "repulsive" force	1
R_{cell}	equivalent cell radius	8.4 μm
R_{A}	maximum cell adhesion distance	1.25 $R_{\text{cell}} \approx 10.5$ μm
C_{ccr}	cell-cell "repulsive" repulsive force	10.0ν μm/min
C_{cca}	cell-cell adhesive force	0.4ν μm/min

Table 6. Reference cell-BM mechanics parameters for a breast epithelial cell. Here, ν is the cell drag parameter in Equation 53, whose specific value is not needed in PhysiCell.

Parameter	Biophysical meaning	Reference value
n_{cba}	smoothness of cell-BM adhesive force	1
n_{cbr}	smoothness of cell-BM "repulsive" force	1
R_{cell}	equivalent cell radius	8.4 μm
R_{A}	maximum cell adhesion distance	1.25 $R_{\text{cell}} \approx 10.5$ μm
C_{cbr}	cell-BM "repulsive" repulsive force	10.0ν μm/min
C_{cba}	cell-BM adhesive force	4ν μm/min

2 Numerical algorithm details

Here, we give additional (or expanded) detail on numerical algorithms used in the current release of PhysiCell.

2.1 Decoupling Velocity and Position Updates

Each cell velocity $\mathbf{v}_i$ depends upon the positions of its neighbors, and not their velocities. We write this as a system:

$$\dot{\mathbf{x}}_i = \mathbf{f}\left(\mathbf{x}_j\right)_{j\in\mathcal{N}(i)}, \qquad \text{for } 1 \le i \le n(t). \tag{69}$$

where $n(t)$ is the number of agents at time t. As in [35], rather than solving this system at once, we first compute $\mathbf{v}_i$ for $1 \le i \le n(t)$, and then we update $\mathbf{x}_i$ by $\dot{\mathbf{x}}_i = \mathbf{v}_i$. Because the $\{\mathbf{v}_i\}$ calculations do not change $\{\mathbf{x}_i\}$, the velocities can be computed in parallel, in any order. Likewise, the positions can be updated in parallel in any order.

2.2 ODE solutions

We solve the ordinary differential equations for cell volume using the forwards Euler method. We update the cell position using the second-order Adams-Bashforth method:

$$\mathbf{x}_i(t+\Delta t) = \mathbf{x}_i(t) + \frac{1}{2}\Delta t\Big(3\mathbf{v}_i(t) - \mathbf{v}_i(t-\Delta t)\Big). \tag{70}$$

This allows higher-order accuracy (and larger time steps) at approximately the same computational cost as the forwards Euler method, by storing $\mathbf{v}_i(t-\Delta t)$ in addition to $\mathbf{v}_i(t)$ for each agent.

2.3 Biotransport

Diffusion, secretion, uptake, and decay are solved using the 3-D solvers in BioFVM [18]. The first PhysiCell release uses BioFVM Version 1.1.5.

Table 7. Reference parameter values for breast epithelial cell motility.

Parameter	Biophysical meaning	Reference value
$T_{\text{persistence}}$	mean migration persistence time	15 min
s_{loc}	mean migration speed	1 μm/min

2.4 Pseudorandom number generator and probabilities

We use the built-in 64-bit Mersenne Twister pseudorandom number generator (PRNG) and uniform random distribution functions that are standard to any C++11 or later compiler, seeded to either a user-specified value or the system clock. We evaluate statements of the form "cell behavior $\mathcal{A}$ occurs in $[t, t + \Delta t]$ with probability p" as:

1. Select a random number $0 \leq r \leq 1$ from the uniform random function.
2. If $r \leq p$, then make the cell do behavior $\mathcal{A}$.

See the supplementary material of [35] for further discussion.

2.5 Choice of time step values

In [18], we found that $\Delta t_{\text{diffusion}} = 0.01$ min gives stable and accurate results (relative error 5% or less) for diffusion, decay, and secretion rates typical of cancer biology. In our testing (see Section 3), we find that $\Delta t_{\text{mechanics}} = 0.1$ min accurately and stably computes the cell mechanics (for tissue engineering and cancer biology problems), and $\Delta t_{\text{cell}} = 6$ min sufficiently resolves the ~ 1 hour time scales in cell cycling, death, and volume changes.

2.6 Interaction testing data structure (expanded detail)

Naïve implementations of cell-cell interactions result in a prohibitive $\mathcal{O}(n^2)$ computational cost scaling, where $n(t)$ is the number of agents. To prevent this, we designed a cell-cell interaction data structure (IDS) that estimates the set $\mathcal{N}(i)$ of neighbor cells for the ith agent. We divide the simulation domain into a set of voxels (boxes) $\{B_j\}$ of equal size. Each B_j tracks a list $\mathcal{L}_j$ of the agents it contains. To estimate $\mathcal{N}(i)$, we (1) determine which B_k contains the agent, and (2) we approximate

$$\mathcal{N}(i) \quad \approx \quad \bigcup \Big\{ \mathcal{L}_j \text{ such that } B_j \text{ shares an edge with } B_k \Big\} \cup \mathcal{L}_k. \tag{71}$$

That is, we estimate $\mathcal{N}(i)$ by concatenating the cell lists in B_k and its neighboring voxels. In our Cartesian mesh, this gives a maximum of 27 lists to concatenate. As an additional acceleration, we test whether the agent is near an edge of B_k, allowing us to eliminate some of the neighbor voxels from $\mathcal{N}(i)$.

2.7 Computational cost estimates (expanded detail)

In the main text, we discussed that most of the simulation steps have computational cost that scales linearly in the number of cells. The step that requires additional analysis (and relies upon PhysiCell's IDS) is the step where cell-cell mechanical interactions are used to set the cell velocities. Bounding this computational costs requires that we find a fixed upper bound on the number of cell-cell interactions, so that the computational cost is $\mathcal{O}(1)$ for single cells, and $\mathcal{O}(n^2)$ for all the cells.

We estimate an upper bound on the of cells in any voxel B_i by

$$N_{\max} \leq \frac{V_{\text{mech}}}{\min \{ V_i : \mathbf{x}_i \in B_i \}}, , \tag{72}$$

where V_{mech} is the fixed volume of the voxels in the interaction testing data structure. For cycling cells with "mature" volume V, we have $V_i \geq \frac{1}{2} V$. By default, dead cells are removed when $V_i \leq 20\ \mu\text{m}^3 (\approx \frac{1}{100} V)$. Since a typical $\mathcal{N}(i)$ is constructed from up to 27 such voxels, we have

$$N_{\max} \leq 27 \frac{V_{\text{mech}}}{\frac{1}{2} V} = 54 \frac{V_{\text{mech}}}{V} \tag{73}$$

for simulations dominated by live cells, and

$$N_{\max} \leq 27 \frac{V_{\text{mech}}}{\frac{1}{100} V} = 2700 \frac{V_{\text{mech}}}{V} \tag{74}$$

for simulations dominated by dead cells. Thus, the computational cost for a single cell's mechanical interactions is bounded by a fixed constant, and the total cost over all cell-cell mechanical interactions scales linearly in n. The slope of the cost-versus-n curve may be shallower for early, non-necrotic simulations, and it can be up to a factor of 100 steeper for necrosis-dominated simulations. In some cases, simulations may temporarily show a nonlinear relationship with n when transitioning from non-necrotic to necrotic.

3 Convergence and validation testing

We tested all the main components of PhysiCell to ensure convergence and validate the code.

3.1 Cell volume

To make sure that our "update volume" method works as we expected, solved Equations 1-8 using the reference parameter values in Table 1. In each of the tests below, we simulated a single cell's volume in a separate cell cycle phase (Ki-67 Advanced model–see Section 1.2.3) or death phase, and computed the convergence rate for the overall cell volume with respect to Δt. We solved with $\Delta t \in \{60, 6, 0.6, 0.06\}$ min. Since we do not have any analytical solution for computing the cell volume, we used the numerical solution for $\Delta t = 0.06$ min to approximate the error. If $V_{\Delta t}(t)$ is the numerical solution of $V(t)$ using Δt, then we approximate the relative error by

$$\mathrm{Err}(\Delta t, t) = \frac{|V_{\Delta t}(t) - V_{0.06}(t)|}{V_{0.06}(t)}. \tag{75}$$

We then estimate the order of convergence by computing the slope of the linear least squares fit of the points $\{\log \Delta t, \log \mathrm{Err}(\Delta t, t)\}$ at each time t. (Since $\mathrm{Err} = C\Delta t^n \implies \log \mathrm{Err} = \log C + n \log \Delta t$. See the brief discusion at http://www.mathcancer.org/blog/convergence_math/.)

In all cases, we obtained first-order convergence in time, consistent with our use of the first-order forwards Euler discretization.

3.1.1 Test 1: cell volume in the Ki67 advanced model (deterministic variant)

To compute the convergence rate in time for the cell volume at different phases of the cell cycle, we simulated 20 hours of `Advanced_KI67_model` (deterministic variant) cell cycle for a cell starting from K_1 phase. The length of K_1 phase was 13 hours, K_2 was 2.5 hours, and Q had a random length with mean duration 74.35 hours. The relative error values for $\Delta t \in \{60, 6, 0.6\}$ min for the cells in K_1 state are plotted in Figure 5.a and recorded in Table 8.

Since K_2 is 2.5 hours long, the $\Delta t = 60$ min simulation cannot capture the division accurately and then, as the result, the volume of the cell is very inaccurate around and after the time of cell division. So for the K_2 and Q phases, we just report the relative error for $\Delta t \in \{0.6, 6\}$ min as plotted in Figure 5.b and recorded in Table 9. In this example, we see first-order convergence in time at several solution times for the K_1, K_2, and Q phases. This shows good accuracy on both short and long time scales. Notice that for each fixed Δt for the K_2 and Q phases, the errors are largest for the first time points, when volume changes are fastest.

Table 8. Convergence in Δt for cell volume at K1 phase. For all times, we observe approximately first-order convergence.

	Err(Δt)			
Time (min)	$\Delta t = 60$ min	$\Delta t = 6$ min	$\Delta t = 0.6$ min	order
60	4.24e-02	1.58e-03	1.33e-04	1.25
300	4.03e-02	3.36e-03	3.01e-04	1.06
600	1.73e-02	1.73e-03	1.56e-04	1.02
780	8.88e-03	9.52e-04	8.82e-05	1.00
mean				1.02

Table 9. Convergence in Δt for cell volume at K2 and Q phases. For all times, we observe approximately first-order convergence.

	Err(Δt)		
Time (min)	$\Delta t = 0.6$ min	$\Delta t = 6$ min	order
840	7.72e-04	6.94e-05	1.05
900	6.23e-04	5.70e-05	1.04
960	4.99e-04	4.47e-05	1.05
1200	2.02e-04	1.61e-05	1.10
mean			1.03

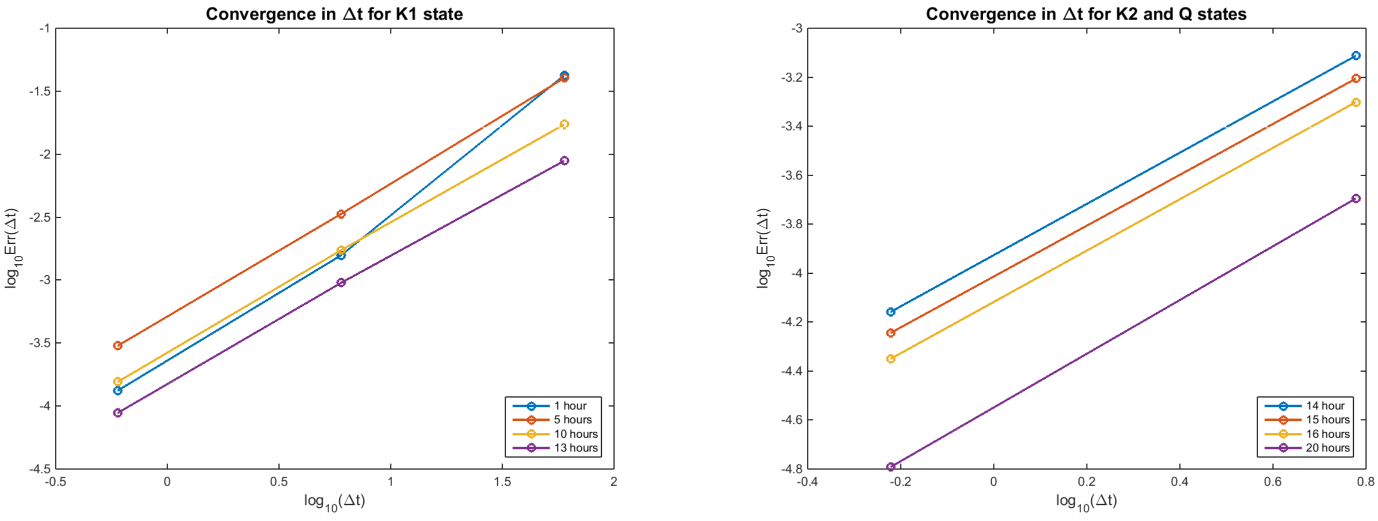

(a) (b)

Figure 5. Convergence in Δt for cell volume in different phases of KI67-advanced cell cycle model. Each curve gives the error across Δt at a different time. Notice that for each fixed Δt, the error improves in time as the solution approaches steady state. (a) Relative error for cell volume in K1 state for $\Delta t \in \{0.6, 6, 60\}$ min (compared to cell volume for $\Delta t = 0.06$ min as the reference value); see Table 8. (b) Relative error for cell volume in K1 state for $\Delta t \in \{0.6, 6\}$ min (compared to cell volume for $\Delta t = 0.06$ min as the reference value); see Table 9.

3.1.2 Test 2: cell volume in the apoptosis model

We next simulated 8 hours of cell apoptosis (Section 1.3.1.) using the reference parameter values. (See Section 1.3.1.) The relative error values for $\Delta t \in \{60, 6, 0.6\}$ min for the cell volume (at several times) are plotted in Figure 6 and recorded in Table 10. In this example, we see approximately first-order convergence in time when using all the Δt values; considering the two smaller Δt shows a first-order convergence more clearly. Notice that for each fixed Δt, the errors are largest for the first time points, when volume changes are fastest.

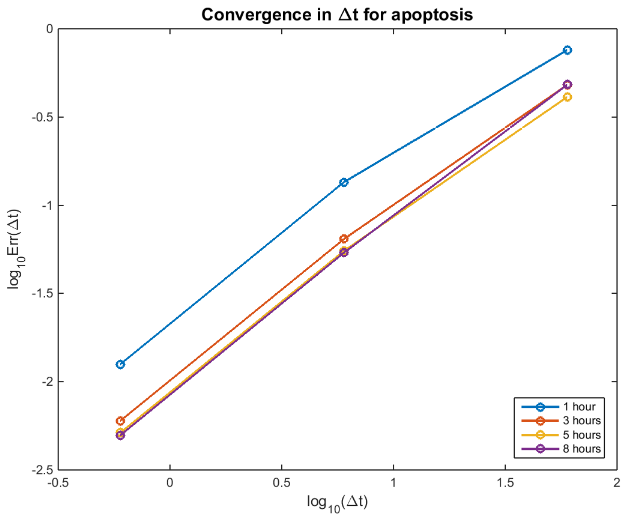

Figure 6. Convergence in Δt for cell volume at apoptotic phase. Relative error for cell volume in apoptotic phase for $\Delta t \in \{0.6, 6, 60\}$ min (compared to cell volume for $\Delta t = 0.06$ min as the reference value); see Table 10. Each curve gives the error across Δt at a different time. Notice that for each fixed resolution Δt, the errors are largest for the first time points, when volume changes are fastest.

Table 10. Convergence in Δt for cell volume at apoptotic phase. For all times, we observe approximately first-order convergence.

	Err(Δt)			
Time (min)	$\Delta t = 60$ min	$\Delta t = 6$ min	$\Delta t = 0.6$ min	order
60	7.59e-01	1.35e-01	1.26e-02	0.89
180	4.82e-01	6.43e-02	6.01e-03	0.95
300	4.11e-01	5.51e-02	5.14e-03	0.95
480	4.82e-01	5.38e-02	4.98e-03	0.99
mean				0.95

3.1.3 Test 3: cell volume in the necrosis model

As in the previous tests, we computed the convergence rate in time for the cell volume during necrosis. (See Section 1.3.2 for a description of the necrosis model and Section 1.3.2 for parameter values.) We simulated 20 hours of cell necrosis where cell goes through oncosis and lysis. The relative error values for $\Delta t \in \{60, 6, 0.6\}$ min for the cell volume are plotted in Figure 7 and recorded in Table 11. In this example, we see first-order convergence in time.

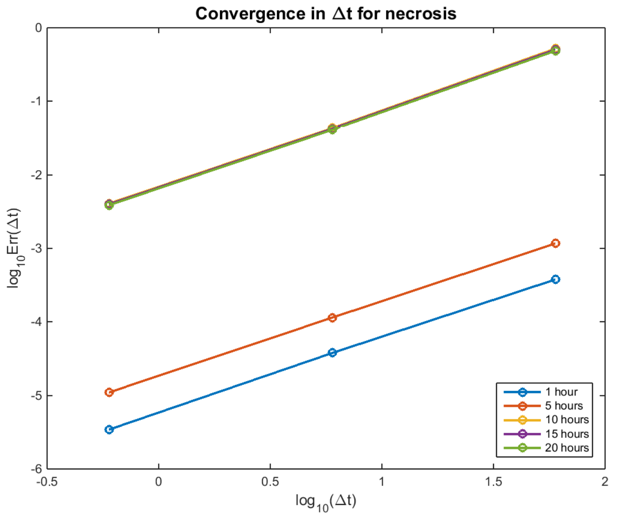

Figure 7. Convergence in Δt for cell volume at necrotic phase. Relative error for cell volume in necrotic phase for $\Delta t \in \{0.6, 6, 60\}$ min (compared to cell volume for $\Delta t = 0.06$ min as the reference value); see Table 11. Each curve gives the error across Δt at a different time.

Table 11. Convergence in Δt for cell volume at necrosis phase. For all times, we observe approximately first-order convergence.

	Err(Δt)			
Time (min)	$\Delta t = 60$ min	$\Delta t = 6$ min	$\Delta t = 0.6$ min	order
60	3.78e-04	3.78e-05	3.44e-06	1.02
300	1.17e-03	1.15e-04	1.10e-05	1.01
600	5.22e-01	4.33e-02	4.09e-03	1.05
900	5.06e-01	4.23e-02	3.99e-03	1.05
1200	4.87e-01	4.10e-02	3.87e-03	1.05
mean				1.04

3.1.4 Running the code for the volume tests

The code for these convergence tests is provided in `examples/convergence_test_volume.cpp`. This code needs the Δt as the input (in units of minutes) from the command line. To simulate the apoptosis process add "-a" as a

parameter when calling the executable file from a terminal; use "-n" for necrosis. Example (running in Windows): `test_volume.exe 0.6 -a`. The output will be written to vol_report.txt.

3.2 Cell mechanics and motion

We performed two tests to verify the accuracy and convergence of cell mechanics and motion in PhysiCell: the movement of two partially overlapping cells towards equilibrium, and the expansion (mechanical relaxation) of a compressed multicellular spheroid. As with the volume testing, we did not have an analytical solution for computing the errors. We used a high-resolution simulations ($\Delta t = 0.001$ min) to estimate the errors (which are defined for each problem below). In all the tests, we set all the cells to the Q phase in the Ki-67 Advanced model, with cell cycling, death, and volume changes disabled.

In all the tests, the cells had volume $V = 2494\,\mu\mathrm{m}^3$, $C_{\mathrm{ccr}} = 10\nu\ \mu\mathrm{m/min}$, $C_{\mathrm{cca}} = 0.170577155519015\nu\ \mu\mathrm{m/min}$, and $R_{\mathrm{A}} = 1.5\,R_{\mathrm{cell}}$.

3.2.1 Test 1: pairwise cell mechanics

We first simulated the mechanical interactions between two partly overlapping cells until they reach an equilibrium separation. We placed two cells close to each other with about 50% overlap and let the mechanical forces (repulsion and adhesion) move the cells towards an equilibrium separation. We simulated with $\Delta_{\mathrm{mechanics}} = \Delta tt \in \{1, 0.1, 0.01, 0.001\}$ min, and recorded $d_{\Delta t}(t)$: the distance between cell centers at time t, simulated with step size $\Delta t_{\mathrm{mechanics}} = \Delta t$. (Note that because Adams-Bashforth requires current and previous velocities at $t = 0$, we simulated up to $t = \Delta t$ with the forward Euler method using a temporary time step size of $0.1\,\Delta t$.)

For each Δt and recorded time t, we approximated the relative error by

$$\mathrm{Err}(\Delta t, t) = \frac{|d_{\Delta t}(t) - d_{0.001}(t)|}{d_{0.001}(t)}. \tag{76}$$

Table 12 shows the relative errors at 1, 10, 20, 30, 45, and 60 minutes, computed for the different Δt values. At each time, we observed a convergence rate between first- and second-order, and the solutions were stable even for large Δt. The distances quickly converged towards their known equilibrium spacing, where $||\mathbf{F}_{\mathrm{cca}}|| = ||\mathbf{F}_{\mathrm{ccr}}||$. For $\Delta t = 0.1$ min, the relative errors were below 10^{-6} for all times beyond 45 minutes. For $\Delta t = 0.01$ min, the relative error rapidly fell below 10^{-6} for all recorded times beyond 20 minutes. This complicated computation of the convergence rates, and so we restricted convergence rate calculations to errors exceeding 10^{-6}. At 60 minutes and beyond, only the $\Delta t = 1$ min solution had relative errors exceeding 10^{-6}.

Table 12. Convergence in Δt for two cells placed with 50% overlap. At each time, we observe a convergence rate between first- and second-order and the distances converge quickly to the expected value even for large Δt.
* Error below double precision.
** As one of the errors is zero, one of the orders is undefined. So the total order is just computed using Err(1 min) and Err(0.1 min).

Time	Err(Δt), where Δt is:			
(min)	1 min	0.1 min	0.01 min	order
5	9.96e-02	2.56e-03	2.12e-04	1.34
15	1.99e-02	3.40e-04	2.70e-05	1.39
30	4.09e-03	5.81e-05	5.28e-06	1.43
45	1.04e-03	1.58e-05	0*	1.82**
59	2.99e-04	5.25e-06	0*	1.76**
mean				1.55

3.2.2 Test 2: compressed spheroid example

As a much more challenging test of the mechanics code, we simulated the mechanical relaxatio of a compressed cluster of 44,661 cells, arranged in a sphere with about 80% overlap with their neighbors, using $\Delta t_{\mathrm{mechanics}} = \Delta t \in \{1, 0.1, 0.01, 0.001\}$ min. For any Δt and time t, we measured the volume $V_{\Delta t}(t)$ of the expanding multicellular spheroid and converted it to an equivalent radius $R_{\Delta t}(t)$ via

$$V_{\Delta t}(t) = \frac{4}{3}\pi R^3_{\Delta t}(t). \tag{77}$$

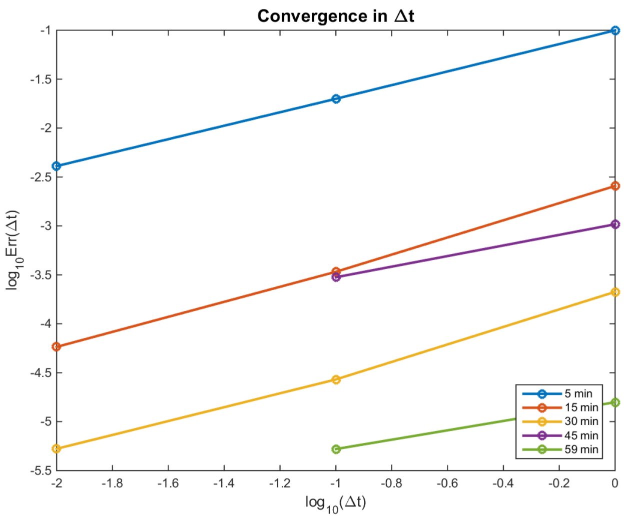

Figure 8. Convergence in Δt for the distance of two cells placed with 50% overlap. Relative error for the distance of two cells placed with 50% overlap computed for $\Delta t \in \{0.01, 0.1, 1\}$ min (compared to radius for $\Delta t = 0.001$ min as the reference value); see Table 12. Each curve gives the error across Δt at a different time.

We estimated the relative error by

$$\text{Err}(\Delta t, t) = \frac{|R_{\Delta t}(t) - R_{0.001}(t)|}{R_{0.001}(t)}. \tag{78}$$

Table 13 shows the relative error of the estimated radius of the the sphere. We observed approximately second-order convergence for this example, particularly for later times when the tissue was closer to mechanical homeostasis–a situation closer to the norm for a slowly-evolving tissue.

Table 13. Convergence in Δt for a spheroid of 45k cells placed with 80% overlap.

Time	Err(Δt), where Δt is:			
(min)	1 min	0.1 min	0.01 min	order
5	0.4418	2.27e-2	1.75e-4	1.70
10	0.3100	1.55e-2	9.11e-5	1.77
20	0.2040	9.08-3	5.49e-5	1.78
30	0.1609	6.45e-3	2.43e-5	1.91
45	0.1282	4.46e-3	3.57e-6	2.27
60	0.1090	3.75e-3	7.12e-6	2.09
mean				1.92

3.2.3 Running the code for the mechanics tests

The code for the two-cells convergence test is provided in `examples/convergence_test_mechanics1.cpp` and for the spheroid one is in `convergence_test_mechanics2.cpp`. These codes receive the Δt as the input from the command line.

3.3 Cell phenotype changes

We tested the cell phenotype switching by comparing to the known population-scale behavior. Following the analyses in [35], if $K_1(t)$, $K_2(t)$, $Q(t)$, and $A(t)$ are the number of cells in the K_1, K_2, Q, and A (apoptotic)

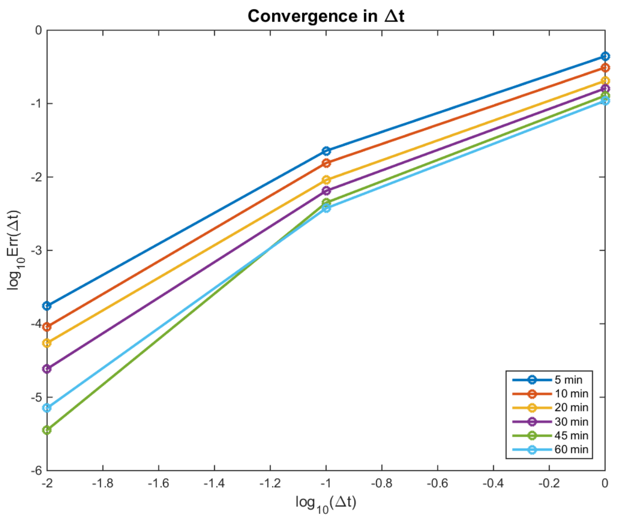

Figure 9. Convergence in Δt for the radius of a spheroid of 45k cells placed with 80% overlap. Relative error for the radius of a spheroid of 45k cells placed with 80% overlap computed for $\Delta t \in \{0.01, 0.1, 1\}$ min (compared to radius for $\Delta t = 0.001$ min as the reference value); see Table 13. Each curve gives the error across Δt at a different time.

phases at time t, then for sufficiently large populations of cells, the Ki67 Advanced model can be coarse-grained as

$$\frac{d}{dt}K_1 = \frac{1}{T_\mathrm{Q}}Q - \left(\frac{1}{T_1} + r_{1\mathrm{A}}\right)K_1 \tag{79}$$

$$\frac{d}{dt}K_2 = \frac{2}{T_1}K_1 - \left(\frac{1}{T_2} + r_{2\mathrm{A}}\right)K_2 \tag{80}$$

$$\frac{d}{dt}Q = \frac{1}{T_2}K_2 - \left(\frac{1}{T_\mathrm{Q}} + r_{\mathrm{QA}}\right)Q \tag{81}$$

$$\frac{d}{dt}A = r_{1\mathrm{A}}K_1 + r_{2\mathrm{A}}K_2 + r_{\mathrm{QA}}Q - \frac{1}{T_\mathrm{A}}A. \tag{82}$$

Because this model is stochastic, individual runs will not match this behavior except for long times where there are many cells. However, (1) for any single simulation run, the individual growth curves of K_1, K_2, Q, and A should tend to the same growth rates as the analytical solution of Equations 80-82, and (2) as we run many simulations with the same parameter values, the mean of the solutions should converge to the analytical solution of the ODE model above.

3.3.1 Analytical solutions

If we let $\mathbf{p} = [K_1, K_2, Q, A]^T$ (^{T} denotes the transpose), then the system above can be written $\dot{\mathbf{p}} = M\mathbf{p}$. If M has eigenvalues $\{\lambda_i\}_{i=1}^4$ and corresponding eigenvectors $\{\mathbf{v}_i\}_{i=1}^4$, then the general solution can be written as

$$\mathbf{p}(t) = \sum_{i=1}^{4} c_i e^{\lambda_i t}\mathbf{v}_i. \tag{83}$$

Suppose that the initial cell distribution is given as $\mathbf{p}(0)$. Then we can determine the coefficients by solving

$$\mathbf{p}(0) = [\mathbf{v}_1|\mathbf{v}_2|\mathbf{v}_3|\mathbf{v}_4]\,\mathbf{c}, \tag{84}$$

where $\mathbf{c} = [c_1, c_2, c_3, c_4]^T$. Moreover, $\lambda_1, \lambda_2, \lambda_3 < 0$ and $\lambda_4 > 0$, then for long times,

$$\mathbf{p}(t) \approx c_4 e^{\lambda_4 t}\mathbf{v}_4. \tag{85}$$

Thus, if $||\mathbf{p}||_1 = \sum_{i=1}^n |p_i|$, and if we define a series of indices $\mathrm{PI}_i = p_i / ||\mathbf{p}||_1$ for each i, we have (for long solution times)

$$\mathrm{PI}_i = v_i / ||\mathbf{v}||_1\,. \tag{86}$$

We provide further discussion and a matlab script to numerically obtain the solution at:

http://MathCancer.org/blog/coarse-graining-discrete-models/.

3.3.2 Validation testing

We tested the advanced Ki-67 model (See Section 1.2.3), with $T_1 = 13$ hours, $T_2 = 2.5$ hours, $T_A = 8.6$ hours, $r_{1A} = r_{2A} = r_{QA} = 1.05 \times 10^{-3}$ hour^{-1}, and $T_Q = 74.35$ hours. We initialized the simulations with 1000 cells in the Q phase, and simulated 6 days of growth. We disabled the mechanics, motion, microenvironment, and volume codes to focus the testing on the phenotype switching.

For each $\Delta t \in \{60, \ldots, 0.01\}$ min, we ran $n = 100$ simulations, and we computed the mean solutions $\langle K_1(t)\rangle$, $\langle K_2(t)\rangle$, $\langle Q(t)\rangle$, and $\langle A(t)\rangle$. If

$$\langle N(t)\rangle = \langle K_1(t)\rangle + \langle K_2(t)\rangle + \langle Q(t)\rangle + \langle A(t)\rangle \tag{87}$$

is the mean total number of cells at time t, then we can define and compute the indices

$$\langle \mathrm{KI}_1(t)\rangle = \frac{\langle K_1(t)\rangle}{\langle N(t)\rangle}, \qquad \langle \mathrm{KI}_2(t)\rangle = \frac{\langle K_2(t)\rangle}{\langle N(t)\rangle}, \qquad \text{and} \qquad \langle \mathrm{AI}(t)\rangle = \frac{\langle A(t)\rangle}{\langle N(t)\rangle}. \tag{88}$$

Hereafter, we drop the $\langle\cdot\rangle$ notation, but continue to refer to the mean across the n simulations, at any fixed resolution Δt. In Figure 10, we plot the solutions K_1, K_2, Q, and A for each of the values of Δt. There was excellent agreement with the theoretical behavior (the blue curves) in each of the populations for all Δt values. In Figure 11, we plotted KI_1 , KI_2, and AI for each of the values of Δt. Again, we saw excellent agreement with the theoretical curves (blue curves) for all Δt values.

3.3.3 Running the phenotype testing code

The code for this convergence test is provided in `examples/PhysiCell_test_cell_cycle.cpp`. This code needs the Δt as the input (in units of minutes) from the command line. Example (running in Windows): `test_cycle.exe 0.6`. The output will be written to `output` folder for each time step.

4 Parameter values for the main examples

For both main examples, we used the Ki-67 advanced model (legacy variant), with $T_1 = 13$ hours, $T_2 = 2.5$ hours, $\langle T\rangle_Q = 74.35$ hours, and $T_A = 8.6$ hours.

The volume parameters were set to the reference values in Section 1.1.1. The mechanics parameters were set to the reference values in Section 1.4.6, with the following exceptions: $R_A = 1.5 R_{\mathrm{cell}}$ (which we used to test more challenging cell deformations and cell-cell interactions), $C_{\mathrm{cca}} = 0.1706$, and $C_{\mathrm{cba}} = 1.7058$ (for DCIS example).

4.1 Main example 1: hanging drop spheroids

We used a 2000 μm $\times$ 2000 μm $\times$ 2000 μm computational domain with 20μm resolution for this experiment. We initialized the spheroid with 2347 cells arranged using sphere packing technique (initial spheroid radius was 150 μm). We set $\mathrm{pO}_2 = 38$ mmHg all over the domain and all cells start from Q phase. The tumor in simulation with deterministic necrosis model reaches the boundaries of the computational domain in about 18 days; for the stochastic necrosis model this duration is about 19 days.

4.2 Main example 2: ductal carcinoma in situ (DCIS)

For this example, the duct radius was $R = 158.75\,\mu$m, and we set $\mathrm{pO}_2 = 7.1930$ mmHg outside the duct. We used a 1200 μm $\times$ 400 μm $\times$ 400 μm computational domain with 20μm resolution for this experiment and initialized the simulation with 1153 cells arranged as a semi-sphere at the cap of the duct. All the cells start from Q phase.

4.3 Running the code for the main examples

The codes for DCIS and HDS examples are provided in `examples/PhysiCell_test_DCIS.cpp` and `examples/PhysiCell_test_HDS.cpp` respectively.

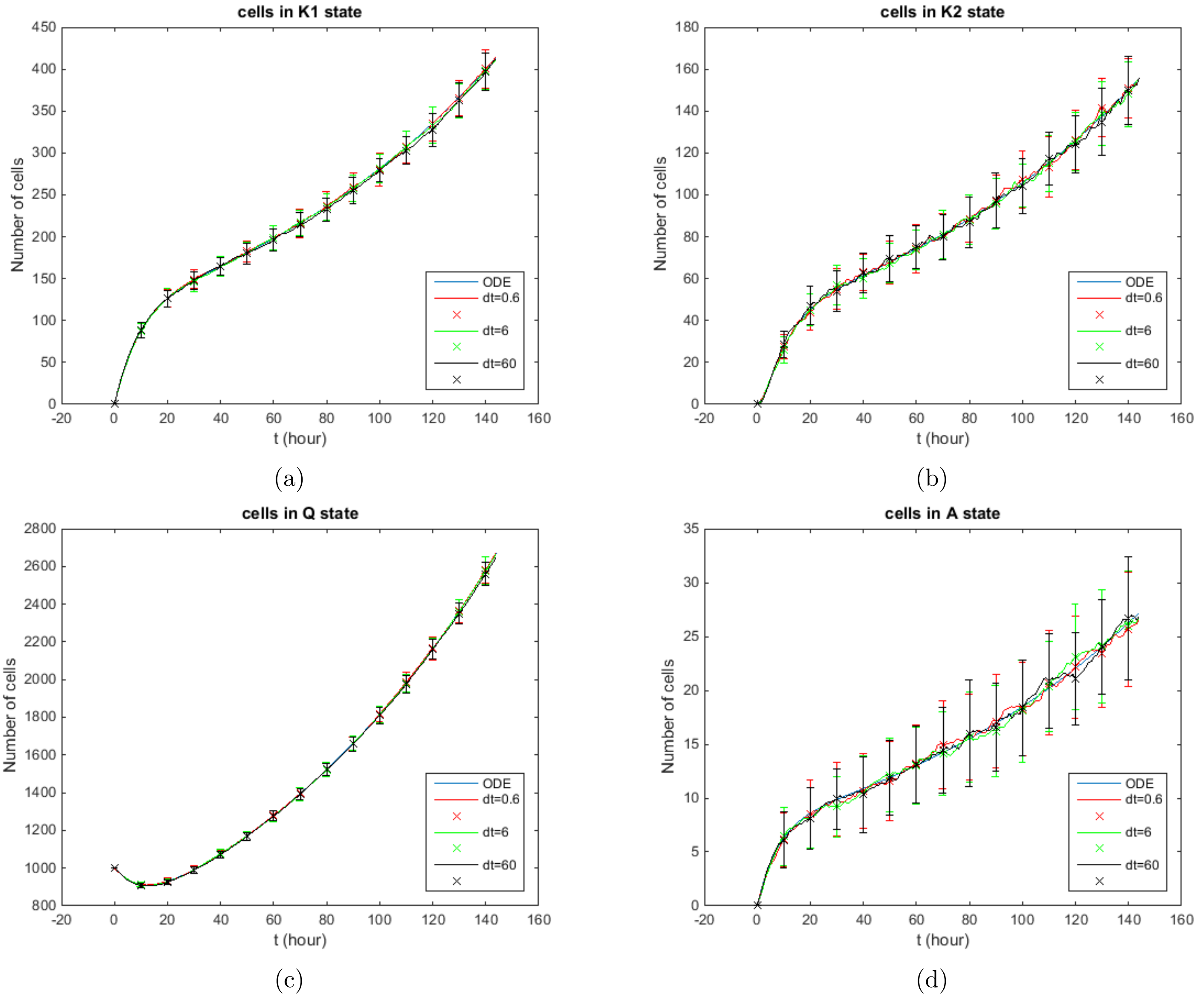

Figure 10. Number of cells in different phases of KI-67 advanced cell cycle model for $\Delta t \in \{0.6, 6, 60\}$ min. We simulated 6 days of of growth starting from 1000 cells and kept the track of the cells in different phases for each Δt. (a) cells in K1 phase, (b) cells in K2 phase, (c) cells in Q phase, and (d) cells in A phase.

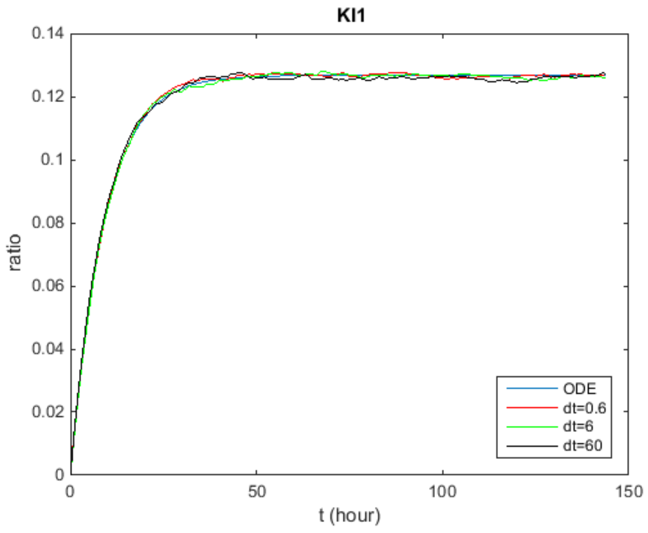

(a)

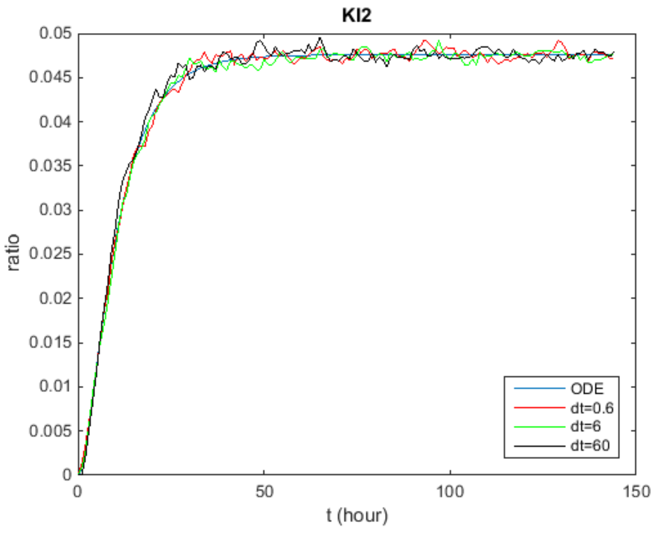

(b)

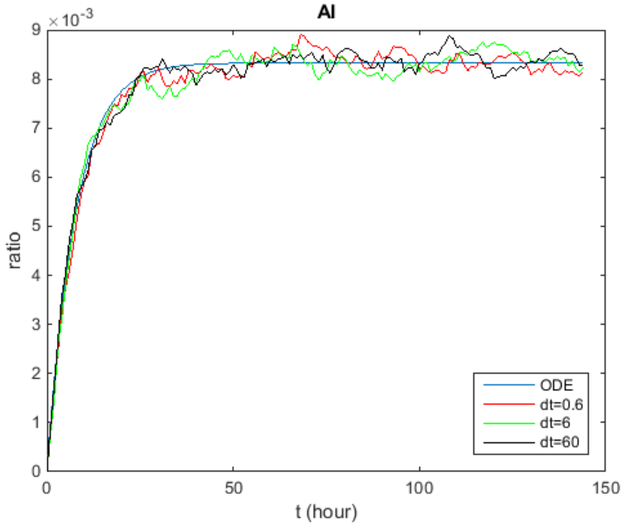

(c)

Figure 11. Ratio of cells in different phases of KI-67 advanced cell cycle model for $\Delta t \in \{0.6, 6, 60\}$ min. We simulated 6 days of of growth starting from 1000 cells and kept the track of the cells in different phases for each Δt. (a) ratio of cells in K1 phase, (b) ratio of cells in K2 phase, and (c) ratio of cells in A phase.

5 Comparison of PhysiCell with other major 3-D multicellular simulators

In Table 14, we compare the features of PhysiCell and several other major 3-D multicellular simulators from multiple points of view: built-in standard models, workstation performance, licensing, extensibility, software complexity (in terms of ease of installation and dependencies), and cross-platform compatibility.

5.1 Notes

In this comparison, we only include cell cycle, apoptosis, and other functionality that is included as standard in all downloads, rather than in online wikis or code samples. "Advanced" cell cycle models are those that go beyond a simple timer or division criterion, to explicitly model volume changes as well, or to include multiple built-in cell cycle representations (e.g., live/dead cells, flow cytometry, Ki67 pathology). "Minimal dependencies and development environment" denotes codes that require only 1-2 external dependencies and libraries beyond standard C++ compiler and makefile software. "Cross-platform compatible" means that the code can generate native binaries using a C++ 2011-compliant compiler in at least Linux, Windows, and OSX, rather than run by virtual machine. Of all the licenses, only BSD-licensed codes and GPL-licensed codes were found to be free for commercial use, although GPL-based commercial software still require full publication of derivative source code.

Table 14. Software feature comparison matrix: Comparison of features of leading 3-D multicellular simulation packages, using out-of-the-box settings and functionality.
°Division triggered by simple timer or volume parameter. ★Models duration and volume change, and/or multiple built-in models. ✠"bundling" via git command. *Simple installation for Ubuntu only. All other platforms are complex. ◊subject to the terms of GPL.

**Feature**	**Computational Framework**					
	**Chaste (3.64)** [41]	**Morpheus (2.0 beta)** [46]	**CompuCell3D (3.7.5)** [48]	**Biocellion (1.1)** [28]	**CellSys 2010 (5.0)** [24]	**PhysiCell (1.2.1)**
Custom functions	Y	Y	Y	N	N	Y
Custom function for each cell type	Y	Y	Y	N	N	Y
Custom function for individual cells	Y	N	N	N	N	Y
Integrated subcellular signaling models	Y	Y	Y	N	N	N
Integrated fast multi-substrate diffusion	N	N	N	N	N	Y
Integrated general PDE solvers	Y	Y	Y	Y	N	N
Integrated general ODE solvers	Y	Y	Y	N	N	N
Built-in cell volume model	N	Y	Y	N	Y	Y
Built-in cell fluid/solid volume model	N	N	N	N	N	Y
Built-in cell morphology models	Y	Y	Y	N	N	N
Built-in cell cycle model: <u>N</u>one/<u>B</u>asic°/<u>A</u>dvanced★	A	B	B	N	A	A
Built-in apoptosis model	Y	Y	Y	N	Y	Y
Built-in necrosis model	N	N	N	N	N	Y
Anonymous downloads	N	Y	Y	N	Y	Y
All dependencies bundled in download	N	Y✠	Y	N	n.a.	Y
Minimal dependencies and development environment	N	N	Y	Y	n.a.	Y
Simple source installation	Y*	Y	Y	Y	n.a.	Y
cross-platform compatible	N	Y	Y	N	Y	Y
General executable "client" with XML/text input	Y	Y	Y	N	N	N
Windows native binary available	N	Y	Y	N	Y	N
OSX native binary available	N	Y	Y	N	N	N
Linux native binary available	Y	Y	Y	N	N	N
Runs on desktop workstation or single HPC node	Y	Y	Y	Y	Y	Y
GPU acceleration	N	N	Y	N	N	N
Runs single simulation on cluster (MPI)	N	N	N	Y	N	N
Simulate 10^5+ cells on workstations	Y	N	N	Y	Y	Y
Simulate 10^6+ cells on workstations	Y	N	N	Y	Y	Y
Simulate 10^9+ cells on clusters	N	N	N	Y	N	N
Free for commerical use	Y	Y	Y◊	N	N	Y
Free for academic use	Y	Y	Y	Y	Y	Y
Source code available	Y	Y	Y	N	N	Y
Clear permission to publish application source	Y	Y	Y	N	n.a.	Y
OSI-compliant open source license	Y	Y	Y	N	N	Y
GPL2-compliant license	Y	Y	Y	N	N	Y

Bibliography

[1] K. R. Albe, M. H. Butler, and B. E. Wright. Cellular concentrations of enzymes and their substrates. *J. Theor. Biol.*, 143(2):163–95, 1990. doi: 10.1016/S0022-5193(05)80266-8. URL http://dx.doi.org/10.1016/S0022-5193(05)80266-8.

[2] M. Aoyagi, M. Yamamoto, H. Wakimoto, H. Azuma, K. Hirakawa, and K. Yamamoto. Immunohistochemical detection of Ki-67 in replicative smooth muscle cells of rabbit carotid arteries after balloon denudation. *Stroke*, 26(12):2328–2332, 1995. doi: 10.1161/01.STR.26.12.2328. URL http://dx.doi.org/10.1161/01.STR.26.12.2328.

[3] M. A. Banks, D. W. Porter, W. H. Pailes, D. Schwegler-Berry, W. G. Martin, and V. Castranova. Taurine content of isolated rat alveolar type I cells. *Comp. Biochem. Physiol. B*, 100(4):795–9, 1991. doi: 10.1016/0305-0491(91)90292-L. URL http://dx.doi.org/10.1016/0305-0491(91)90292-L.

[4] L. F. Barros, T. Hermosilla, and J. Castro. Necrotic volume increase and the early physiology of necrosis. *Comp. Biochem. Physiol. A. Mol. Integr. Physiol.*, 130(3):401–9, 2001. doi: 10.1016/S1095-6433(01)00438-X. URL http://dx.doi.org/10.1016/S1095-6433(01)00438-X.

[5] S. Bruno and Z. Darzynkiewicz. Cell cycle dependent expression and stability of the nuclear protein detected by Ki-67 antibody in HL-60 cells. *Cell Proliferation*, 25(1):31–40, 1992. ISSN 1365-2184. doi: 10.1111/j.1365-2184.1992.tb01435.x. URL http://dx.doi.org/10.1111/j.1365-2184.1992.tb01435.x.

[6] H. M. Byrne and D. Drasdo. Individual-based and continuum models of growing cell populations: A comparison. *J. Math. Biol.*, 58(4–5):657–87, 2009. doi: 10.1007/s00285-008-0212-0. URL http://dx.doi.org/10.1007/s00285-008-0212-0.

[7] S. Byun, V. C. Hecht, and S. R. Manalis. Characterizing cellular biophysical responses to stress by relating density, deformability, and size. *Biophysical J.*, 109(8):1565–73, 2015. doi: 10.1016/j.bpj.2015.08.038. URL http://dx.doi.org/10.1016/j.bpj.2015.08.038.

[8] G. M. Cooper. The eukaryotic cell cycle. In *The Cell: A Molecular Approach*. 2nd edition, 2000. URL http://www.ncbi.nlm.nih.gov/books/NBK9876/.

[9] G. D'Antonio, P. Macklin, and L. Preziosi. An agent-based model for elasto-plastic mechanical interactions between cells, basement membrane and extracellular matrix. *Math. Biosci. Eng.*, 10(1):75–101, 2013. doi: 10.3934/mbe.2013.10.75. URL http://dx.doi.org/10.3934/mbe.2013.10.75.

[10] S. P. Desai, S. N. Bhatia, M. Toner, and D. Irimia. Mitochondrial localization and the persistent migration of epithelial cancer cells. *Biophysical journal*, 104(9):2077–2088, May 2013. ISSN 1542-0086. doi: 10.1016/j.bpj.2013.03.025. URL http://dx.doi.org/10.1016/j.bpj.2013.03.025.

[11] H. P. Dinkel, A. M. Gassel, and A. Tschammler. Is the appearance of microcalcifications on mammography useful in predicting histological grade of malignancy in ductal cancer in situ? *The British Journal of Radiology*, 73(873):938–44, 2000. doi: 10.1259/bjr.73.873.11064645. URL http://dx.doi.org/10.1259/bjr.73.873.11064645.

[12] D. Drasdo, R. Kree, and J. S. McCaskill. Monte Carlo approach to tissue-cell populations. *Phys. Rev. E*, 52:6635–6657, Dec 1995. doi: 10.1103/PhysRevE.52.6635. URL http://link.aps.org/doi/10.1103/PhysRevE.52.6635.

[13] M. E. Edgerton, Y.-L. Chuang, P. Macklin, W. Yang, E. L. Bearer, and V. Cristini. A novel, patient-specific mathematical pathology approach for assessment of surgical volume: Application to ductal carcinoma in situ of the breast. *Anal. Cell. Pathol.*, 34(5):247–63, 2011. doi: 10.3233/ACP-2011-0019. URL http://dx.doi.org/10.3233/ACP-2011-0019.

[14] Z. El-Schich, A. Mlder, H. Tassidis, P. Hrknen, M. F. Miniotis, and A. G. Wingren. Induction of morphological changes in death-induced cancer cells monitored by holographic microscopy. *Journal of Structural Biology*, 189(3):207 – 212, 2015. ISSN 1047-8477. doi: http://dx.doi.org/10.1016/j.jsb.2015.01.010. URL http://www.sciencedirect.com/science/article/pii/S104784771500026X.

[15] H. B. Frieboes, J. S. Lowengrub, S. M. Wise, X. Zheng, P. Macklin, E. L. Bearer, and V. Cristini. Computer simulation of glioma growth and morphology. *NeuroImage*, 37(S1):S59–S70, 2007. doi: 10.1016/j.neuroimage.2007.03.008. URL http://dx.doi.org/10.1016/j.neuroimage.2007.03.008.

[16] J. Galle, M. Loeffler, and D. Drasdo. Modeling the effect of deregulated proliferation and apoptosis on the growth dynamics of epithelial cell populations in vitro. *Biophys. J.*, 88(1):62–75, 2005. doi: 10.1529/biophysj.104.041459. URL http://dx.doi.org/10.1529/biophysj.104.041459.

[17] R. A. Gatenby, K. Smallbone, P. K. Maini, F. Rose, J. Averill, R. B. Nagle, L. Worrall, and R. J. Gillies. Cellular adaptations to hypoxia and acidosis during somatic evolution of breast cancer. *Br. J. Cancer*, 97(5): 646–53, 2007. doi: 10.1038/sj.bjc.6603922. URL http://dx.doi.org/10.1038/sj.bjc.6603922.

[18] A. Ghaffarizadeh, S. H. Friedman, and P. Macklin. BioFVM: an efficient, parallelized diffusive transport solver for 3-D biological simulations. *Bioinformatics*, 32(8):1256–1258, 2016. doi: 10.1093/bioinformatics/btv730. URL http://dx.doi.org/10.1093/bioinformatics/btv730.

[19] A. Ghaffarizadeh, R. Heiland, S. H. Friedman, S. M. Mumenthaler, and P. Macklin. PhysiCell: an open source physics-based cell simulator for 3-d multicellular systems. *PLoS Comput. Biol.*, 2018 (accepted). URL https://doi.org/10.1101/088773.

[20] D. J. Grignon. Unusual subtypes of prostate cancer. *Mod. Pathol.*, 17(3):316–27, 2004. doi: 10.1038/modpathol.3800052. URL http://dx.doi.org/10.1038/modpathol.3800052.

[21] J. Guck, S. Schinkinger, B. Lincoln, F. Wottawah, S. Ebert, M. Romeyke, D. Lenz, H. M. Erickson, R. Ananthakrishnan, D. Mitchell, J. Ks, S. Ulvick, and C. Bilby. Optical deformability as an inherent cell marker for testing malignant transformation and metastatic competence. *Biophysical Journal*, 88(5): 3689 – 3698, 2005. ISSN 0006-3495. doi: 10.1529/biophysj.104.045476. URL http://dx.doi.org/10.1529/biophysj.104.045476.

[22] C. C. Guo and J. I. Epstein. Intraductal carcinoma of the prostate on needle biopsy: histologic features and clinical significance, 2006. URL http://dx.doi.org/10.1038/modpathol.3800702.

[23] A. T. Hahn, J. T. Jones, and T. Meyer. Quantitative analysis of cell cycle phase durations and PC12 differentiation using fluorescent biosensors. *Cell Cycle*, 8(7):1044–1052, 2009. doi: 10.4161/cc.8.7.8042. URL http://dx.doi.org/10.4161/cc.8.7.8042. PMID: 19270522.

[24] S. Hoehme and D. Drasdo. A cell-based simulation software for multi-cellular systems. *Bioinformatics*, 26(20):2641–2642, 2010. doi: 10.1093/bioinformatics/btq437. URL http://dx.doi.org/10.1093/bioinformatics/btq437.

[25] J. Howard. *Mechanics of motor proteins and the cytoskeleton*. Sinauer Associates, Sunderland (Mass.), 2001. ISBN 978-0-87893-333-4. URL http://opac.inria.fr/record=b1130788.

[26] S. Huether and K. McCance. Altered cellular and tissue biology. In *Understanding Pathophysiology*, chapter 3. Mosby, St. Louis, MO USA, 5 edition, 2011. ISBN 978-0323078917.

[27] A. Z. Hyun and P. Macklin. Improved patient-specific calibration for agent-based cancer modeling. *J. Theor. Biol.*, 317:422–4, 2013. doi: 10.1016/j.jtbi.2012.10.017. URL http://dx.doi.org/10.1016/j.jtbi.2012.10.017.

[28] S. Kang, S. Kahan, J. McDermott, N. Flann, and I. Shmulevich. Biocellion: accelerating computer simulation of multicellular biological system models. *Bioinformatics*, 30(21):3101–3108, 2014. doi: 10.1093/bioinformatics/btu498. URL http://dx.doi.org/10.1093/bioinformatics/btu498.

[29] A. Khmaladze, R. L. Matz, T. Epstein, J. Jasensky, M. M. B. Holl, and Z. Chen. Cell volume changes during apoptosis monitored in real time using digital holographic microscopy. *Journal of Structural Biology*, 178(3):270 – 278, 2012. ISSN 1047-8477. doi: http://dx.doi.org/10.1016/j.jsb.2012.03.008. URL http://www.sciencedirect.com/science/article/pii/S104784771200086X.

[30] R. F. Krause and K. C. Beamer. Lipid content and phospholipid metabolism of subcellular fractions from testes of control and retinol-deficient rats. *The Journal of Nutrition*, 104(5):629–637, 1974. URL http://jn.nutrition.org/content/104/5/629.short.

[31] V. Kumar, A. K. Abbas, J. C. Aster, and N. Fausto. Cellular responses to stress and toxic insults: Adaptation, injury, and death. In *Pathologic Basis of Disease*, chapter 1. Saunders Elsevier, Philadelphia, PA USA, 8 edition, 2009. ISBN 978-1416031215.

[32] S. Larsson, T. Ryden, U. Holst, S. Oredsson, and M. Johansson. Estimating the variation in S phase duration from flow cytometric histograms. *Mathematical Biosciences*, 213(1):40–9, 2008. doi: 10.1016/j.mbs.2008.01.009. URL http://dx.doi.org/10.1016/j.mbs.2008.01.009.

[33] W. Lin and G. Arthur. Phospholipids are synthesized in the G2/M phase of the cell cycle. *The International Journal of Biochemistry & Cell Biology*, 39(3):597–605, 2007. ISSN 1357-2725. doi: 10.1016/j.biocel.2006.10.011. URL http://dx.doi.org/10.1016/j.biocel.2006.10.011.

[34] P. Macklin, S. R. McDougall, A. R. A. Anderson, M. A. J. Chaplain, V. Cristini, and J. S. Lowengrub. Multiscale modelling and nonlinear simulation of vascular tumour growth. *J. Math. Biol.*, 58(4-5):765–798, 2009. doi: 10.1007/s00285-008-0216-9. URL http://dx.doi.org/10.1007/s00285-008-0216-9.

[35] P. Macklin, M. E. Edgerton, A. M. Thompson, and V. Cristini. Patient-calibrated agent-based modelling of ductal carcinoma in situ (DCIS): From microscopic measurements to macroscopic predictions of clinical progression. *J. Theor. Biol.*, 301:122–40, 2012. doi: 10.1016/j.jtbi.2012.02.002. URL http://dx.doi.org/10.1016/j.jtbi.2012.02.002.

[36] P. Macklin, S. Mumenthaler, and J. Lowengrub. Modeling multiscale necrotic and calcified tissue biomechanics in cancer patients: application to ductal carcinoma in situ (DCIS). In A. Gefen, editor, *Multiscale Computer Modeling in Biomechanics and Biomedical Engineering*, chapter 13, pages 349–80. Springer, Berlin, Germany, 2013. ISBN 978-3-642-36482-2. doi: 10.1007/8415_2012_150. URL http://dx.doi.org/10.1007/8415_2012_150. (invited author: P. Macklin).

[37] S. D. Manoir, P. Guillaud, E. Camus, D. Seigneurin, and G. Brugal. Ki-67 labeling in postmitotic cells defines different Ki-67 pathways within the 2c compartment. *Cytometry*, 12(5):455–463, 1991. ISSN 1097-0320. doi: 10.1002/cyto.990120511. URL http://dx.doi.org/10.1002/cyto.990120511.

[38] E. Mendoz and C. Lim. Collective Migration Behaviors of Human Breast Cancer Cells in 2D. *Cellular and Molecular Bioengineering*, 4(3):411–426, Sept. 2011. ISSN 1865-5025. doi: 10.1007/s12195-011-0193-8. URL http://dx.doi.org/10.1007/s12195-011-0193-8.

[39] R. Milo and R. Phillips. What is the density of cells? In *Cell Biology by the Numbers*. Garland Science, 2015. ISBN 978-0815345374. URL http://book.bionumbers.org/what-is-the-density-of-cells/.

[40] R. Milo, P. Jorgensen, G. Weber, and M. Springer. BioNumbers–the database of key numbers in molecular and cell biology. 38(Suppl. 1):D750–3, 2010. doi: 10.1093/nar/gkp889. URL http://dx.doi.org/10.1093/nar/gkp889.

[41] G. R. Mirams, C. J. Arthurs, M. O. Bernabeu, R. Bordas, J. Cooper, A. Corrias, Y. Davit, S.-J. Dunn, A. G. Fletcher, D. G. Harvey, M. E. Marsh, J. M. Osborne, P. Pathmanathan, J. Pitt-Francis, J. Southern, N. Zemzemi, and D. J. Gavaghan. Chaste: An open source C++ library for computational physiology and biology. *PLoS Comput Biol*, 9(3):1–8, 03 2013. doi: 10.1371/journal.pcbi.1002970. URL http://dx.doi.org/10.1371/journal.pcbi.1002970.

[42] M. Mugnano, A. Calabuig, S. Grilli, L. Miccio, and P. Ferraro. Monitoring cell morphology during necrosis and apoptosis by quantitative phase imaging, 2015. URL http://dx.doi.org/10.1117/12.2186771.

[43] I. Ramis-Conde, D. Drasdo, A. R. A. Anderson, and M. A. J. Chaplain. Modeling the influence of the E-cadherin-β-Catenin pathway in cancer cell invasion: A multiscale approach. *Biophys. J.*, 95(1):155–65, 2016. doi: 10.1529/biophysj.107.114678. URL http://dx.doi.org/10.1529/biophysj.107.114678.

[44] A. M. Schlitter, K.-T. Jang, G. Kloppel, B. Saka, S.-M. Hong, H. Choi, G. J. Offerhaus, R. H. Hruban, Y. Zen, B. Konukiewitz, I. Regel, M. Allgauer, S. Balci, O. Basturk, M. D. Reid, I. Esposito, and V. Adsay. Intraductal tubulopapillary neoplasms of the bile ducts: clinicopathologic, immunohistochemical, and molecular analysis of 20 cases. *Mod. Pathol.*, 28(9):1249–64, 2015. doi: 10.1038/modpathol.2015.61. URL http://dx.doi.org/10.1038/modpathol.2015.61.

[45] D. Schlüter, I. Ramis-Conde, and M. J. Chaplain. Computational modeling of single-cell migration: The leading role of extracellular matrix fibers. *Biophys. J.*, 103(6):1141–51, 2016. doi: 10.1016/j.bpj.2012.07.048. URL http://dx.doi.org/10.1016/j.bpj.2012.07.048.

[46] J. Starru, W. de Back, L. Brusch, and A. Deutsch. Morpheus: a user-friendly modeling environment for multiscale and multicellular systems biology. *Bioinformatics*, 30(9):1331–1332, 2014. doi: 10.1093/bioinformatics/btt772. URL http://dx.doi.org/10.1093/bioinformatics/btt772.

[47] R. L. Sutherland, R. E. Hall, and I. W. Taylor. Cell proliferation kinetics of MCF-7 human mammary carcinoma cells in culture and effects of tamoxifen on exponentially growing and plateau-phase cells. *Cancer Research*, 43(9):3998–4006, 1983. ISSN 0008-5472. URL http://cancerres.aacrjournals.org/content/43/9/3998.

[48] M. H. Swat, G. L. Thomas, J. M. Belmonte, A. Shirinifard, D. Hmeljak, and J. A. Glazier. Chapter 13 - multi-scale modeling of tissues using CompuCell3D. In A. R. Asthagiri and A. P. Arkin, editors, *Computational Methods in Cell Biology*, volume 110 of *Methods in Cell Biology*, pages 325 – 366. Academic Press, 2012. doi: 10.1016/B978-0-12-388403-9.00013-8. URL http://dx.doi.org/10.1016/B978-0-12-388403-9.00013-8.

[49] The Physical Sciences - Oncology Centers Network. A physical sciences network characterization of non-tumorigenic and metastatic cells. *Sci. Rep.*, 3:1449, 2013. doi: 10.1038/srep01449. URL http://dx.doi.org/10.1038/srep01449.

[50] A. Urruticoechea, I. E. Smith, and M. Dowsett. Proliferation marker Ki-67 in early breast cancer. *Journal of Clinical Oncology*, 23(28):7212–7220, 2005. doi: 10.1200/JCO.2005.07.501. URL http://jco.ascopubs.org/content/23/28/7212.abstract.

[51] P. Van Liedekerke, M. M. Palm, N. Jagiella, and D. Drasdo. Simulating tissue mechanics with agent-based models: concepts, perspectives and some novel results. *Computational Particle Mechanics*, 2(4):401–444, 2015. ISSN 2196-4386. doi: 10.1007/s40571-015-0082-3. URL http://dx.doi.org/10.1007/s40571-015-0082-3.

[52] M. C. Weiger, S. Ahmed, E. S. Welf, and J. M. Haugh. Directional persistence of cell migration coincides with stability of asymmetric intracellular signaling. *Biophysical journal*, 98(1):67–75, Jan. 2010. ISSN 1542-0086. doi: 10.1016/j.bpj.2009.09.051. URL http://dx.doi.org/10.1016/j.bpj.2009.09.051.

[53] Y. Zheng and T. L. Sheppard. Half-life and DNA strand scission products of 2-deoxyribonolactone oxidative DNA damage lesions. *Chemical Research in Toxicology*, 17(2):197–207, 2004. doi: 10.1021/tx034197v. URL http://dx.doi.org/10.1021/tx034197v.
