# Supplementary material for: PhysiCell: An open source physics-based cell simulator for 3-D multicellular systems
Source: PLoS Comput Biol. 2018 Feb 23;14(2):e1005991. doi: 10.1371/journal.pcbi.1005991 (PMC5841829; doi:10.1371/journal.pcbi.1005991)
Supplement: S2 Text — User Guide for PhysiCell 1.2.2. Includes full API documentation and examples. (PDF) [file pcbi.1005991.s002.pdf]

PhysiCell User Guide (Version 1.2.2)

PhysiCell Project (Lead: Paul Macklin)

Revision: November 4, 2017

Contents

1 Introduction and citing PhysiCell **4**

2 Getting started: The Quickstart and your First Simulation **4**

3 Further resources for help **5**

4 Preparing your development environment **5**
4.1 Special notes for OSX users 5
4.2 Virtual Machine option 6
4.3 Suggested tutorials and resources 6
4.3.1 Linux and Makefile tutorials 6
4.3.2 C++ references 7
4.3.3 Matlab tutorials 7
4.3.4 VirtualBox and related information (virtual appliances) 8
4.3.5 Recommended additional tools 8

5 Overall codebase structure **9**

6 Using project templates **10**

7 Using the Sample Projects **11**
7.1 Extra makefile rules 12

8 The BioFVM microenvironment **13**
8.1 A brief note on the microenvironment 13
8.2 Setting up and using the microenvironment 13
8.2.1 Setting BioFVM options 13
8.2.2 Adding new diffusing substrates to the tissue environment 15
8.2.3 Initializing the BioFVM tissue microenvironment 16
8.3 Sampling the microenvironment 17
8.4 Dirichlet conditions 18
8.4.1 Refined control of Dirchlet conditions 19
8.5 Other BioFVM resources 19

9 Cells 19
9.1 Other member data . . . 22
9.2 Member functions . . . 22
9.3 Other key functions . . . 25
9.4 Important classes (except Phenotype) . . . 26
9.4.1 Custom_Cell_Data . . . 26
9.4.2 Cell_Parameters . . . 30
9.4.3 Cell_Functions . . . 32
9.4.4 Cell_State . . . 34
9.4.5 Cell Definition . . . 35

10 Phenotype 36
10.1 Member functions . . . 37

11 Phenotype details 37
11.1 Cycle Models . . . 37
11.1.1 Phase . . . 38
11.1.2 Phase_Link . . . 39
11.1.3 Cycle_Model . . . 40
11.1.4 Cycle_Data . . . 42
11.1.5 Cycle . . . 44
11.2 Death models . . . 45
11.2.1 Death_Parameters . . . 45
11.2.2 Death . . . 46
11.3 Volume . . . 48
11.3.1 Member functions . . . 50
11.4 Geometry . . . 50
11.4.1 Member functions . . . 52
11.5 Mechanics . . . 52
11.5.1 Member functions . . . 53
11.6 Motility . . . 53
11.6.1 Member functions . . . 54
11.6.2 Motility definitions . . . 54
11.7 Secretion . . . 55
11.7.1 Member functions . . . 56

12 Cell Containers 56

13 PhysiCell Outputs 58
13.1 Virtual Pathology . . . 58
13.1.1 SVG functions . . . 58
13.1.2 Pathology functions . . . 60
13.1.3 Cell coloring functions . . . 61
13.1.4 Examples of custom cell coloring functions . . . 63
13.2 MultiCellDS digital simulation snapshots . . . 64
13.2.1 Reading PhysiCell snapshots in MATLAB . . . 67
13.3 Other outputs . . . 68

14 Key initialization functions 69

15 Key global data structures **69**
15.1 Global strings . . . 69
15.2 Default microenvironment . . . 69
15.3 Default cell definition . . . 70
15.4 A list of all cells . . . 71
15.5 MultiCellDS options . . . 71
15.5.1 Software metadata . . . 72
15.5.2 Citation metadata . . . 73
15.5.3 Person metadata . . . 73
15.6 SVG options . . . 74
15.7 PhysiCell Constants . . . 74

16 Standard models **76**
16.1 Cell Cycle Models . . . 77
16.1.1 Live (`live`) code: `PhysiCell_constants::live_cells_cycle_model`) . . . 77
16.1.2 Ki-67 Basic (`Ki67_basic`) (Code: `PhysiCell_constants::basic_Ki67_cycle_model`) . . . 77
16.1.3 Ki-67 Advanced (`Ki67_advanced`) (code: `PhysiCell_constants::advanced_Ki67_cycle_model`) . . . 78
16.2 Death Cycle Models . . . 79
16.2.1 Apoptosis (`apoptosis`) (code: `PhysiCell_constants::apoptosis_death_model`) . . . 79
16.2.2 Necrosis (`necrosis`) (code: `PhysiCell_constants::necrosis_death_model`) . . . 80
16.3 Volume model (`standard_volume_update_function`) . . . 81
16.4 Cell velocity model (`standard_update_cell_velocity`) . . . 81
16.5 Up orientation model (`up_orientation`) . . . 81
16.6 Oxygen-dependent phenotype (`update_cell_and_death_parameters_O2_based`) . . . 81

17 Examples **82**
17.1 Working with `Cell` functions . . . 82
17.1.1 Example: a custom volume model . . . 82
17.1.2 Example: a custom migration bias . . . 83
17.1.3 Example: a custom cell rule . . . 84
17.1.4 Example: a custom phenotype update function . . . 85
17.1.5 Example: a custom velocity update function . . . 86
17.1.6 Example: analytical basement membrane functions . . . 86
17.1.7 Example: a custom cell orientation function . . . 86
17.2 Cell cycle models . . . 86
17.2.1 Creating a custom cell cycle model . . . 86
17.2.2 Adding an arrest function . . . 87
17.2.3 Adding a custom phase entry function . . . 88

18 Future **89**

19 Some notes on parameter values **89**
19.1 Live cycle model . . . 89

19.2 Ki67 Basic model . . . 90
19.3 Ki67 Advanced model . . . 91

20 Acknowledgements **91**

Bibliography **91**

1 Introduction and citing PhysiCell

This user guide will teach you how to download and use PhysiCell [3], as well as document the key classes and functions. Wherever possible, it will demonstrate with specific examples. Please note that this guide will be periodically updated. Users should check PhysiCell.MathCancer.org for the latest version. The PhysiCell method paper is in revision for additional peer review at PLoS Computational Biology [3].

If you use PhysiCell, please cite it as:

> We built our model using PhysiCell (Version 1.2.2) [1].
>
> [1] A. Ghaffarizadeh, R. Heiland, S.H. Friedman, S.M. Mumenthaler, and P. Macklin, PhysiCell: an Open Source Physics-Based Cell Simulator for 3-D Multicellular Systems, bioRxiv 088773, 2017. DOI: 10.1101/088773.

We will update this citation information as PhysiCell progresses through peer review.

If you prefer to cite a peer-reviewed journal publication while waiting for our main method paper, you you can also cite an earlier (2-D) version of the code by our 2012 paper in the Journal of Theoretical Biology:

> P. Macklin, M.E. Edgerton, A.M. Thompson, and V. Cristini. Patient-calibrated agent-based modelling of ductal carcinoma in situ (DCIS): From microscopic measurements to macroscopic predictions of clinical progression. J. Theor. Biol. 301:122-40, 2012. DOI: 10.1016/j.jtbi.2012.02.002.

[Return to Table of Contents.]

2 Getting started: The Quickstart and your First Simulation

As of Version 1.2.2, every download of PhysiCell includes a `Quickstart.pdf` guide in the root directory. If you follow the instructions in that guide (along with instructions to set up your compiler and environment; see Section 4), you should be able to run and visualize your first PhysiCell simulation (heterogeneous tumor growth) in well under an hour.

You should also watch the #PhysiCell hashtag on Twitter for updates on PhysiCell and new tricks, tips, and tutorials. Tutorials and other blog posts can be found at http://MathCancer.org/blog/physicell-tutorials/. See Section 3 for resources for help, including support tickets and the PhysiCell blog.

[Return to Table of Contents.]

3 Further resources for help

The PhysiCell project posts tips and tutorials at its blog:

http://www.mathcancer.org/blog/physicell-tutorials/

Users are encouraged to frequently visit the blog for these tips. This user manual may be updated more frequently than PhysiCell. Please check the PhysiCell project website for updates:

http://PhysiCell.MathCancer.org

Lastly, users can support help tickets at SourceForge:

https://sourceforge.net/p/physicell/tickets/

[Return to Table of Contents.]

4 Preparing your development environment

PhysiCell was designed to be cross-platform compatible, without need for a package manager system or any other complex installation. In principle, any `C++11` compliant compiler with OpenMP support should work. In practice, we use `g++` as our "gold standard." You'll also want to ensure that your build environment supports makefiles. Command-line zip/unzip is also helpful, but not required.

Please note that OSX (and its associated developer tools) ships with "`g++`" instead of `g++`: it uses LLVM/Clang and an alias to *pretend* to be `g++`. Unfortunately, the version of LLVM/Clang that Apple ships does not fully support OpenMP, and so compiling can fail on those platforms without further setup. For OSX, we recommend following our Homebrew-based tutorial to install real `g++`.

Full tutorials on installing a 64-bit `g++`/OpenMP environment (on Windows via mingw-w64 and on OSX using Homebrew) can be found at:

http://www.MathCancer.org/blog/physicell-tutorials/

Most linux users should already have a 64-bit `g++` environment installed by default.

[Return to Table of Contents.]

4.1 Special notes for OSX users

As of Version 1.2.2, OSX users no longer need to modify the `CC` definition in the makefiles–this represents a significant simplification for those users. However, OSX users (including those who have already installed `g++` by Homebrew of MacPorts) need to perform a one-time setup of an environment variable. Open your terminal, and run the following commands:

```
export PHYSICELL_CPP=your_compiler_name
echo export PHYSICELL_CPP=your_compiler_name >> ~/.bash_profile
```

`your_compiler_name` will be something like `g++-7` for Homebrew installations, and something like `g++-mp-7` for MacPorts installations.

See the tutorials at http://www.MathCancer.org/blog/physicell-tutorials/ for more details. Also, if you have compiler crashes, see the FAQ (frequently asked questions) at:

http://www.mathcancer.org/blog/common-problems-and-solutions/

[Return to Table of Contents.]

4.2 Virtual Machine option

Starting with the 1.2.x releases, we began distributing PhysiCell as zipped source (preferred) and virtual appliances for use in VirtualBox and other virtual machine software, allowing us to distribute a full PhysiCell development environment (including 64-bit `g++`, support for makefiles, zip/unzip, ImageMagick, mencoder, eog, a text editor, and the most up-to-date version of PhysiCell). This should make it simpler to start learning and using PhysiCell, even in cases where developers are not free to install or modify their own build environments, or have difficulty installing and configuring `g++`.

Please visit the PhysiCell blog for information on running VirtualDub and using the virtual appliance. (Section 3).

[Return to Table of Contents.]

4.3 Suggested tutorials and resources

Working in PhysiCell requires some knowledge of `C++`, Makefiles, and command-line routines in Unix-like systems. If you are not familiar with these skillsets, we recommend the following resources:

4.3.1 Linux and Makefile tutorials

1. **A tutorial on learning the Linux shell:** http://linuxcommand.org/lc3_learning_the_shell.php
   In particular, I recommend:
   (a) What is "The Shell"?
   (b) Navigation
   (c) Looking Around
   (d) Manipulating Files
2. **UNIX Tutorial for Beginners:** http://www.ee.surrey.ac.uk/Teaching/Unix/
   In particular, I suggest the following:
   (a) Tutorial 1 (navigation, creating directories, etc.)
   (b) Tutorial 2 (moving, copying files, etc.)

(c) Tutorial 4 (wildcards, filename conventions, etc.)
(d) Tutorial 7 (compiling and runnign software, etc.)
(e) Tutorial 8 (UNIX and environment variables, etc.) (**Note:** May not work in Windows/MinGW.)

3. **The GNU Bash Reference Manual:** http://www.gnu.org/software/bash/manual/bash.html
4. **A good makefile tutorial:** http://www.cprogramming.com/tutorial/makefiles.html
5. **Another good makefile tutorial:** http://mrbook.org/blog/tutorials/make/
6. **One more makefile tutorial:** http://makefiletutorial.com/
7. **The GNU Make Reference:** https://www.gnu.org/software/make/manual/make.html

---

[Return to Table of Contents.]

4.3.2 C++ references

The following websites are good references for `C++`:

1. **CPlusPlus.com:** http://www.cplusplus.com/
   Excellent, detailed documentation on `C++`, as well as tutorials.
2. **C++ Reference:** http://en.cppreference.com/w/
   Another good reference guide to `C++`.
3. **LearnCpp.com:** http://www.learncpp.com/
   A series of tutorials on `C++`.
4. **StackOverflow (C++ tag):** https://stackoverflow.com/questions/tagged/c%2b%2b
   A sometimes overwhelming resource for problem solving in `C++`.
5. **C++ section on CodeGuru:** http://www.codeguru.com/cpp/cpp/

[Return to Table of Contents.]

4.3.3 Matlab tutorials

If you use Matlab for visualization and/or postprocessing, we recommend:

1. **MATLAB tutorials at Mathworks:**
   https://www.mathworks.com/academia/student_center/tutorials/mltutorial_launchpad.html
2. **Introduction to MATLAB for Engineering Students:** http://bit.ly/2cQBXDc
3. **A Beginner's Guide to MATLAB:** http://homen.vsb.cz/~lud0016/NM/matlab_guide.pdf
   (By Christos Xenophontos at Loyola College).
4. **MATLAB Academy:** https://matlabacademy.mathworks.com/?s_eid=ppc_23223967642

5. **MATLAB Tutorial:** https://www.tutorialspoint.com/matlab/
6. **Octave:** If you do not have access to MATLAB or prefer an open source alternative, have a look at this cross-platform package:
   https://www.gnu.org/software/octave/

[Return to Table of Contents.]

4.3.4 VirtualBox and related information (virtual appliances)

If you use the virtual appliance, we suggest the following tutorials and resources:

1. **VirtualBox:** http://virtualbox.org
   This is an excellent cross-platform (Windows, Linux, OSX, etc.) virtual machine software that can import the virtual appliance version of PhysiCell.
2. **Alpine Linux:** https://alpinelinux.org/
   Alpine Linux is a lean and secure version of linux we installed in the PhysiCell virtual appliance.
3. **Alpine Linux Wiki:** https://wiki.alpinelinux.org
   A helpful site for using Alpine Linux.
4. **VirtualBox shared folders in Alpine:** https://wiki.alpinelinux.org/wiki/VirtualBox_shared_folders
5. **Alpine Linux users forum:** https://forum.alpinelinux.org/
6. **XFCE4 project:** https://xfce.org/
   We use the XFCE4 desktop environment in our virtual appliance, so this may be helpful.

[Return to Table of Contents.]

4.3.5 Recommended additional tools

Lastly, we find the following tools and resources very useful for postprocessing and visualization:

1. **ImageMagick** is a cross-flatform image manipulation suite, which can (among other things) resize and crop images, change formats, and insert text labels. In PhysiCell, ImageMagick is especially useful for converting SVG files to PNG, JPG, or other raster-based formats for further animation.
   - **URL:** http://imagemagick.org
   - **Typical use:** `magick mogrify -format jpg snapshot*.svg`
     (Converts all files of the form snapshot*.svg to the png format.)
   - **Typical use:** `magick snapshot*.jpg animation.gif`
     (Combines all snapshot*.jpg files into a (huge!) animated gif file.)
2. **Mencoder** is a cross-platform, open source mpeg encoder, useful for creating compressed movies. In PhysiCell, we use mencoder to convert series of simulation snapshots to movies.

- **URL (Linux):** Use your package manager to install mplayer (which includes mencoder).
- **URL (OSX):** Use Homebrew or MacPorts to install mplayer (which includes mencoder).
- **URL (Windows):** http://mplayerwin.sourceforge.net/
- **Typical use:**
```
mencoder "mf://snapshot*.jpg" -ovc lavc -lavcopts
 vcodec=mpeg4:vbitrate=10000:mbd=2:trell -mf fps=24:type=jpg -nosound -o out.avi
```
(Converts all the snapshot*.jpg files into an mpeg4-encoded movie named out.avi, with a 8kbps variable bit rate and 24 frames per second.)

[Return to Table of Contents.]

---

5 Overall codebase structure

PhysiCell was created by extending the `Basic_Agent` class in BioFVM [2] to include a fuller cell phenotype and force-based cell motion. In the overall software philosophy, we structure PhysiCell in several critical subdirectories:

1. **`BioFVM`** This includes a working copy of the BioFVM multi-substrate diffusion code [2]. Users should be able to fully replace these files with any later version of BioFVM. Note that BioFVM distributions also include pugixml (an efficient cross-platform XML parser) [6].

2. **`config`** While this directory is not currently used, PhysiCell will eventually parse XML files with configuration information in this directory. Any custom configuration files should be placed in this directory.

3. **`core`** The core library files for PhysiCell are kept in this directory. Users should not modify functions in the core library.

4. **`custom_modules`** This directory is the right place to put custom user code. Moreover, users should list their custom code files in `custom_modules/PhysiCell_custom.h`.

5. **`modules`** This is where we place non-core (but helpful) standard code modules for distribution with PhysiCell. Currently, we include pathology, MultiCellDS [1], and SVG (scalable vector graphic) functions. Future releases may include basic modules for extracellular matrix.

The following subdirectories are also included:

1. **`archives`** If you use the `make zip` or `make archive` rules, the compressed archives will be placed here.

2. **`documentation`** This directory includes user guides (like this one!).

3. **`matlab`** This includes basic Matlab files for handling PhysiCell outputs. (This and other postprocessing will be a major PhysiCell focus over the next few years.)

4. **`examples`** The examples from the PhysiCell method paper will be placed here. They may not necessarily be updated for compatibiltiy with every PhysiCell release. (They are currently compatible up to version 1.1.1.)

5. **licenses** License files for BioFVM [2], pugixml [6], PhysiCell, and any other (BSD-compatible) dependencies are kept here.

6. **output** Some examples will put their outputs here.

7. **povray** This includes some basic utilities for visulization by povray (an open source raytracing package) [9].

8. **template_projects** This directory once stored template projects for 2D and 3D. These have been moved to the sample projects as of Version 1.2.2. This directory will be deprecated.

9. **sample_projects** This directory includes sample projects, including 2D and 3D template projects and at least the four sample projects in [3]. A good start to a new project is to use the `make template2D` or `make template3D` makefile rules, which will populate the `main.cpp`, `Makefile`, and `./custom_modules` with appropriate codes to get started. See Section 6 for more information on the template projects, and Section 7 for the full list of sample projects and instructions to build them.

[Return to Table of Contents.]

---

6 Using project templates

As of Version 1.1.0, PhysiCell includes templates for 2-D and 3-D projects. These template projects set up all the critical BioFVM (microenvironment) and PhysiCell data structures and give examples on seeding a few cells. When beginning a new project with PhysiCell, we strongly recommend starting with these templates.

To start a new 2-D project (based on the template), go to the command line in the root PhysiCell directory, and run:

```
make template2D
```

This will populate a starting project from the files in `./sample_projects/template2D/`. In particular, it will overwrite the `Makefile`, copy `template_projects/template2D.cpp` to `main.cpp` in the root directory, and copy the contents of `./sample_projects/template2D/custom_modules/` to `./custom_modules`. Note that the `Makefile` has been modified to include the custom modules:

```
# put your custom objects here (they should be in the custom_modules directory)

PhysiCell_custom_module_OBJECTS := custom.o

##### and later in the Makefile ...

# user-defined PhysiCell modules

custom.o: ./custom_modules/custom.cpp
    $(COMPILE_COMMAND) -c ./custom_modules/custom.cpp
```

In general, your project should modify main.cpp, but primarily add custom codes to `./custom_modules`, just as this example. (For example, you might define your own angiogenesis functions in `./custom_modules/angiogenesis.h`, implement the functions in `./custom_modules/angiogenesis.cpp`, and then modify the `Makefile` to include it:

```
# put your custom objects here (they should be in the custom_modules directory)

PhysiCell_custom_module_OBJECTS := custom.o angiogenesis.o

##### and later in the Makefile ...

# user-defined PhysiCell modules

custom.o: ./custom_modules/custom.cpp
    $(COMPILE_COMMAND) -c ./custom_modules/custom.cpp

angiogenesis.o: ./custom_modules/angiogenesis.cpp
    $(COMPILE_COMMAND) -c ./custom_modules/angiogenesis.cpp
```

This is the recommended structure for a project.

Once you're ready, build the project by typing

`make`

By default, the executable will be called `project2D` (`project2D.exe` on windows). To change the name of the executable, modify the `PROJECT_NAME` variable in the `Makefile`.

To start a new 3-D project based on a template, type `make template3D` and continue as before.

Now, run the code:

`./project2D`

(in Windows, omit the "`./`".) This will generate (among other things) a series of SVG images that visualize the simulation once per hour.

More examples will be introduced on the PhysiCell blog. See Section 3.

[Return to Table of Contents.]

7 Using the Sample Projects

PhysiCell includes several sample projects to illustrate capabilities and suggest modeling possibilities. In general, building and running these projects consists of the following steps:

1. **Populate a project:** Use the following Makefile rule (from a terminal / command project in your PhysiCell root directory):

   `make [project_name]`

   where `[project_name]` is one of the sample projects listed below. For example, to populate the "cancer biorobots" sample project:

   `make cancer-biorobots-sample`

2. **Build the project:** Just run make:

   `make`

3. **Run the project:** Run the executable created by the compiler. If the name of the program is `[PROGRAM_NAME]`, run

   `./[PROGRAM_NAME]`

   (Windows users should omit the `./`.)

   One simple way to determine the name of the executable is to use grep on the Makefile:

   `grep PROGRAM_NAME Makefile`

   For example, to run the cancer biorobots example, the executable name is `cancer_biorobots`, so you run:

   `./cancer_biorobots`

   The project will create a series of SVG images files, as well as MultiCellDS save files (a combination of matlab and XML files). See 13.2.1 and the PhysiCell blog.

4. **(Optional) Clear out the project / return to a clean slate:** If you want to build and run a different sample project, or clear out the sample materials to create your own, you need to "de-populate" the sample project:

   `make reset`

Here are the sample projects included as of Version 1.2.2:

1. **`biorobots-sample`** is a 2-D example of 3 cell types: cargo cells, worker cells (which seek out cargo cells and haul them to a destination), and director cells (which attract worker cells).

2. **`cancer-biorobots-sample`** extends the biorobots example to (1) simulate a 2-D tumor that develops a necrotic core, (2) so that cargo cells are hauled towards hypoxic tumor regions, and (3) so that released cargo cells secrete a chemotherapeutic compounds.

3. **`heterogeneity-sample`** simulates a 2-D tumor with heterogeneous "genetics" that drive differential proliferation.

4. **`cancer-immune-sample`** extends the heterogeneity example to 3D, and introduces a new immune cell type (modeling T cells) that migrate towards tumor cells, temporarily adhere, test for immunogenicity, and initiate apoptosis.

5. **`template2D`** is the template for a 2-D project. See Section 6.

6. **`template3D`** is the template for a 3-D project. See Section 6.

[Return to Table of Contents.]

7.1 Extra makefile rules

To clear out the data generated in a simulation:

`make data-cleanup`

To clear out the compiler-generated objects (which would allow you to recompile the entire project):

`make clean`

[Return to Table of Contents.]

8 The BioFVM microenvironment

PhysiCell is built upon BioFVM [2], and so it uses the (bio)chemical microenvironment from BioFVM, including diffusion, decay, cell-based secretions/uptake, and bulk supply/uptake functions. All PhysiCell projects already include bioFVM once you have included PhysiCell:

```
#include "./core/PhysiCell.h"
```

We also suggest using the BioFVM and PhysiCell namespaces:

```
using namespace BioFVM;
using namespace PhysiCell;
```

8.1 A brief note on the microenvironment

BioFVM divides the simulation domain into a collection of non-intersecting **voxels**: volumetric pixels. Each voxel has a unique integer **index**: this is its unique address, for quickly accessing its information. As particularly notable information, each voxel stores its own position (center) and volume.

BioFVM adds one or more diffusible substrates to this microenvironment. Each substrate has a diffusion coefficient and decay rate. At present, these are homogeneous throughout the microenvironment, although improvements are planned for BioFVM to have spatially variable diffusion coefficients. In BioFVM, each substrate diffuses and decays, and can be secreted or uptaken by individual cells (see Section 11.7) at their individual positions. You can also set bulk uptake and secretion functions, which are applied in each voxel. These are not used in the current template and sample PhysiCell projects. See [2] for more information.

For each voxel, we store a vector of chemical substrates (densities), and a vector of gradients (one gradient vector for each substrate). Moreover, users can use "Dirichlet nodes" to overwrite the substrate values at any voxel within the simulation domain. This is useful for modeling biotransport in irregular domains, setting substrate values along blood vessels, or applying classical Dirichlet conditions along the outer edges of the simulation domain. Note that without specifying Dirichlet conditions, BioFVM applies Neumann (no flux) conditions at the outer simulation boundaries.

[Return to Table of Contents.]

8.2 Setting up and using the microenvironment

Within your main program loop (`int main( int argc, char* argv[])`), you need to declare and set up a Microenvironment. (Here, we also include some useful tips for setting space and time units.) We make use of BioFVM 1.1.5 improvements that include simplified setup functions.

8.2.1 Setting BioFVM options

BioFVM 1.1.5 and later versions includes a data structure (`default_microenvironment_options`, of type `Microenvironment_Options`) for setup options. This data type is defined in `BioFVM_microenvironment.h` as:

```
class Microenvironment_Options
{
 private:

 public:
    Microenvironment* pMicroenvironment;
    std::string name;

    std::string time_units;
    std::string spatial_units;
    double dx;
    double dy;
    double dz;

    bool outer_Dirichlet_conditions;
    std::vector<double> Dirichlet_condition_vector;
    std::vector<bool> Dirichlet_activation_vector;

    bool simulate_2D;
    std::vector<double> X_range;
    std::vector<double> Y_range;
    std::vector<double> Z_range;

    Microenvironment_Options();

    bool calculate_gradients;

    bool use_oxygen_as_first_field;
};
```

You can set these options either towards the top of your main program source file (e.g., in `main.cpp`) or in a standalone setup function. See the sample projects for examples. Here is sample use, to set the tissue name to "liver" and set up to 3D with Dirichlet conditions of oxygen = 38 mmHg:

```
/* Microenvironment setup */

default_microenvironment_options.name = "liver";
default_microenvironment_options.time_units = "min";
default_microenvironment_options.spatial_units = "micron";
default_microenvironment_options.dx = 20;
default_microenvironment_options.dy = 20;
default_microenvironment_options.dz = 20;

// set a Dirichlet outer boundary condition
default_microenvironment_options.outer_Dirichlet_conditions = true;

std::vector<double> bc_vector( 1 , 38.0 ); // 5% o2
default_microenvironment_options.Dirichlet_condition_vector = bc_vector;
```

```
// stick with a 3-D simulation
default_microenvironment_options.simulate_2D = false;

// set the domain size
default_microenvironment_options.X_range = {-500, 500};
default_microenvironment_options.Y_range = {-400, 400};
default_microenvironment_options.Z_range = {-100, 100};

// turn off gradient calculations
default_microenvironment_options.bool calculate_gradients = false;
```

[Return to Table of Contents.]

8.2.2 Adding new diffusing substrates to the tissue environment

By default, the BioFVM microenvironment has a single substrate (with index 0). To add (append) a new substrate to the microenvironment, use one of these functions:

1. **`void Microenvironment::add_density( void )`** Use this to add a new diffusing substrate (density), without setting any of its properties.
2. **`void Microenvironment::add_density( std::string name , std::string units )`** Use this to add a new substrate called `name` with units `units`. (e.g., crayons with units Megacrayolas).
3. **`void Microenvironment::add_density( std::string name , std::string units, double diffusion_constant, double decay_rate )`** acts similarly as above, but also sets the diffusion coefficient and decay rate. Generally, these shoudl be in the same units as the simulation: by default, μm^2/min for diffusion and 1/min for decay.

You can also change the name, units, and properties for an existing substrate:

1. **`void Microenvironment::set_density( int index , std::string name , std::string units )`** This function renames the substrate with index `index` to `name`, and sets its units to `units`.
2. **`void Microenvironment::set_density( int index , std::string name , std::string units , double diffusion_constant , double decay_rate )`** works as above, but also sets the diffusion coefficient and decay rate.

For example,

```
// add a chemoattractant

microenvironment.add_density( "chemoattractant", "dimensionless" );
microenvironment.diffusion_coefficients[1] = 1e3;
microenvironment.decay_rates[1] = .1;
```

```
// add a therapeutic compound

microenvironment.add_density( "drug", "dimensionless" );
microenvironment.diffusion_coefficients[2] = 1e3;
microenvironment.decay_rates[2] = 0.15625;

// rename the first density to be glucose, and change parameters

microenvironment.set_density( "glucose", "dimensionless" );
microenvironment.diffusion_coefficients[0] = 1e3;
microenvironment.decay_rates[0] = 0.05625;
```

You should always add or modify your substrates prior to initializing the microenvironment. See Section 8.2.3

[Return to Table of Contents.]

8.2.3 Initializing the BioFVM tissue microenvironment

```
// initialize BioFVM with these options
initialize_microenvironment();
```

BioFVM defaults to a 1 mm^3 domain centered at (0,0,0) with $\Delta x = \Delta y = \Delta z = 20\ \mu$m, simulating 3D with no Dirichlet conditions and no gradient calculations, and minutes time units and micron space units. If you call `initialize_microenvironment()` without setting units (see Section 8.2.1), these defaults will be used.

The code also automatically chooses the correct 2D/3D diffusion solver, and sets the single diffusing field to oxygen with diffusion coefficient $10^5 \mu\mathrm{m}^2$/min and decay coefficient 0.1 1/min (for a 1 mm diffusion length scale in the absence of cells).

By the end of these commands, the default `Microenvironment` is set to `microenvironment`. You can get this address at any time using the BioFVM command:

`Microenvironment* get_default_microenvironment( void )`

You'll also need to set up PhysiCell's mechanics data structures (for cell-cell interaction testing) and match them to BioFVM:

```
/* PhysiCell setup */

// prepare PhysiCell mechanics data structures

// mechanics voxel size
double mechanics_voxel_size = 30;
Cell_Container* cell_container = create_cell_container_for_microenvironment(
   microenvironment, mechanics_voxel_size );
```

Within your main program loop, you'll want to make sure that BioFVM is being called to update the biochemical environment:

```
while( t < t_max )
{
   // main loop contents ...

   // update the microenvironment

   microenvironment.simulate_diffusion_decay( diffusion_dt );
   if( default_microenvironment_options.calculate_gradients )
   { microenvironment.compute_all_gradient_vectors(); }

   // physicell functions

   t += diffusion_dt;
}
```

As of Version 1.2.0, PhysiCell has automated cell secretions/uptake as part of its phenotype updates. There is no need to explicitly call the BioFVM cell-based functions in the main program loop.

Note that the PhysiCell template projects and sample projects already include these critical setup commands. See Section 6.

[Return to Table of Contents.]

8.3 Sampling the microenvironment

To make it easier to write functions involving microenvironment substrates at any cell's location, BioFVM 1.1.5 added the following function:

`int Microenvironment::find_density_index( std::string name )` This allows you to find the index of the substrate named `name`. Note that this function is case sensitive.

The following functions can access the substrates and their gradients in space:

`int nearest_voxel_index( std::vector<double>& position )` This returns the integer that identifies the microenvironment voxel containing the point at `position`.

`std::vector<double>& nearest_density_vector( std::vector<double>& position )` This function returns the vector of substrates located nearest to `position`.

`std::vector<double>& nearest_density_vector( int voxel_index )` This function returns the vector of substrates stored in the voxel with index `voxel_index`. See the `nearest_voxel_index`

`std::vector<double>& operator()( int n )` This function can directly access the density vector stored at voxel index `n`.

`std::vector<gradient>& gradient_vector(int n )` This function accesses the vector of gradient vectors at voxel index `n`.

`std::vector<gradient>& gradient_vector(std::vector<double>& position )` This function accesses the vector of gradient vectors at `position`.

Here's an example of these functions in action:

```
bool bad_conditions( Microenvironment& M , int voxel_index )
{
    // find the correct indices the first time you run this function
    static int oxygen_index  = M.find_density_index( "oxygen" );
    static int glucose_index = M.find_density_index( "glucose" );

    // find the oxygen gradient

    std::vector<double> gradient = M.gradient_vector(voxel_index)[oxygen_index];

    static double oxygen_threshold = 2.5; // mmHg
    static double glucose_threshold = 0.05; // dimensionless

    if( M(voxel_index)[oxygen_index]  < oxygen_threshold &&
        M(voxel_index)[glucose_index] < glucose_threshold )
    { return true; }

    return false;
}
```

There are several functions to help sample the microenvironment at a cell's position. See Section 9.

[Return to Table of Contents.]

8.4 Dirichlet conditions

BioFVM also allows you to set constant conditions at any voxel. (These are called Dirichlet nodes.) The relevant functions are:

1. **`void Microenvironment::add_dirichlet_node( int voxel_index, std::vector<double>& value )`** adds a Dirichlet node at voxel `voxel_index`, so that (for some `Microenvironment M`):

   `M(voxel_index) = value`.

2. **`void Microenvironment::update_dirichlet_node( int voxel_index , std::vector<double>& new_value )`** overwrites the (vector) value of the Dirichlet node at `voxel_index`, so that

   `M(voxel_index) = new_value`.

   If the voxel was not previously a Dirichlet node, it is automatically changed to a Dirichlet node.

3. **`void Microenvironment::remove_dirichlet_node( int voxel_index )`** removes the Dirichlet node at voxel `voxel_index`.

4. **`void Microenvironment::apply_dirichlet_conditions( void )`** applies the previously set Dirichlet conditions at all Dirichlet nodes.

5. **`bool& Microenvironment::is_dirichlet_node( int voxel_index )`** returns `true` if there is a Dirichlet node at voxel `voxel_index`.

BioFVM applies these Dirichlet conditions every time it evaluates the diffusion solver.

[Return to Table of Contents.]

8.4.1 Refined control of Dirchlet conditions

As of Version 1.2.1, you can now now control Dirichlet conditions on a substrate-by-substrate basis. That is, you can apply the Dirichlet condition to just one or two substrates (e.g., oxygen in index 0 and doxorubicin in index 12), while not applying them to the remaining substrates. For a microenvironment `M`, you can set these options in the `default_microenvironment_options` (see Section 8.2.1) prior to initializing the microenvironment, or at later times using the function

`void Microenvironment::set_substrate_dirichlet_activation( int substrate_index , bool new_value )` This function sets the substrate at index `substrate_index` to have/not have a Dirichlet condition based on the `true`/`false` value of `new_value`.

For example:

```
\\ set options for the substrate with index 2 to false

default_microenvironment_options.Dirichlet_activation_vector[2] = false;

\\ initialize the microenvironment with the currently set options

initialize_microenvironment();

\\ set the Dirichlet conditions in substrate 1 to true.

get_default_microenvironment()->set_substrate_dirichlet_activation(1,true);
```

Note that turning on or off a Dirichlet condition for a substrate applies it at all Dirichlet nodes for which `is_dirichlet_node(int voxel_index)` is `true`.

[Return to Table of Contents.]

8.5 Other BioFVM resources

To learn more about using BioFVM, take a look at the tutorials at:

http://mathcancer.org/blog/biofvm-tutorials/

[Return to Table of Contents.]

9 Cells

Each `Cell` is an extension of BioFVM's `Basic_Agent` class. As such, it has access to all the parent class's member data and functions. In PhysiCell, Cells have the following major parts:

1. **std::string type_name:** The name of the type of cell.
2. **Custom_Cell_Data custom_data:** Custom data attached the cell, which may differ from other cells. See Section 9.4.1.
3. **Cell_Parameters parameters:** A set of standardized parameters (which may eventually be moved into the cell's phenotype). See Section 9.4.2.
4. **Cell_Functions functions:** A collection of functions used for updating the cell phenotype, including custom functions. See Section 9.4.3.
5. **Cell_State cell_state:** A small set of standard state variables, chiefly (basal-to-apical) orientation. See Section 9.4.4.
6. **Phenotype phenotype:** A hierarchical organization of the cell's properties. This data element is discussed in great depth in Section 10.

The following inherited member data and functions (from `Basic_Agent`) are especially helpful:

1. **int ID** is a unique integer identifier for the cell. No other cell now or in the future will have the same `ID`.
2. **int index** is the cell's current index in `std::vector<Basic_Agent*> all_basic_agents`, the list of all current `Basic_Agents` in the simulation.
3. **int type** is a user-specified (default 0) integer code to classify the cell's type. Use these to quickly compare if two cells are of the same or different types, or to perform type-specific operations on them.
4. **int get_current_voxel_index(void)** returns the cell's positional index in the BioFVM Microenvironment.
5. **std::vector<double>& nearest_density_vector(void)** allows the user to directly access (i.e., sample or modify) the vector of substrates at the cell's position. This is useful building functions that alter cell phenotype based on the microenvironment.
6. **std::vector<double>& nearest_gradient(int substrate_index)** returns (by reference) the gradient of the substrate with index `substrate_index`, at the cell's current position. This is useful for things like chemotaxis.
7. **std::vector<gradient>& nearest_gradient_vector(void)** returns a vector of *all* the substrate gradients at the cell's position. Each `gradient` is a `std::vector<double>` of size 3.

Here is how the `Cell` class is defined in `PhysiCell_cell.h`:

```
class Cell : public Basic_Agent
{
 private:
    Cell_Container * container;
    int current_mechanics_voxel_index;
    int updated_current_mechanics_voxel_index;
```

```
public:
   std::string type_name;

   Custom_Cell_Data custom_data;
   Cell_Parameters parameters;
   Cell_Functions functions;

   Cell_State state;
   Phenotype phenotype;

   void update_motility_vector( double dt_ );
   void advance_bundled_phenotype_functions( double dt_ );

   void add_potentials(Cell*);
   void set_previous_velocity(double xV, double yV, double zV);
   int get_current_mechanics_voxel_index();
   void turn_off_reactions(double);

   bool is_out_of_domain;
   bool is_movable;

   void flag_for_division( void );
   void flag_for_removal( void );

   void start_death( int death_model_index );

   Cell* divide( void );
   void die( void );
   void step(double dt);
   Cell();

   bool assign_position(std::vector<double> new_position);
   bool assign_position(double, double, double);
   void set_total_volume(double);

   double& get_total_volume(void);

   // mechanics
   void update_position( double dt );
   std::vector<double> displacement;

   void assign_orientation();

   void copy_function_pointers(Cell*);

   void update_voxel_in_container(void);
   void copy_data(Cell *);
```

```
    void set_phenotype( Phenotype& phenotype );
    void update_radius();
    Cell_Container * get_container();

    std::vector<Cell*>& cells_in_my_container( void );

    void Cell::convert_to_cell_definition( Cell_Definition& cd )
};
```

[Return to Table of Contents.]

9.1 Other member data

1. **bool is_out_of_domain** is `true` if the cell is out of the simulation domain boundaries.
2. **bool is_movable** indicates whether the cell is in a static position. Set this to `true` if you would like PhysiCell to leave its position fixed and not evaluate the mechanics models for this cell. Note that it can still exert adhesive and repulsive forces on other cells.

[Return to Table of Contents.]

9.2 Member functions

1. **void update_motility_vector(double dt_)** updates the cell's motility vector, based on the following model:

   First, check if the cell should change its direction of motility $\mathbf{v}_{\mathrm{mot}}$ within the next Δt time:

   (a) If `is_motile == false` set $\mathbf{v}_{\mathrm{mot}} = \mathbf{0}$ and exit.

   (b) Let $s \in U(0,1)$ be a random number from the uniform distribution on $[0,1]$.

   (c) If $s \leq \Delta t / \mathrm{T}_{\mathrm{per}}$ or if $\Delta t < \mathrm{T}_{\mathrm{per}}$ (where $\mathrm{T}_{\mathrm{per}}$ is the cell's mean persistence time), then continue. Otherwise, leave $\mathbf{v}_{\mathrm{mot}}$ unchanged and exit.

   (d) Choose a random direction $\boldsymbol{\xi}$ in 3-D:

      i. Choose $\theta \in U(0, 2\pi)$ to be a random angle in $[0, 2\pi]$.

      ii. Choose $\phi \in U(0, \pi)$ to be a random angle in $[0, \pi]$. If `restrict_to_2D == true`, set $\phi = \frac{\pi}{2}$.

      iii. Set $\boldsymbol{\xi} = [\sin(\phi)\cos(\theta), \sin(\phi)\sin(\theta), \cos(\phi)]$.

   (e) Update the motility bias vector $\mathbf{b}$ and the bias b by calling `functions.update_migration_bias`.

   (f) Set $\mathbf{v}_{\mathrm{mot}}$ according to biased random motion:

   $$\mathbf{v}_{\mathrm{mot}} = s_{\mathrm{mot}} \frac{(1-b)\boldsymbol{\xi} + b\mathbf{b}}{||(1-b)\boldsymbol{\xi} + b\mathbf{b}||} \tag{1}$$

   Here $0 \leq b \leq 1$ is the motility bias (`phenotype.motility.migration_bias`), $\mathbf{b}$ is the migration bias direction (`phenotype.motility.migration_bias_direction`), and s_{mot} is the migration speed (`phenotype.motility.migration_speed`). See Section 11.6.

See Section 9.4.3 for more information on cell functions, and Section 11.6 for further details on cell motility.

2. **void advance_bundled_phenotype_functions(double dt_)** automatically runs the following cell functions once every phenotype time step (by default, once per 6 simulated minutes):

   (a) Evaluate `functions.update_phenotype` to update the phenotype based upon the current sampling of the microenvironment and any other parameters (e.g., cell-cell interactions). If `update_phenotype == NULL`, this is skipped. See Section 10 for more information on the cell phenotype class.

   (b) Advance the cell volume model by `dt_` time by calling `functions.volume_update_function`. Skip this step if `functions.volume_update_function == NULL`. See Section 11.3 for more information on cell volume.

   (c) Update the cell geometry (radius, nuclear radius, surface area, etc.) by calling `phenotype.geometry.update`. See Section 11.4 for more information on the cell geometry.

   (d) Check for death events in the next `dt_` time (such as apoptosis and necrosis) by calling `phenotype.death.check_for_death`. If the function returns `true`, set the cell's current cycle model to the appropriate death model, evaluate any death entry functions, and set motility to zero. Cell secretions are set to zero, and cell uptake is cut by a factor of 10. (Users can make further changes to secretion and uptake using `entry_functions` that are called at the start of a death model phase. See Section 11.1.1.) See Section 11.2 for more information on cell death, and see Section 11.1 on the general cycle class.

   (e) Advance the current cycle model, whether it is a (live) cell cycle model or a death cycle model, by calling `phenotype.cycle.advance_cycle`. See Section 11.1 for more details.

   (f) If `phenotype.flagged_for_removal == true`, call `flag_for_removal()`.
   If `phenotype.flagged_for_division == true`, call `flag_for_division()`.

   See Section 9.4.3 for more details on these cell functions, and Section 17 for examples.

3. **void add_potentials(Cell*)** is used in the mechanics functions. Users should almost never call this function.

4. **void set_previous_velocity(double xV, double yV, double zV)** sets the cell's previous velocity (for use in Adams-Bashforth evolution of the cell position) to `[xV,yV,zV]`. Users are not expected to ever need to call this function.

5. **int get_current_mechanics_voxel_index()** returns the index of the `Container` currently containing the cell.

6. **void turn_off_reactions(double)** turns off all secretions and uptake by the cell.

7. **void flag_for_division(void)** adds the cell to the list of cells that should divide at the next opportunity.

8. **void flag_for_removal(void)** adds the cell to the list of cells that should be removed from the simulation at the next opportunity.

9. **void start_death(int death_model_index)** immediately kills the cell (with the death model indicated by `death_model_index`), sets `cell.phenotype.death.dead = true`, sets motility to zero, sets secretion to zero, cuts all uptake by a factor of ten, and executes any "entry" functions for the associated death model.

10. **Cell* divide(void)** performs cell division, and returns the memory address of the newly-created cell.

11. **void die(void)** removes the cell from the simulation.

12. **void step(double dt)** is neither used nor implemented. It will be deprecated.

13. **Cell()** is the default constructor. Users should use the `create_cell` functions rather than this constructor. See Section 9.3.

14. **bool assign_position(std::vector<double> new_position)** safely sets the cell's position to `new_position`. Always use this rather than directly editing `position` (inherited from `Basic_Agent`).

15. **bool assign_position(double, double, double)** performs the same function as above, but supplying the x, y, and z coordinates separately.

16. **void set_total_volume(double)** safely sets the cell's total volume. It also sets the cell's sub-volumes proportionally.

17. **double& get_total_volume(void)** returns the cell's current total volume, stored in `cell.phenotype.volume.total`. It is *not* the preferred way to access this data, but it is provided to overwrite the base `Basic_Agent::get_total_volume` function.

18. **void update_position(double dt)** uses the cell's current and previous velocities to safely update the cell's position, using the second-order Adams-Bashforth algorithm.

19. **void assign_orientation()** sets the cell's `state.orientation` according to its current model (if any). See Section 9.4.3.

20. **void copy_function_pointers(Cell*)** overwrites the functions in `functions` with those from the supplied `Cell`.

21. **void update_voxel_in_container(void)** updates the cell's position within the interaction testing data structure.

22. **void copy_data(Cell *)** copies the member data (including `custom_data`, `parameters`, but *not* `phenotype` from the Cell at `Cell*`.

23. **void set_phenotype(Phenotype& phenotype)** is no longer used and will be deprecated. Users can now safely overwrite the cell's phenotype at any time.

24. **void update_radius()** is neither implemented nor used. It will be deprecated.

25. **Cell_Container* get_container()** returns the memory address of the `Cell_Container` containing the cell.

26. **std::vector<Cell*>& cells_in_my_container(void)** returns (by reference) a vector of pointers to the cells in the current mechanics voxel. ***For performance reasons, we are giving users direct access to the underlying data structures, rather than copies. It is critical that users do not alter the underlying array of `Cell*`.*** Future releases of PhysiCell will provide more refined access functions, including better lists of neighbors and lists of nearby mechanics voxels.

27. **void convert_to_cell_definition(Cell_Definition& cd)** converts the cell to match the name, type, custom data, parameters, function, and phenotype stored in the Cell Definition in `cd`. (See Section 9.4.5 for more information on cell definitions.) As of Version 1.2.2, this function does not overwrite the `is_movable`, `is_out_of_domain`, and `displacement` data of the cell.

    This function is particularly useful if you want to do thinks like stem cell heirarchies, where you might "convert" a stem cell to a differentiated cell, each of which has been defined separately.

[Return to Table of Contents.]

9.3 Other key functions

The following are functions in the `PhysiCell` namespace (and are not members of the `Cell` class).

1. **Cell* create_cell(void)** creates a new cell (with default `Cell_Definition`; see Section 9.4.5) at (0,0,0) and registers it in all the relevant data structures. This is the *safe* way to create a new cell in the simulation.

    Example: Let's create a cell (with the default `Cell_Definition`) at (15,27,-32):

```
Cell* pC = create_cell();
pC->assign_position(15,27,-32);
```

2. **Cell* create_cell(Cell_Definition& cd)** creates a new cell at (0,0,0) with parameters, phenotype, functions, and other properties specified in `cd` (See Section 9.4.5), and registers it in all the relevant data structures. This is the *safe* way to create a new cell in the simulation.

    Example: Let's define a new type of cell (`stem_cell`), then create a new cell of this type at (15,27,-32):

```
// create a new cell definition
Cell_Definition stem_cell;

// Operations to set the properties and models
// of the stem_cell type go here.

// create a new stem_cell, and move it.
Cell* pC = create_cell(stem_cell);
pC->assign_position(15,27,-32);
```

3. **void delete_cell(int)** deletes the cell located at `all_cells(int)` and removes it from all relevant data structures. This is the *safe* way to directly remove a cell from a simulation.

    Example: Let's check the 13th cell and delete it if a custom variable "infected" (See Section 9.4.1) indicates that it is infected. (See Section 15.4 for more on the vector `all_cells` that lists the memory addresses of all cells.)

```
Cell* pCell = (*all_cells)[13];

if( pCell->custom_data[ "infected" ] == true )
{
    delete_cell( 13 );
}
```

4. **`void delete_cell( Cell* )`** deletes the cell with located at `Cell*` and removes it from all relevant data structures. This is the *safe* way to directly remove a cell from a simulation.

   Example: Let's check the 13th cell and delete it if a custom variable "infected" (See Section 9.4.1) indicates that it is infected. (See Section 15.4 for more on the vector `all_cells` that lists the memory addresses of all cells.)

```
Cell* pCell = (*all_cells)[13];

if( pCell->custom_data[ "infected" ] == true )
{
    delete_cell( pCell );
}
```

[Return to Table of Contents.]

9.4 Important classes (except Phenotype)

9.4.1 Custom_Cell_Data

This class allows users to dynamically add new custom data to individual cells, or to a `Cell_Definition`. (See Section 9.4.5.) Here is the full class definition:

```
class Custom_Cell_Data
{
 private:
    std::unordered_map<std::string,int> name_to_index_map;
    friend std::ostream& operator<<(std::ostream& os, const Custom_Cell_Data& ccd);
 public:
    std::vector<Variable> variables;
    std::vector<Vector_Variable> vector_variables;

    int add_variable( Variable& v );
    int add_variable( std::string name , std::string units , double value );
    int add_variable( std::string name , double value );

    int add_vector_variable( Vector_Variable& v );
    int add_vector_variable( std::string name ,
        std::string units , std::vector<double>& value );
    int add_vector_variable( std::string name , std::vector<double>& value );

    int find_variable_index( std::string name );

    double& operator[]( int i );
    double& operator[]( std::string name );

    Custom_Cell_Data();
    Custom_Cell_Data( const Custom_Cell_Data& ccd );
};
```

Here are the main data elements and member functions, in greater detail:

1. **std::vector<Variable> variables** is a vector of variables (initially empty). A `Variable` has the following form:

```
class Variable
{
 private:
    friend std::ostream& operator<<(std::ostream& os, const Variable& v);
 public:
    std::string name;
    double value;
    std::string units;

    Variable();
};
```

2. **std::vector<Vector_Variable> vector_variables** is a vector of vector variables (initially empty). A `Vector_Variable` has the following form:

```
class Vector_Variable
{
 private:
    friend std::ostream& operator<<(std::ostream& os, const Vector_Variable& v);

 public:
    std::string name;
    std::vector<double> value;
    std::string units;

    Vector_Variable();
};
```

3. **int add_variable(Variable& v)** adds the new variable `v` to the custom data, and returns the index of the newly added variable.

   Example:

```
Custom_Cell_Data ccd;
Variable v;

v.name = "sensitivity";
v.value = 0.3;
v.units = "dimensionless";

ccc.add_variable( v );
```

4. **int add_variable(std::string name , std::string units , double value)** adds a new variable with name `name`, units `units`, and value `value`. It returns the index of the newly added variable.

   Example:

   ```
   Custom_Cell_Data ccd;
   ccd.add_variable( "sensitivity", "dimensionless", 0.3 );
   ```

5. **int add_variable(std::string name , double value)** adds a new variable with name `name`, unspecified units, and value `value`. It returns the index of the newly added variable.

   Example:

   ```
   Custom_Cell_Data ccd;
   ccd.add_variable( "sensitivity", 0.3 );
   ```

6. **int add_vector_variable(Vector_Variable& v)** adds the new vector variable `v` to the custom data, and returns the index of the newly added variable.

   Example:

   ```
   Custom_Cell_Data ccd;
   Vector_Variable v;

   v.name = "home";
   v.value = {0,1,2};
   v.units = "micron";

   ccc.add_vector_variable( v );
   ```

7. **int add_vector_variable(std::string name, std::string units,
   std::vector<double>& value)** adds a new vector variable with name `name`, units `units`, and (vector) value `value`. It returns the index of the newly added variable.

   Example:

   ```
   Custom_Cell_Data ccd;

   std::vector<double> myvec = {0,1,2};

   ccc.add_vector_variable( "home", "micron", myvec );
   ```

8. **int add_vector_variable(std::string name , std::vector<double>& value)** adds a new vector variable with name `name`, unspecified units, and (vector) value `value`. It returns the index of the newly added variable.

   Example:

   ```
   Custom_Cell_Data ccd;

   std::vector<double> myvec = {0,1,2};

   ccc.add_vector_variable( "home", myvec );
   ```

9. **int find_variable_index(std::string name)** returns the index of the variable with name `name`, if it exists. Note that this function is case sensitive.

   Example:

```
Custom_Cell_Data ccd;

ccd.add_variable( "oxygen" , "mmHg", 38.0 );
ccd.add_variable( "maple syrup" , "megayums", 1.2 );
ccd.add_variable( "VEGF", "dimensionless", 0.01 );

int syrup_index = ccd.find_variable_index( "maple syrup" );
std::cout << ccd.variables[syrup_index].value << std::endl;
```

10. **double& operator[](int i)** access the ith scalar variable.

   Example:

```
Custom_Cell_Data ccd;

ccd.add_variable( "oxygen" , "mmHg", 38.0 );
ccd.add_variable( "maple syrup" , "megayums", 1.2 );
ccd.add_variable( "VEGF", "dimensionless", 0.01 );

int syrup_index = ccd.find_variable_index( "maple syrup" );
std::cout << ccd.variables[syrup_index].value << std::endl;
std::cout << ccd[syrup_index] << std::endl;
```

11. **double& operator[](std::string name)** access the scalar variable with name `name`. Note that this function is case sensitive.

   Example:

```
Custom_Cell_Data ccd;

ccd.add_variable( "oxygen" , "mmHg", 38.0 );
ccd.add_variable( "maple syrup" , "megayums", 1.2 );
ccd.add_variable( "VEGF", "dimensionless", 0.01 );

int syrup_index = ccd.find_variable_index( "maple syrup" );
std::cout << ccd.variables[syrup_index].value << std::endl;
std::cout << ccd[syrup_index] << std::endl;
std::cout << ccd["maple syrup"] << std::endl;
```

12. **Custom_Cell_Data()** is the default constructor. It creates a custom cell data structure with no variables and no vector variables.

13. **Custom_Cell_Data(const Custom_Cell_Data& ccd)** is the copy constructor, where the new `Custom_Cell_Data` is initialized with all contents equal to `ccd`.

14. **Streaming:** You can display a custom variable to any `C++` streaming operator.

    Example: We'll create a custom cell data structure called `my_custom_data`, add some scalar and vector variables, and display it.

```
my_custom_data.add_variable( "spring constant", "1/min" , 0.01 );
my_custom_data.add_variable( "relaxation rate", "1/min" , 7e-5 );

my_custom_data.add_variable( "strain", "micron" , 0.0 );
my_custom_data.add_variable( "integrated strain", "micron*min" , 0.0 );

my_custom_data.add_variable( "max strain", "micron" , 10.0 );
my_custom_data.add_variable( "max integrated strain", "micron*min" , 600.0 );

my_custom_data.add_vector_variable( "home" , "micron" , {0,0,0} );

std::cout << my_custom_data << std::endl;
```

    The output should look like this:

```
Custom data (scalar):
0: spring constant: 0.01 1/min
1: relaxation rate: 7.0e-005 1/min
2: strain: 0 micron
3: integrated strain: 0 micron*min
4: max strain: 10 micron
5: max integrated strain: 600 micron*min
Custom data (vector):
0: home: [0,0,0] micron
```

[Return to Table of Contents.]

9.4.2 Cell_Parameters

This class stores standardized parameters for a `Cell` (or a `Cell_Definition`; see Section 9.4.5). In future editions of PhysiCell, some of these may be moved into the phenotype. (See Section 10.) Here is the full class definition:

```
class Cell_Parameters
{
 private:
 public:
    double o2_hypoxic_threshold;
    double o2_hypoxic_response;
    double o2_hypoxic_saturation;

    double o2_proliferation_saturation;
    double o2_proliferation_threshold;
```

```
    double o2_reference;

    double o2_necrosis_threshold;
    double o2_necrosis_max;

    Phenotype* pReference_live_phenotype;

    double max_necrosis_rate;
    int necrosis_type;

    Cell_Parameters();
}
```

Here are the main data elements and member functions, in greater detail:

1. **double o2_hypoxic_threshold** is the oxygen value (in mmHg) below which hypoxic signaling starts. (default: 15 mmHg, or about 2% oxygen).

   $$\texttt{o2\_hypoxic\_saturation} \le \texttt{o2\_hypoxic\_response} \le \texttt{o2\_hypoxic\_threshold}$$

2. **double o2_hypoxic_response** is the oxygen value (in mmHg) below which hypoxic responses are observed (e.g., omics changes). (default: 8 mmHg)

   $$\texttt{o2\_hypoxic\_saturation} \le \texttt{o2\_hypoxic\_response} \le \texttt{o2\_hypoxic\_threshold}$$

3. **double o2_hypoxic_saturation** is the oxygen value (in mmHg) below which hypoxic responses are at a maximum. (default: 4 mmHg)

   $$\texttt{o2\_hypoxic\_saturation} \le \texttt{o2\_hypoxic\_response} \le \texttt{o2\_hypoxic\_threshold}$$

4. **double o2_proliferation_saturation** is the oxygen value (in mmHg) above which the proliferation rate is maximmized. No further oxygenation benefits the cell. (default: 160 mmHg, or 21% standard culture conditions)

   $$\texttt{o2\_proliferation\_threshold} \le \texttt{o2\_reference} \le \texttt{o2\_proliferation\_saturation}$$

5. **double o2_proliferation_threshold** is the oxygen value (in mmHg) below which the proliferation ceases. (default: 5 mmHg)

   $$\texttt{o2\_proliferation\_threshold} \le \texttt{o2\_reference} \le \texttt{o2\_proliferation\_saturation}$$

6. **double o2_reference** is the oxygen value that corresponds to the reference phenotype (see below). (default: 160 mmHg)

   $$\texttt{o2\_proliferation\_threshold} \le \texttt{o2\_reference} \le \texttt{o2\_proliferation\_saturation}$$

7. **double o2_necrosis_threshold** is the oxygen value at which necrosis starts. (default: 5 mmHg)

   o2_necrosis_max ≤ o2_necrosis_threshold

8. **double o2_necrosis_max** is the oxygen value at which necrosis reaches its maximum rate. (default: 2.5 mmHg)

   o2_necrosis_max ≤ o2_necrosis_threshold

9. **Phenotype* pReference_live_phenotype** is a pointer to a Phenotype (See Section 10) which will serve as reference values when oxygen is equal to o2_reference.

10. **double max_necrosis_rate** is the necrosis rate (in units 1/min) when the oxygen value dips below o2_necrosis_max.

11. **int necrosis_type** is used to decide between deterministic and stochastic necrosis. (Use PhysiCell_constants::deterministic_necrosis or PhysiCell_constants::stochastic_necrosis.)

12. **Cell_Parameters()** is the default constructor.

[Return to Table of Contents.]

9.4.3 Cell_Functions

This data structure attaches user-specified functions for key cell behaviors. Note that in PhysiCell, almost all functions that act on cells take the form

```
void function( Cell* pCell, Phenotype& phenotype, double dt ).
```

Ordinarily, phenotype = pCell->phenotype, but we allow these to be specified separately for cases where pCell = NULL.

Here is the full class definition:

```
class Cell_Functions
{
 private:
 public:
   Cycle_Model cycle_model;

   void (*volume_update_function)( Cell* pCell, Phenotype& phenotype , double dt );
   void (*update_migration_bias)( Cell* pCell,
       Phenotype& phenotype, double dt );

   void (*custom_cell_rule)( Cell* pCell, Phenotype& phenotype, double dt );
   void (*update_phenotype)( Cell* pCell, Phenotype& phenotype, double dt );
```

```
    void (*update_velocity)( Cell* pCell, Phenotype& phenotype, double dt );

    void (*add_cell_basement_membrane_interactions)(Cell* pCell,
        Phenotype& phenotype, double dt );
    double (*calculate_distance_to_membrane)( Cell* pCell,
        Phenotype& phenotype, double dt );

    void (*set_orientation)(Cell* pCell, Phenotype& phenotype, double dt );

    Cell_Functions();
};
```

Here are the member data and functions in greater detail:

1. **`Cycle_Model cycle_model`** is the cell cycle model to be used for this cell (or `Cell_Definition`–see Section 9.4.5). The `Cycle\_Model` class is discussed in greater detail in Section 11.1.3.

2. **`void (*volume_update_function)( Cell* pCell, Phenotype& phenotype, double dt )`** is a function pointer to a user-specified model of cell volume regulation, based upon the parameters stored in `phenotype.volume`. See Section 11.3 for more detail. We recommend the reference model in [3]: `standard_volume_update_function`.

3. **`void (*update_migration_bias)( Cell* pCell, Phenotype& phenotype, double dt )`** is a function pointer to a user-specified model to set the migration bias direction, e.g., towards a chemical gradient. See Section 11.6 for more detail. We give an example in Section 17.1.2.

4. **`void (*custom_cell_rule)( Cell* pCell, Phenotype& phenotype, double dt )`** is a custom function that is executed every time the cell updates its mechanics. We give an example in Section 17.1.3

5. **`void (*update_phenotype)( Cell* pCell, Phenotype& phenotype, double dt )`** is where users should alter the cell's phenotype based upon microenvironmental conditions. We give an example in Section 17.1.4. We recommend the reference model `update_cell_and_death_parameters_O2_based` found in [3].

6. **`void (*update_velocity)( Cell* pCell, Phenotype& phenotype, double dt )`** is a pointer to a user-specified model to set the cell's velocity, based upon the mechanics model. We recommend `standard_update_cell_velocity` from [3].

7. **`void (*add_cell_basement_membrane_interactions)(Cell* pCell, Phenotype& phenotype, double dt)`** modifies the cell's velocity based upon interactions with basement membrane given in this function. This function may change in future releases.

8. **`double (*calculate_distance_to_membrane)( Cell* pCell, Phenotype& phenotype, double dt );`** calculates the (signed) distance to the basement membrane (e.g., by an analytic solution, via a level set function, or by other discrete means).

9. **`void (*set_orientation)(Cell* pCell, Phenotype& phenotype, double dt )`** is a user-supplied function to update the cell's orientation, based upon interactions wiht other neighbors or other factors.

10. **Cell_Functions()** is the default constructor.

[Return to Table of Contents.]

9.4.4 Cell_State

The Cell_State is a small collection of cell descriptors that do not neatly belong in the cell Phenotype. Here is the full class definition:

```
class Cell_State
{
 public:
    std::vector<Cell*> neighbors;
    std::vector<double> orientation;

    double simple_pressure;

    Cell_State();
};
```

And here are further details on the member data and functions:

1. **std::vector<Cell*> neighbors** is the memory addresses of all cells that are currently neighbors of the cell (as determined by the adhesion interaction potential). **This variable is not currently updated in PhysiCell, but it will be in a future release.**

2. **std::vector<double> orientation** is the cell's current basal-to-apical orientation, as a unit vector. Please note that if the cell's `phenotype.geometry.polarity = 0`, then this state variable has no meaning. Also note that in 2-D simulations, one should set `orientation = {0,0,1}`.

3. **double simple_pressure** is a simple analog for mechanical pressure, nondimensionalized by a scale. We define the dimensional pressure p_i by

$$p_i = \frac{1}{S_i} \sum_{j \in \mathcal{N}_i} \left| \mathbf{F}_{ij}^{\text{ccr}} \right| \tag{2}$$

where $\mathcal{N}_i$ is the set of the neighbor cells of the cell, $\mathbf{F}_{ij}^{\text{ccr}}$ is the cell-cell "repulsive" imparted on cell i by its neighbor j, and S_i is the cell surface area. In [3, 7], we approximated $\mathbf{F}_{ij}^{\text{ccr}}$ by

$$\mathbf{F}_{ij}^{\text{ccr}} = c_j^{\text{ccr}} \left(1 - \frac{d_{ij}}{R_i + R_j} \right)^2 \frac{\mathbf{d}_{ij}}{d_{ij}}, \tag{3}$$

where $\mathbf{d}_{ij} = \mathbf{x}_j - \mathbf{x}_i$ and $d_{ij} = |\mathbf{d}_{ij}|$.

To define a scale, suppose the cell is in close packing in 3D by equally-sized cells with total volume V, mean radius R, maximal cross-sectional area A, and equal coefficient $c_{\text{ccr}}^j = c_{\text{ccr}}^i$ for each neighbor j. In 3D, the cell has 12 neighbors in a tight sphere packing. By [4], the equilibrium cell-cell distance s in a 3-D confluent cell packing is

$$\frac{s}{2R} = \frac{1}{2R}\sqrt{\frac{2A}{\sqrt{3}}} = \sqrt{\frac{2\pi R^2}{4R^2\sqrt{3}}} = \sqrt{\frac{\pi}{2\sqrt{3}}} \approx 0.9523. \tag{4}$$

This defines a pressure scale:

$$\overline{p}_i = 12\frac{c^i_{\mathrm{ccr}}}{S_i}\left(1-\sqrt{\frac{\pi}{2\sqrt{3}}}\right)^2 \approx 0.02729\frac{c^i_{\mathrm{ccr}}}{S_i}. \tag{5}$$

Thus, we define a dimensionless analog of pressure by:

$$\begin{aligned}
\texttt{simple_pressure} = \frac{p_i}{\overline{p}_i} &= \frac{1}{\left(1-\sqrt{\frac{\pi}{2\sqrt{3}}}\right)^2}\sum_{j\in\mathcal{N}_i}\frac{c^j_{\mathrm{ccr}}}{c^i_{\mathrm{ccr}}}\left(1-\frac{d_{ij}}{R_i+R_j}\right)^2 \\
&= \frac{2\sqrt{3}}{\left(\sqrt{2\sqrt{3}}-\sqrt{\pi}\right)^2}\sum_{j\in\mathcal{N}_i}\frac{c^j_{\mathrm{ccr}}}{c^i_{\mathrm{ccr}}}\left(1-\frac{d_{ij}}{R_i+R_j}\right)^2 \\
&\approx 439.74\sum_{j\in\mathcal{N}_i}\frac{c^j_{\mathrm{ccr}}}{c^i_{\mathrm{ccr}}}\left(1-\frac{d_{ij}}{R_i+R_j}\right)^2 \\
&= \frac{439.74}{c^i_{\mathrm{ccr}}}\sum_{j\in\mathcal{N}_i}c^j_{\mathrm{ccr}}\left(1-\frac{d_{ij}}{R_i+R_j}\right)^2. \qquad (6)
\end{aligned}$$

4. **`Cell_State()`** is the default constructor.

9.4.5 Cell Definition

The `Cell_Definition` class allows users to define a cell type, including its default phenotype, and all its custom functions. Users can then work on defining multiple cell types at the start of a simulation and using these to initialize many cells at the start of a simulation (or during a simulation).

Here is the full class definition:

```
class Cell_Definition
{
 private:
 public:
    int type;
    std::string name;

    Microenvironment* pMicroenvironment;

    Cell_Parameters parameters;
    Custom_Cell_Data custom_data;
    Cell_Functions functions;
    Phenotype phenotype;

    Cell_Definition();
};
```

Here are the member data and functions in greater detail:

1. **int type** is a unique identifier for the cell type. It will be copied to the cell's type.

2. **std::string name** is the "plain English" name of the cell type. It is copied to the cell's type_name.

3. **pMicroenvironment** is a pointer to the simulation's BioFVM microenvironment, which is always &microenvironment.

4. **parameters** is defined in Section 9.4.2. It is copied to the cell's parameters.

5. **custom_data** is defined in Section 9.4.1. It is copied to the cell's custom_data.

6. **functions** is defined in Section 9.4.3. It is copied to the cell's functions.

7. **phenotype** is defined in Section 10. It is copied to the cell's phenotype.

8. **Cell_Definition()** is the default constructor.

[Return to Table of Contents.]

---

10 Phenotype

The Phenotype is one of the most important components of a cell in PhysiCell, allowing us to specify the current state and properties of a cell. Most functions in PhysiCell are based upon either updating the phenotype or advancing the simulation based upon each cell's current phenotype.

The Phenotype is divided into multiple functional groups:

1. **Cycle** gives cell cycle information, including the graph structure of the cycle model, associated parameter values (transition rates), and information on the current cycle phase. See Section 11.1. Note that for a dead cell, the "cycle model" is set to a death model.

2. **Death** gives current cell death rates for one or more death models. See Section 11.2.

3. **Volume** gives the cell's current total volume, as well as nuclear, cytoplasmic, fluid, and other subvolumes. See Section 11.3.

4. **Geometry** records the cell's radius, nuclear radius, and surface area (using spherical approximations). See Section 11.4.

5. **Mechanics** records the cell's adhesion and repulsion strength parameters for interactions with cells and the basement membrane, as well as the maximum adhesive interaction distance (a multiple of the cell's equivalent radius). Future releases of PhysiCell will likely expand this aspect of the cell phenotype. See Section 11.5.

6. **Motility** records phenotype information on motility, currently including persistence, a direction for biased random walks (e.g., chemotaxis), and a scalar bias parameter that can vary motility between purely Brownian and completely deterministic along the current bias direction. See Section 11.6.

7. **Secretion** records cell secretion and uptake rates, as well as a saturation density (at which secretion ends). See Section 11.7.

Here is how the Phenotype class is defined in PhysiCell_phenotype.h:

```
class Phenotype
{
 private:
 public:
    bool flagged_for_division;
    bool flagged_for_removal;

    Cycle cycle;
    Death death;
    Volume volume;
    Geometry geometry;
    Mechanics mechanics;
    Motility motility;
    Secretion secretion;

    Phenotype();

    void sync_to_functions( Cell_Functions& functions );

    void sync_to_default_functions( void );
};
```

In the Section 11, we give further details on the data elements within the `Phenotype` class.

[Return to Table of Contents.]

10.1 Member functions

1. **`Phenotype()`** is the default constructor.

2. **`void sync_to_functions( Cell_Functions& functions )`** makes sure that `phenotype.cycle` is matched to the cycle model and parameters in `functions.cycle_model`. See Section 11.1 for more details on the cycle model.

3. **`void sync_to_default_functions( void )`** is neither implemented nor used, because default constructors take care of this. The function will be deprecated.

[Return to Table of Contents.]

11 Phenotype details

11.1 Cycle Models

`Cycle` stores the graph structure and parameters of the current cycle model, which can either be a cell cycle model or a death cycle model. It also includes member functions to progress through the cycle, and call appropriate cell division or removal functions. Before introducing `Cycle` (Section 11.1.5), we will detail the sub-classes necessary to work with a cycle model. All these data structures have been introduced

with the goal of separating the graph structure of a cycle model (`Cycle_Model`: Section 11.1.3) from its parameter values (`Cycle_Data`: section 11.1.4), while still bundling them in a simple structure (`Cycle`: Section 11.1.5) for use in a `Phenotype`.

A `Cycle_Model` is constructed of one or more `Phases` (Section 11.1.1) that are connected into a directed graph by `Phase_Links` (Section 11.1.2).

[Return to Table of Contents.]

11.1.1 Phase

The `Phase` class defines the phase in a cycle model, including the following data elements:

1. **`int index`** is an internal unique index within a cycle model.
2. **`int code`** is a unique global identifier for the phase, using constants defined in `PhysiCell_constants.h` using `PhysiCell_constants`. See Section 15.7.
3. **`std::string name`** is the "plain English" name of the phase (e.g., G0/G1).
4. **`bool division_at_phase_exit`** is a Boolean variable that indicates whether the cell should divide at the end of this phase (e.g., at the end of M phase).
5. **`bool removal_at_phase_exit`** is a Boolean variable that indicates whether the cell should be removed at the end of this phase (e.g., at the end of apoptosis).
6. **`void (*entry_function)(Cell* pCell, Phenotype& phenotype, double dt)`** is a pointer to a function that is executed at the start of the phase. This would be a good place to write a function to perform mutations (e.g., at the start of G0/G1, randomly choose selected phenotype parameters according to a distribution).

Here is the formal class declaration:

```
class Phase
{
 public:
     int index;
     int code;
     std::string name;

     bool division_at_phase_exit;
     bool removal_at_phase_exit;

     void (*entry_function)( Cell* pCell, Phenotype& phenotype, double dt );

     Phase();
};
```

Member functions

1. **Phase()** is the default constructor. It sets `index = 0`, `code = 0`, `name = "unnamed"`, all flags to `false`, and the entry function to `NULL`.

[Return to Table of Contents.]

11.1.2 Phase_Link

The `Phase_Link` class is a link between one `Phase` and another (e.g., progression from G0/G1 to S phase). It includes the following data elements:

1. **int start_phase_index** is the unique index of the starting phase in the phase transition (a link from phase `start_phase_index` to phase `end_phase_index`).

2. **int end_phase_index** is the unique index of the ending phase in the phase transition (a link from phase `start_phase_index` to phase `end_phase_index`).

3. **bool fixed_duration** is a Boolean variable that indicates whether the cell spends a fixed amount of time before transitioning from phase `start_phase_index` to phase `end_phase_index`. Note that this variable only makes sense if there is a single, unique phase transition starting from `start_phase_index`.

4. **void (*arrest_function)(Cell* pCell, Phenotype& phenotype, double dt)** is a function pointer that allows you to set an arrest condition for the phase transition (e.g., only progress from M to G0/G1 if the cell is of sufficient volume). Its return value is `true` if the transition is arrested, and `false` if the transition is allowed to proceed. Set this pointer to `NULL` to bypass checking for arrest in this phase link.

5. **void (*exit_function)(Cell* pCell, Phenotype& phenotype, double dt)** is a function pointer to a function that is executed at the end of the phase transition. This would be a good place to write a function to perform mutations (e.g., at the transition from M to G0/G1, randomly choose selected phenotype parameters according to a distribution).

Here is the formal class declaration:

```
class Phase_Link
{
 public:
     int start_phase_index;
     int end_phase_index;

     bool fixed_duration;

     bool (*arrest_function)( Cell* pCell, Phenotype& phenotype, double dt );
     void (*exit_function)(  Cell* pCell, Phenotype& phenotype, double dt );

     Phase_Link();
};
```

Member functions

1. **`Phase_Link()`** is the default constructor. It sets all indices to 0, all flags to `false`, and it sets `arrest_function = NULL`.

[Return to Table of Contents.]

11.1.3 Cycle_Model

The `Cycle_Model` connects one or more `Phase`s through `Phase_Link`s, and stores this entire graph structure along with parameter values. (See `Cycle_Data` in Section 11.1.4.) It also contains member functions to assist with constructing and executing a cycle model, as well as to readily access transition rates. Here are the key data elements:

1. **`std::vector< std::unordered_map<int,int> > inverse_index_maps`** is an internal (private) data structure for easily accessing specific phase transitions. For reference, `index_inverse_map[i][j] = k` helps us find the `Phase_Link` from `Phase` i to `Phase` j, which is stored in `phase_links[i][k]`, and whose transition rate is stored in `data.transition_rates[i][k]`.

2. **`std::string name`** is the "plain English" name of the cycle model (e.g., Ki67 (Basic)).

3. **`int code`** is a unique global (integer) identifier for the cycle model, using constants defined in `PhysiCell_constants.h` using `PhysiCell_constants`. (See Section 15.7.)

4. **`std::vector<Phases> phases`** is a vector of Phases.

5. **`std::vector< std::vector<Phase_Link> > phase_links`** is a vector of vectors of `Phase_Links`. Note that `phase_links[i]` is the vector of all phase links starting at phase `i`. (This is why we need the inverse map above.)

6. **`int default_phase_index`** is the index of the default phase in the cycle model (usually 0). If a new `Phenotype` instance is created and assigned this cycle model, it should start out in this phase.

7. **`Cycle_Data data`** (of type `Cycle_Data`) contains a template of the key parameter values for this cycle model. Place default values for your cycle model here, as a copy of `data` is passed to any new `Phenotype` instance containing the cycle model. See Section 11.1.4.

Here is the formal class declaration:

```
class Cycle_Model
{
 private:
     std::vector< std::unordered_map<int,int> > inverse_index_maps;

 public:
     std::string name;
     int code;
```

```
    std::vector<Phase> phases;
    std::vector< std::vector<Phase_Link> > phase_links;

    int default_phase_index;

    Cycle_Data data;

    Cycle_Model();

    void advance_model( Cell* pCell, Phenotype& phenotype, double dt );

    int add_phase( int code, std::string name );

    int add_phase_link( int start_index, int end_index ,
         bool (*arrest_function)( Cell* pCell, Phenotype& phenotype, double dt ) );
    int add_phase_link( int start_index, int end_index , double rate ,
         bool (*arrest_function)( Cell* pCell, Phenotype& phenotype, double dt ) );

    int find_phase_index( int code );
    int find_phase_index( std::string );

    double& transition_rate( int start_index , int end_index );
    Phase_Link& phase_link(int i,int j);

    std::ostream& display( std::ostream& os );
};
```

Member functions

1. **`Cycle_Model()`** is the default constructor. It sets `name = "unnamed"`, sets `code = PhysiCell_constants::custom_cycle_model`, sets the model to an empty one with no phases, and the links `data.pCycle_Model` to `this` (the `Cycle_Model` under construction). See Section 11.1.4.

2. **`int add_phase(int code, std::string name)`** adds a new `Phase` to the cycle model, with the supplied `code` and `name`. It returns the index (`new_index`) of the new phase, so that you can subsequently access it with `phases[new_index]`.

3. **`int add_phase_link( int start_index, int end_index,`**
   `bool (*arrest_function)( Cell* pCell, Phenotype& phenotype, double dt ) )`
   adds a new `Phase_Link` to `phase_links[start_index]` joining phase `start_index` to phase `end_index`, with the arrest function `arrest_function`. It resizes internal data structures including `data` (and its `transition_rates`) automatically, and sets the transition rate to 0.0. It returns the index (`new_index`) so that the phase link can be directly accessed as `phase_links[start_index][new_index]`.

4. **`int add_phase_link( int start_index, int end_index, double rate`**
   `bool (*arrest_function)( Cell* pCell, Phenotype& phenotype, double dt ) )`
   adds a new `Phase_Link` to `phase_links[start_index]` joining phase `start_index` to phase `end_index`, with the arrest function `arrest_function`. It resizes internal data structures including `data` (and

its `transition_rates`) automatically, and sets the transition rate to `rate`. It returns the index (`new_index`) so that the phase link can be directly accessed as `phase_links[start_index][new_index]`.

5. **`int find_phase_index( int code )`** finds the index `i` such that `phases[i].code = code`. Please note that this returns 0 if there is no exact match!

6. **`int find_phase_index( std::string name )`** finds the index `i` such that `phases[i].name = name`. Please note that this returns 0 if there is no exact match! Note that this function is case sensitive.

7. **`double& transition_rate( int start_index , int end_index )`** is a user-friendly interface function to access (by reference) the transition rate from phase `start_index` to phase `end_index`.

8. **`Phase_Link& phase_link(int start_index , int end_index)`** is a user-friendly interface function to access (by reference) the `Phase_Link` from phase `start_index` to phase `end_index`.

9. **`void advance_model( Cell* pCell, Phenotype& phenotype, double dt )`** advances the the cycle model by `dt` time (assumed minutes in current PhysiCell versions). For the current phase in `phenotype.cycle.data.current_phase()`, it evaluates the probability of advancing to all linked phases within `dt` time (see [3]) and changes the model (and the state of `phenotype.cycle.data`) accordingly. It will call cell division and removal functions as needed.

10. **`std::ostream& display( std::ostream& os )`** allows streaming of a basic visual output of the cycle model. I recommend calling `display( std::cout )`.

[Return to Table of Contents.]

11.1.4 Cycle_Data

The `Cycle_Data` class contains key parameters and state variables for a cell cycle model (see Section 11.1.3), as well as member functions to easily access transition rates and the current cycle phase. It includes the following data elements:

1. **`std::vector< std::unordered_map<int,int> > inverse_index_maps`** is an internal (private) data structure for easily accessing specific phase transitions. For reference, `index_inverse_map[i][j] = k` helps us find the `Phase_Link` from `Phase` i to `Phase` j in the `pCycle_Model`, which is stored in `pCycle_Model->phase_links[i][k]`, and whose transition rate is stored in `transition_rates[i][k]`.

2. **`Cycle_Model* pCycle_Model`** is a pointer to the appropriate `Cycle_Model`.

3. **`std::string time_units`** is the units of time for the cycle model. (PhysiCell currently assumes all time units are in minutes.)

4. **`std::vector< std::vector<double> > transition_rates`** is a vector of vectors of cell phase transition rates. For each `i`, `transition_rates[i]` is the vector of transition rates from Phase `i` to any other linked phases. For safety, we might make this data element private in the future, as it can be more intuitively accessed via `double& transition_rate(int,int)` (below).

5. **`int current_phase_index`** indicates the current phase in the `Cycle_Model`.

6. **`double elapsed_time_in_phase`** records how long the cell has been in the current phase.

Here is the formal class declaration:

```
class Cycle_Data
{
 private:
    std::vector< std::unordered_map<int,int> > inverse_index_maps;

 public:
    Cycle_Model* pCycle_Model;

    std::string time_units;

    std::vector< std::vector<double> > transition_rates;

    int current_phase_index;
    double elapsed_time_in_phase;

    Cycle_Data();

    Phase& current_phase( void );

    void sync_to_cycle_model( void );

    double& transition_rate(int start_phase_index, int end_phase_index );
    double& exit_rate(int phase_index );
};
```

Member functions

1. **`Cycle_Data()`** is the default constructor. It resizes `transition_rates` to zero, sets `pCycle_Model = NULL`, and defaults `time_units = "min"`.

2. **`Phase& current_phase( void )`** is a user-friendly interface function to access (by reference) the current `Phase` in the cycle. Use this to get the name and other structural information.

3. **`void sync_to_cycle_model( void )`** resizes the internal data structures for consistency with the `pCycle_Model`, if it is non-`NULL`.

4. **`double& transition_rate( int start_phase_index , int end_phase_index )`** is a user-friendly interface function to access (by reference) the transition rate from phase `start_phase_index` to phase `end_phase_index`.

5. **`double& exit_rate( int phase_index )`** is a user-friendly interface function to access (by reference) the rate of exiting the `phase_index` phase, in the case where there is only one `Phase_Link` from that phase to another. (e.g., for a cycle model where the S phase only links to the G2 phase.) In this case, the cell spends (in the mean)

$$\frac{1}{\texttt{exit\_rate}(\texttt{phase\_index})} \tag{7}$$

time in phase `phase_index`.

11.1.5 Cycle

The Cycle bundles a Cycle_Model with Cycle_Data for simpler inclusion in a Phenotype. Its main data elements are:

1. **Cycle_Model* pCycle_Model** is a pointer to a Cycle_Model.
2. **Cycle_Data data** is Cycle_Data associated with the Cycle_Model. Note that this is an *independent copy* of the pCycle_Model->data, so that a single cell's phenotype can be updated without modifying the defaults for the underlying cycle model.

Here is the formal class declaration:

```
class Cycle
{
 private:
 public:
    Cycle_Model* pCycle_Model;
    Cycle_Data data;

    Cycle();

    void advance_cycle( Cell* pCell, Phenotype& phenotype, double dt );

    Cycle_Model& model( void );
    Phase& current_phase( void );
    int& current_phase_index( void );

    void sync_to_cycle_model( Cycle_Model& cm );
};
```

Member functions

1. **Cycle()** is the default constructor. It sets pCycle_Model = NULL and uses the default constructor for the Cycle_Data.
2. **void advance_cycle(Cell* pCell, Phenotype& phenotype, double dt** advances the cycle model by dt time, executing any arrest, entry, or other functions according to the supplied phenotype. (It is implemented by calling pCycle_Model->advance_model(pCell,phenotype,dt).
3. **Cycle_Model& model(void)** returns (by reference) the cycle model pointed to by pCycle_Model.
4. **Phase& current_phase(void)** returns (by reference) the current phase (as given in data) in the cycle model.

5. **int& current_phase_index(void)** returns (by reference) the current phase index (as given in data) in the cycle model.

6. **void sync_to_cycle_model(Cycle_Model& cm)** sets pCycle_Model = &cm and then overwrites data with cm.data.

[Return to Table of Contents.]

11.2 Death models

A death model is a Cycle_Model (Section 11.1.3), where one of the phases is marked to trigger cell removal. We define Death_Parameters (Section 11.2.1) to include key parameters needed in most death models, and bundle these as Death (Section 11.2.2) within the phenotype, similarly to Cycle (Section 11.1.5).

[Return to Table of Contents.]

11.2.1 Death_Parameters

Death_Parameters bundles key parameters needed to initialize a cell death model, particularly changes in a cell's volume model. The key data elements include:

1. **std::string time_units** gives the time units (by default minutes throughout PhysiCell).
2. **double unlysed_fluid_change_rate** is the rate of fluid change (in min^{-1}) prior to cell lysis.
3. **double lysed_fluid_change_rate** is the rate of fluid change (in min^{-1}) after cell lysis.
4. **double cytoplasmic_biomass_change_rate** is the degradation rate for cytoplasmic solids (in min^{-1}).
5. **double nuclear_biomass_change_rate** is the degradation rate for nuclear solids (in min^{-1}).
6. **double calcification_rate** is the rate of cell calcification (in min^{-1}).
7. **double relative_rupture_volume** is the relative amount by which the cell must swell (compared to the volume at the onset of cell death) before it bursts or lyses.

Here is the formal class declaration:

```
class Death_Parameters
{
 public:
    std::string time_units;

    double unlysed_fluid_change_rate;
    double lysed_fluid_change_rate;

    double cytoplasmic_biomass_change_rate;
```

```
    double nuclear_biomass_change_rate;

    double calcification_rate;

    double relative_rupture_volume;

    Death_Parameters();
};
```

Member functions

1. **`Death_Parameters()`** is the default constructor. It sets all parameter values to the reference apoptosis values for a generic breast epithelium line (calibrated to MCF-10A measurements), as in [3].

[Return to Table of Contents.]

11.2.2 Death

In PhysiCell, we allow the cells to evaluate multiple death models (e.g., apoptosis and necrosis). `Death` stores the cell's death rates, the corresponding death models, and associated parameters. Here are the data elements:

1. **`std::vector<double> rates`** is a vector of death rates, one per death model.
2. **`std::vector<Cycle_Model*> models`** is a vector of pointers to death models, which are of type `Cycle_Model`.
3. **`std::vector<Death_Parameters> parameters`** is a vector of `Death_Parameters`s, one for each `Cycle_Model`.
4. **`bool dead`** is a Boolean variable that is `true` if the cell is dead.
5. **`int current_death_model_index`** is the index of the current death model, when `dead == true`.

Here is the formal class declaration:

```
class Death
{
 private:
 public:
    std::vector<double> rates;
    std::vector<Cycle_Model*> models;
    std::vector<Death_Parameters> parameters;

    bool dead;
    int current_death_model_index;
```

```
    Death();

    int add_death_model( double rate, Cycle_Model* pModel );
    int add_death_model( double rate, Cycle_Model* pModel,
        Death_Parameters& death_parameters);

    int find_death_model_index( int code );
    int find_death_model_index( std::string name );

    bool check_for_death( double dt );
    void trigger_death( int death_model_index );

    Cycle_Model& current_model( void );
    Death_Parameters& current_parameters( void );
};
```

Member functions

1. **`Death()`** is the default construtor. It resizes all the vectors to size zero, sets `dead = false`, and `current_death_model_index = 0`.

2. **`int add_death_model( double rate , Cycle_Model* pModel )`** adds the cycle model at `pModel` and sets its corresponding death rate to `rate`. It also resizes the `parameters` with values in the default constructor. It returns the index of the newly added death model.

3. **`int add_death_model( double rate, Cycle_Model* pModel, Death_Parameters& death_parameters)`** adds the cycle model at `pModel` and sets its corresponding death rate to `rate`. It also increases the size of `parameters` by one by appending `death_parameters`. It returns the index of the newly added death model.

4. **`int find_death_model_index( int code )`** returns an integer `i` such that `models[i]->code == code`. Note that if no exact match is found, this returns 0.

5. **`int find_death_model_index( std::string name )`** returns an integer `i` such that `models[i]->name == name`. Note that if no exact match is found, this returns 0. Note that this function is case sensitive.

6. **`bool check_for_death( double dt )`** checks for each type of cell death in the next `dt` time. If the cell dies by any of the models between now and `dt` time in the future, this code sets `dead = true`, sets `current_death_model_index` to the index of the corresponding death model, and returns `true`.

7. **`void trigger_death( int death_model_index )`** immediately sets the cell to `dead = true` and `current_death_model_index = death_model_index`.

8. **`Cycle_Model& current_model( void )`** returns (by reference) the current cell death model (if `dead == true`).

9. **`Death_Parameters& current_parameters( void )`** returns (by reference) the `Death_Parameters` for the current death model (if `dead == true`).

[Return to Table of Contents.]

11.3 Volume

`Volume` stores the cell's total volume, its various sub-volume elements, and critical parameters. (PhysiCell supplies reasonable defaults.) Here are the main elements (all volume units in PhysiCell are currently assumed to be μm^3.)

1. **`double total`** is the cell's total volume.

2. **`double solid`** is the solid component of the cell's total volume. It is in dimensional units of volume–not a fraction or ratio. Note that `volume.total = volume.solid + volume.fluid`.

3. **`double fluid`** is the fluid component of the cell's total volume. It is in dimensional units of volume–not a fraction or ratio. Note that `volume.total = volume.solid + volume.fluid`.

4. **`double fluid_fraction`** is the fraction of the cell that is fluid, defined as:

   `volume.fluid_fraction = volume.fluid / volume.total`.

   Note that $0 \leq$ `fluid_fraction` ≤ 1.

5. **`double nuclear`** is the total nuclear volume. Note that:

   `volume.total = volume.nuclear + volume.cytoplasmic`.

6. **`double nuclear_solid`** is the solid component of the cell's nuclear volume. It is in dimensional units of volume–not a fraction or ratio. Note that:

   `volume.nuclear = volume.nuclear_solid + volume.nuclear_fluid`.

7. **`double nuclear_fluid`** is the fluid component of the cell's nuclear volume. It is in dimensional units of volume–not a fraction or ratio. Note that:

   `volume.nuclear = volume.nuclear_solid + volume.nuclear_fluid`.

8. **`double cytoplasmic`** is the total cytoplasmic volume. Note that:

   `volume.total = volume.nuclear + volume.cytoplasmic`.

9. **`double cytoplasmic_solid`** is the solid component of the cell's cytoplasmic volume. It is in dimensional units of volume–not a fraction or ratio. Note that:

   `volume.cytoplasmic = volume.cytoplasmic_solid + volume.cytoplasmic_fluid`.

10. **`double cytoplasmic_fluid`** is the fluid component of the cell's cytoplasmic volume. It is in dimensional units of volume–not a fraction or ratio. Note that:

    `volume.cytoplasmic = volume.cytoplasmic_solid + volume.cytoplasmic_fluid`.

11. **`double calcified_fraction`** is the fraction of the cell that is calcified. (This is particularly useful to simulations of ductal carcinoma in situ of the breast.) Note that

$$0 \leq \texttt{calcified\_fraction} \leq 1. \tag{8}$$

12. **`double cytoplasmic_to_nuclear_ratio`** is `volume.cytoplasmic / volume.nuclear`. Note that

$$0 \leq \texttt{cytoplasmic\_to\_nuclear\_ratio} \leq 1. \tag{9}$$

13. **double rupture_volume** is the (dimensional) volume at which a cell will burst or lyse.

14. **double cytoplasmic_biomass_change_rate** is the rate that cytoplasmic solid material can be synthesized to reach the target cytoplasmic solid volume. It is assumed to be written in units of min^{-1}.

15. **double nuclear_biomass_change_rate** is the rate that nuclear solid material can be synthesized to reach the target nuclear solid volume. It is assumed to be written in units of min^{-1}.

16. **double fluid_change_rate** is the rate that fluid can enter or leave the cell to reach the target fluid fraction. It is assumed to be written in units of min^{-1}.

17. **double calcification_rate** is the rate that the cell calcifies. It is assumed to be written in units of min^{-1}.

18. **double target_solid_cytoplasmic** is the cell's target ("desired" or "goal") solid cytoplasmic volume.

19. **double target_solid_nuclear** is the cell's target ("desired" or "goal") solid nuclear volume.

20. **double target_fluid_fraction** is the cell's target ("desired" or "goal") fluid fraction.

21. **double relative_rupture_volume** is the relative volume at which a cell, written as a multiple of the cell's total volume at the onset of a swelling process. (For example, at the start of necrosis.)

    Note that
    $$0 \le \texttt{relative\_rupture\_volume} \le 1. \tag{10}$$

Here is the class declaration:

```
class Volume
{
 public:
    double total;
    double solid;
    double fluid;
    double fluid_fraction;

    double nuclear;
    double nuclear_fluid;
    double nuclear_solid;

    double cytoplasmic;
    double cytoplasmic_fluid;
    double cytoplasmic_solid;

    double calcified_fraction;

    double cytoplasmic_to_nuclear_ratio;

    double rupture_volume;
```

```
    double cytoplasmic_biomass_change_rate;
    double nuclear_biomass_change_rate;
    double fluid_change_rate;

    double calcification_rate;

    double target_solid_cytoplasmic;
    double target_solid_nuclear;
    double target_fluid_fraction;

    double target_cytoplasmic_to_nuclear_ratio;

    double relative_rupture_volume;

    Volume();

    void divide( void );
    void multiply_by_ratio(double);

    void update( Cell* pCell, Phenotype& phenotype, double dt );
};
```

[Return to Table of Contents.]

11.3.1 Member functions

1. **`Volume()`** is the default constructor. It sets the variables to the reference values of a generic breast epithelium line (calibrated to MCF-10A).

2. **`void divide(void)`** cuts the volume, all sub-volumes, and the target volumes by one half. (It is used during cell division.)

3. **`void multiply_by_ratio(double ratio)`** multiplies the volume, all sub-volumes, and the target volumes by `ratio`.

   `void update( Cell* pCell, Phenotype& phenotype, double dt )` is neither used nor implemented. It will be deprecated.

[Return to Table of Contents.]

11.4 Geometry

`Geometry` stores critical aspects of cell geometry other than volume. In the current implementation, we use spherical approximations for the cell geometry, since PhysiCell tracks cell volume but not cell morphology. The main data elements are:

1. **`double radius`** is the cell's equivalent radius, based upon the spherical approximation

$$\texttt{volume.total} = \frac{4}{3}\pi\ \texttt{radius}^3. \tag{11}$$

   In the current version of PhysiCell, spatial units are assumed to be μm.

2. **`double nuclear_radius`** is the nucleus' equivalent radius, based upon the spherical approximation

$$\texttt{volume.nuclear} = \frac{4}{3}\pi\ \texttt{nuclear\_radius}^3. \tag{12}$$

   Its units are assumed μm.

3. **`double surface_area`** is the cell's equivalent surface area, based upon the spherical approximation

$$\texttt{surface\_area} = 4\pi\ \texttt{radius}^2. \tag{13}$$

4. **`double polarity`** is a dimensionless number between 0 and 1 to indicate how polarized the cell is along its basal-to-apical axis. If the polarity is zero, the cell has no discernible polarity. Note that polarity should be set to one for 2-D simulations.

   Its units are assumed μm^2.

Here is the class definition in `PhysiCell_phenotype.h`:

```
class Geometry
{
 public:
    double radius;
    double nuclear_radius;
    double surface_area;

    double polarity;

    Geometry();

    void update_radius( Cell* pCell, Phenotype& phenotype, double dt );
    void update_nuclear_radius( Cell* pCell, Phenotype& phenotype, double dt );
    void update_surface_area( Cell* pCell, Phenotype& phenotype, double dt );

    void update( Cell* pCell, Phenotype& phenotype, double dt );
};
```

[Return to Table of Contents.]

11.4.1 Member functions

1. **Geometry()** is the default constructor. It sets the variables to the reference values of a generic breast epithelium line (calibrated to MCF-10A).

2. **void update_radius(Cell* pCell, Phenotype& phenotype, double dt)** sets `radius` according to the spherical approximation in Section 11.4, using `phenotype.volume.total`.

3. **void update_nuclear_radius(Cell* pCell, Phenotype& phenotype, double dt)** sets `nuclear_radius` according to the spherical approximation in Section 11.4, using `phenotype.volume.nuclear`.

4. **void update_surface_area(Cell* pCell, Phenotype& phenotype, double dt)** sets `surface_area` according to the spherical approximation in Section 11.4, using `cell.phenotype.volume.total`.

5. **void update(Cell* pCell, Phenotype& phenotype, double dt)** sets `radius`, `nuclear_radius`, and `surface_area` according to `phenotype.volume`.

[Return to Table of Contents.]

11.5 Mechanics

`Mechanics` stores the main mechanics parameters for a cell. The main data elements are:

1. **double cell_cell_adhesion_strength** is the parameter C_{cca} in the default PhysiCell mechanics model, written as a multiple of the drag coefficient ν; see [3]. It regulates the relative strength of cell-cell adhesive forces. Future releases of PhysiCell will allow this to be defined for interactions with multiple cell types.

2. **double cell_BM_adhesion_strength** is the parameter C_{cba} in the default PhysiCell mechanics model, written as a multiple of the drag coefficient ν; see [3]. It regulates the relative strength of adhesion of cells to the basement membrane, when present.

3. **double cell_cell_repulsion_strength** is the parameter C_{ccr} in the default PhysiCell mechanics model, written as a multiple of the drag coefficient ν; see [3]. It regulates the relative strength of cell-cell "repulsive" forces (resistance to deformation and compression).

4. **double cell_BM_repulsion_strength** is the parameter C_{cbr} in the default PhysiCell mechanics model, written as a multiple of the drag coefficient ν; see [3]. It regulates the relative strength of cell-BM "repulsive" forces (resistance of cells to deformation and compression, and resistance of basement membranes to penetration and deformation by cells).

5. **double relative_maximum_adhesion_distance** is the maximum distance of cell adhesion to other cells or a basement membrane, given as a (dimensionless) multiple of `geometry.radius`.

6. **double maximum_adhesion_distance** is the absolute maximum distance at which a cell interacts adhesively with other cells or basement membrane. We tend not to use this variable in PhysiCell functions, so it may be deprecated in future releases.

As future releases of PhysiCell may include additional mechanics models, this class may be expanded in the future. In particular, we anticipate models to allow varying strengths of adhesion between different cell types, and improved adhesion models.

Here is the class definition in PhysiCell_phenotype.h:

```
class Mechanics
{
 public:
    double cell_cell_adhesion_strength;
    double cell_BM_adhesion_strength;
    double cell_cell_repulsion_strength;
    double cell_BM_repulsion_strength;

    double relative_maximum_adhesion_distance;
    double maximum_adhesion_distance;

    Mechanics();
};
```

[Return to Table of Contents.]

11.5.1 Member functions

1. **Mechanics()** is the default constructor function. It sets the parameters to the reference value for a generic breast epithelium line.

[Return to Table of Contents.]

11.6 Motility

Motility stores motility parameters and the current speed/direction of motility. It was designed to be sufficiently generic to allow recovery of purely Brownian motion, deterministic taxis, and combinations of these. Here are the main data elements:

1. **bool is_motile** is a Boolean variable that can be used to enable/disable cell motility.

2. **double persistence_time** is the mean time cell continues at its current speed and direction before re-evaluating and choosing a new motility vector. It is assumed to have units minutes.

3. **double migration_speed** is the speed of motility, in the absence of other forces (e.g., cell-cell adhesion). It is assumed to have units of μm/min.

4. **std::vector<double> migration_bias_direction** is the 3-D vector giving the cell's preferred direction of motility for biased Brownian motion. If the user modifies this vector, they must ensure it is a *unit vector*:

$$||\texttt{migration\_bias\_direction}|| = 1. \quad (14)$$

5. **double migration_bias** (with a value in [0,1]) sets the degree to which cell motility is biased along `migration_bias_direction`. If 0, then motion is completely Brownian. If 1, it is completely deterministc along the bias direction.

6. **bool restrict_to_2D** is a Boolean variable that is set to `true` is we are restricting cell motility to 2D.

7. **std::vector<double> motility_vector** is the velocity vector for cell motility, based upon the all the variables and parameters defined above. See also Section 11.6.2.

Here is the class definition:

```
class Motility
{
 public:
    bool is_motile;

    double persistence_time;
    double migration_speed;
    std::vector<double> migration_bias_direction;
    double migration_bias;
    bool restrict_to_2D;
    std::vector<double> motility_vector;

    Motility();
};
```

The main parameters are further defined and related in Section 11.6.2

[Return to Table of Contents.]

11.6.1 Member functions

1. **Motility()** is the default constructor function. Note that it sets:

   `is_motile = false`,
   `update_migration_bias_vector = NULL`, and
   `restrict_to_2D = false`.

[Return to Table of Contents.]

11.6.2 Motility definitions

The direction of (biased random) motility is given by

$$\mathbf{d}_{\mathrm{mot}} = \frac{b\,\mathbf{d}_{\mathrm{bias}} + (1-b)\boldsymbol{\xi}}{||b\,\mathbf{d}_{\mathrm{bias}} + (1-b)\boldsymbol{\xi}||} \tag{15}$$

where b (`migration_bias`) is the level of bias, $\boldsymbol{\xi}$ is a random unit vector (length 1, uniformly random direction), and $\mathbf{d}_{\mathrm{bias}}$ (`migration_bias_direction`) is the directional bias for motility. See Section 9.2 for more information on how the `Motility` class is used when updating a cell's velocity.

[Return to Table of Contents.]

11.7 Secretion

`Secretion` collects the cell's biotransport parameters for interfacing with BioFVM [2]. Its main data elements include:

1. **`Microenvironment* pMicroenvironment`** is a pointer to the correct microenvironment, where substrates have already been declared.
2. **`std::vector<double> secretion_rates`** is a vector of secretion rates for the substrates in the microenvironment.
3. **`std::vector<double> uptake_rates`** is a vector of uptake rates for the substrates in the microenvironment.
4. **`std::vector<double> saturation_densities`** is a vector of densities at which secretions saturate. See [2].

Here is the full class declaration:

```
class Secretion
{
 private:
 public:
    Microenvironment* pMicroenvironment;

    std::vector<double> secretion_rates;
    std::vector<double> uptake_rates;
    std::vector<double> saturation_densities;

    Secretion();

    void sync_to_current_microenvironment( void );
    void advance( Basic_Agent* pCell, Phenotype& phenotype , double dt );

    void sync_to_microenvironment( Microenvironment* pNew_Microenvironment );

    void set_all_secretion_to_zero( void );
    void set_all_uptake_to_zero( void );
    void scale_all_secretion_by_factor( double factor );
    void scale_all_uptake_by_factor( double factor );
};
```

[Return to Table of Contents.]

11.7.1 Member functions

1. **Secretion()** is the default constructor. If a default Microenvironment has already been set in BioFVM, then `pMicroenvironment` is set to this (otherwise `NULL`). Thereafter, `secretion_rates`, `uptake_rates`, and `saturation_densities` are resized for consistency with `pMicroenvironment`, with all values set to 0.0.

2. **void sync_to_current_microenvironment(void)** resizes `secretion_rates`, `uptake_rates`, and `saturation_densities` consistency with `pMicroenvironment` and sets all vector entries to 0.0.

3. **void sync_to_microenvironment(Microenvironment* pNew_Microenvironment)** sets `pMicroenvironment = pNew_Microenvironment`, resizes `secretion_rates`, `uptake_rates`, and `saturation_densities` for consistency with `pMicroenvironment`, and sets all vector entries to 0.0.

4. **void advance(Basic_Agent* pCell, Phenotype& phenotype, double dt)** evaluates the BioFVM secretion and uptake functions for this individual cell. Consistency checks with BioFVM (including those from volume changes) are fully automated.

5. **void set_all_secretion_to_zero(void)** sets all the secretion rates to zero. (Please note that settings `pCell->is_active = false` is the most efficient way to set all secretion and uptake to zero in a cell.)

6. **void set_all_uptake_to_zero(void)** sets all the uptake rates to zero. (Please note that settings `pCell->is_active = false` is the most efficient way to set all secretion and uptake to zero in a cell.)

7. **void scale_all_secretion_by_factor(double factor)** multiplies all the secretion rates by `factor`.

8. **void scale_all_uptake_by_factor(double factor)** multiplies all the uptake rates by `factor`.

[Return to Table of Contents.]

---

12 Cell Containers

PhysiCell uses `Cell_Containers` to help organize and search for cells within the simulation (spatial) domain, and to accelerate cell-cell mechanics. It is overloaded with much of the core functionality of PhysiCell, so we highly recommend that you ***avoid direct operations on cell containers!*** For reference, here is the full class definition:

```
class Cell_Container : public BioFVM::Agent_Container
{
 private:
    std::vector<Cell*> cells_ready_to_divide;
    std::vector<Cell*> cells_ready_to_die;
    int boundary_condition_for_pushed_out_agents;
    bool initialzed = false;

 public:
```

```
    BioFVM::Cartesian_Mesh underlying_mesh;
    std::vector<double> max_cell_interactive_distance_in_voxel;
    int num_divisions_in_current_step;
    int num_deaths_in_current_step;

    double last_diffusion_time =0.0;
    double last_cell_cycle_time = 0.0;
    double last_mechanics_time = 0.0;
    Cell_Container();
    void initialize(double x_start, double x_end,
        double y_start, double y_end, double z_start, double z_end,
        double voxel_size);
    void initialize(double x_start, double x_end,
        double y_start, double y_end, double z_start, double z_end,
        double dx, double dy, double dz);
    std::vector<std::vector<Cell*> > agent_grid;
    std::vector<std::vector<Cell*> > agents_in_outer_voxels;

    void update_all_cells(double t);
    void update_all_cells(double t, double dt);
    void update_all_cells(double t, double phenotype_dt, double mechanics_dt);
    void update_all_cells(double t, double phenotype_dt, double mechanics_dt,
        double diffusion_dt );

    void register_agent( Cell* agent );
    void add_agent_to_outer_voxel(Cell* agent);
    void remove_agent(Cell* agent );
    void remove_agent_from_voxel(Cell* agent, int voxel_index);
    void add_agent_to_voxel(Cell* agent, int voxel_index);

    void flag_cell_for_division( Cell* pCell );
    void flag_cell_for_removal( Cell* pCell );
    bool contain_any_cell(int voxel_index);
};
```

Users may want to use the following function to ensure that the mechanics data structures are set up consistently with BioFVM microenvironment's domain:

```
Cell_Container* create_cell_container_for_microenvironment( BioFVM::Microenvironment& m,
    double mechanics_voxel_size );
```

Here is an example use:

```
// Set mechanics voxel size.
double mechanics_voxel_size = 30;

// Assume microenvironment is defined above somewhere.
```

```
// Set up the PhysiCell mechanics data structure.
Cell_Container* cell_container = create_cell_container_for_microenvironment(
    microenvironment, mechanics_voxel_size );
```

[Return to Table of Contents.]

13 PhysiCell Outputs

PhysiCell supports several methods of output, including SVG files (allowing virtual pathology through a fixed cross-section), MultiCellDS digital snapshots (a single-time save file), and other output methods.

Over the next several releases, we plan further improvements to PhysiCell outputs.

13.1 Virtual Pathology

PhysiCell can simulate transmitted light microscopy to create virtual H&E (hematoxylin and eosin) images, as well as false-colored images. These images are saved as SVG (scalable vector graphics) files, which allow lossless rescaling of the image. Moreover, because SVG files are a specialized XML, users can change labels and other image aspects long after image processing, using simple text editors.

We also note that the SVG functions provided in PhysiCell (`./modules/PhysiCell_SVG.h`) can be compiled independently of PhysiCell.

[Return to Table of Contents.]

13.1.1 SVG functions

SVG functions (defined in `./modules/PhysiCell_SVG.h`) provide basic functionality for creating SVG files. In all the functions below, please note that the origin $(0, 0)$ in an SVG file is in the upper left corner.

1. **`bool Write_SVG_start( std::ostream& os, double width, double height )`** creates the header information for an SVG file of width `width`, height `height`, and writes the stream in `os`.

   Example: Starting a 640 × 480 image:

   ```
   // open the file, write a basic "header"
   std::ofstream os( "sample_SVG.svg" , std::ios::out );
   Write_SVG_start( os, 640, 480 );
   ```

2. **`bool Write_SVG_end( std::ostream& os )`** writes the end of an SVG file to the stream in `os`.

   Example: Starting a 640 × 480 image:

   ```
   // open the file, write a basic "header"
   std::ofstream os( "sample_SVG.svg" , std::ios::out );
   Write_SVG_start( os, 640, 480 );
   ```

```
// operations on the SVG file

// close and save the file
Write_SVG_end(os);
os.close();
```

3. **bool Write_SVG_text(std::ostream& os, const char* str, double position_x, double position_y, double font_size, const char* color , const char* font)** places the text `str` at (upper left) position `[position_x,position_y]` with text height `font_size`, color `color`, and font `font`. It writes to the stream `os`.

   Example: Placing a dark red "hello world" at (30,60), with font height 17 and the Arial font.

```
// open the file, write a basic "header"
std::ofstream os( "sample_SVG.svg" , std::ios::out );
Write_SVG_start( os, 640, 480 );

std::string my_text = "Hello world!";
std::string my_color = "rgb(128,0,0)";
std::string my_font = "Arial";

Write_SVG_text( os, my_text.c_str(), 30, 60, 17, my_color.c_str(),
    my_font.c_str() );

// close and save the file
Write_SVG_end(os);
os.close();
```

4. **bool Write_SVG_circle(std::ostream& os, double center_x, double center_y, double radius, double stroke_size, std::string stroke_color, std::string fill_color)** places a circle with center at `[center_x,center_y]` and radius `radius`. The circle is filled with color `fill_color`, and an outline of thickness `stroke_size` and color `stroke_color`. It writes to the stream `os`.

   Example: Placing a cyan circle with black outline at (100,90), with radius 8.6, and outline width 1.

```
// open the file, write a basic "header"
std::ofstream os( "sample_SVG.svg" , std::ios::out );
Write_SVG_start( os, 640, 480 );

std::string my_fill_color = "rgb(0,255,255)";
std::string my_outline_color = "black";

Write_SVG_circle(os,100,90,8.6,1,my_outline_color.c_str(),my_fill_color.c_str());

// close and save the file
Write_SVG_end(os);
os.close();
```

5. **bool Write_SVG_rect(std::ostream& os,**
   double UL_corner_x, double UL_corner_y,
   double width, double height, double stroke_size,
   std::string stroke_color , std::string fill_color) places a rectangle with upper-left corner `[UL_corner_x,UL_corner_y]`, width `width`, height `height`, filled with color `fill_color`, and outlined with color `stroke_color` and line thickness `stroke_size`. It writes to the stream `os`.

   Example: Placing a black border with no fill around the SVG image.

```
// open the file, write a basic "header"
std::ofstream os( "sample_SVG.svg" , std::ios::out );
Write_SVG_start( os, 640, 480 );

std::string my_fill_color = "none";
std::string my_outline_color = "black";

Write_SVG_rect(OS,0,0,640,480,1,my_outline_color.c_str(),my_fill_color.c_str());

// close and save the file
Write_SVG_end(os);
os.close();
```

6. **bool Write_SVG_line(std::ostream& os,**
   double start_x, double start_y, double end_x, double end_y,
   double thickness, std::string stroke_color) draws a line with color `stroke_color` and thickness `thickness` from `[start_x,start_y]` to `[end_x,end_y]`. It writes to the stream `os`.

   Example: Placing a thin dark blue line from (0,0) to (640,480).

```
// open the file, write a basic "header"
std::ofstream os( "sample_SVG.svg" , std::ios::out );
Write_SVG_start( os, 640, 480 );

std::string my_stroke_color = "rgb(0,0,64)";

Write_SVG_line(os,0,0,640,480,1.5,my_stroke_color.c_str());

// close and save the file
Write_SVG_end(os);
os.close();
```

[Return to Table of Contents.]

13.1.2 Pathology functions

PhysiCell can create fast virtual pathology images through a cross section. Here are the main functions:

1. **void SVG_plot(std::string filename , Microenvironment& M,**
   double z_slice, double time,

std::vector<std::string> (*cell_coloring_function)(Cell*)) creates an SVG plot through $z = $ z_slice, using the coloring function `cell_coloring_function` (See Section 13.1.3), with labeling for time `time`, in the microenvironment `M`, saved to `filename`.

This function checks all cells for intersection with the plane through $z = $ z_slice, and plots the intersecting part of the cell cytoplasm and nucleus (using circular approximations). In the plot, 1 pixel is 1 μm.

Example: H&E plot through $z = 0\mu$m, followed by a false-colored Ki-67 plot through $z = 10\mu$m:

```
SVG_plot( "initial_HE.svg" , microenvironment, 0.0 , t,
    hematoxylin_and_eosin_cell_coloring );
SVG_plot( "initial_Ki67.svg" , microenvironment, 10.0 , t,
    false_cell_coloring_Ki67 );
```

The SVG plotting options are set by `PhysiCell_SVG_options`, which is discussed further in Section 15.6.

2. **std::string formatted_minutes_to_DDHHMM(double minutes)** creates a nicely formated string (in days, hours, and minutes) based upon `minutes`. It is used extensively in `SVG_plot`.

3. **std::vector<double> transmission(std::vector<double>& incoming_light, std::vector<double>& absorb_color, double thickness, double stain)** simulates transmission of light of color `incoming_light` through a tissue of thickness `thickness`, stained at relative intensity `stain` (with range from 0 to 1), which absorbs light of color `absorb_color`. Its output is the transmitted color, as an RGB vector.

   We use a Lambert-Beer [5] light transmission model for each color channel (`Red`, `Green`, `Blue`):

$$\begin{aligned} \texttt{Transmitted}_\texttt{C} &= \texttt{Incoming}_\texttt{C} \cdot \exp\left(- \left(\texttt{thickness} \cdot \texttt{stain} \cdot \frac{1}{255.0} \texttt{Absorb}_\texttt{C} \right) \right), \\ & \texttt{C} \in \{ \texttt{Red}, \texttt{Green}, \texttt{Blue} \} \end{aligned} \tag{16}$$

   Note that we use 24-bit color, so red, green, and blue values should vary from 0 to 255. Outputs to SVG files will be rounded to the nearest integer value. This function is available for use in custom coloring functions, and PhysiCell uses it for virtual H&E stains.

Future releases will include functions to color the background according to data values in the BioFVM microenvironment.

[Return to Table of Contents.]

13.1.3 Cell coloring functions

PhysiCell's virtual pathology functions are built upon choosing a coloring function for the cells. A coloring function takes the form:

```
std::vector<std::string> some_coloring_function( Cell* pCell )
```

and it returns a vector of four strings: the cytoplasm fill color, cytoplasm outline color, nuclear color, and nuclear outline color.

Colors in SVG can be specified as:

1. **RGB colors:** Use a string of the form `"rgb(R,G,B)"`, where `R`, `G`, and `B` are the red, green, and blue values, and they are integers between 0 and 255.

2. **Standard web colors:** Use a string like `"black"` or `"red"`, or use `"none"` for no color (transparent). See https://www.w3.org/TR/SVG11/types.html#ColorKeywords for a list of valid SVG colors.

The following cell coloring functions are provided in PhysiCell:

1. **`simple_cell_coloring`** colors the cytoplasm red (255,0,0), the nucleus blue (0,0,255), and all outlines black.

2. **`false_cell_coloring_Ki67`** is recommended for the Ki67_Basic and Ki67_Advanced cycle models. (See Section 11.1 and Section 16.1.) Pre-mitotic Ki67+ cells are colored green (0,255,0), with a darker green nucleus (0,125,0). Post-mitotic Ki67+ cells are colored magenta (255,0,255), with a darker magenta nucleus (125,0,125). (In the Ki67_Basic model, all Ki67+ cells are green.) Ki67- cells are colored blue (40,200,255) with a darker blue nucleus (20,100,255).

   Apoptotic cells are colored red (255,0,0) with a darker red nucleus (125,0,0). Necrotic cells are colored brown (250,138,38) with a darker brown nucleus (139,69,19). All outlines are black.

3. **`false_cell_coloring_live_dead`** colors live cells green, apoptotic cells red, and necrotic cells brown, with colors defined as the Ki67 coloring function. All outlines are black.

4. **`hematoxylin_and_eosin_cell_coloring`** colors the cytoplasm by first using the `transmission` function, with white (255,255,255) incoming light, an eosin absorb color (2.55,33.15,2.55) [10], a thickness of 20, and a stain intensity given by

   $$\texttt{stain} = \frac{\texttt{pCell->phenotype.volume.cytoplasmic\_solid}}{\texttt{pCell->phenotype.volume.cytoplasmic} + 10^{-10}}, \tag{17}$$

   which approximates the process of staining cytoplasmic solids with eosin, and the water fraction remaining unstained. The result of this simulated transmission is then fed back through the `transmission` function (as the incoming light color), with an hematoxylin absorb color (49.90,51.00,20.40) [10], a thickness of 20, and a stain intensity given by

   $$\texttt{stain} = \texttt{pCell->phenotype.volume.calcified\_fraction} \tag{18}$$

   which approximates the process of staining calcified cytoplasmic solids with hematoxylin.

   The nucleus is colored by virtual hematoxylin staining, with incoming light color white (255,255,255), hematoxylin absorb color (49.90,51.00,20.40) [10], and a stain intensity given by

   $$\texttt{stain} = \frac{\texttt{pCell->phenotype.volume.nuclear\_solid}}{\texttt{pCell->phenotype.volume.nuclear} + 10^{-10}}, \tag{19}$$

   which approximates the process of staining nuclear solids with hematoxylin, and the water fraction remaining unstained.

   All outlines match the corresponding fill colors.

[Return to Table of Contents.]

13.1.4 Examples of custom cell coloring functions

Example: Black nucleus and oxygen-based cytoplasmic coloring

```
std::vector<std::string> oxygen_coloring( Cell* pCell )
{
    std::vector< std::string > output( 4 , "black" );

    // nucleus and both outlines are already black.

    // cytoplasm color

    // first, get the oxygenation

    // determine the o2 index the first time the function is run.
    static int o2_index = microenvironment.find_density_index( "oxygen" );

    // sample the microenvironment at the cell's locaiton
    double o2 = pCell->nearest_density_vector()[o2_index];

    // set the o2 max scale by the cell's parameters.
    static double max_o2 = pCell->parameters.o2_proliferation_saturation;

    // O2 goes from blue (anoxic, 0 mmHg) to red (fully oxygenated).
    int color = (int) round( o2 * 255.0 / max_o2 );
    char szTempString [128];
    sprintf( szTempString , "rgb(%u,0,%u)", color, 255-color );
    output[0].assign( szTempString );

    return output;
}
```

Example: Simulated immunohistochemistry with DAB and a hematoxylin counterstain As in H&E, we assume that hematoxylin stains all nuclear solids, and here we assume that it stains cytoplasmic solids at 10% of the nuclear intensity. We assume that DAB stains a nuclear protein (in custom data). Based on these virtual stains, we use simulated light transmission.

For reference, we use a DAB absorb color of (25.50,53.55,73.95) [10].

```
std::vector<std::string> nuclear_immunostain( Cell* pCell )
{
    std::vector< std::string > output( 4 , "black" );

    // absorb colors
    static std::vector<double> hematoxylin_absorb = {45.90,51.00,20.40};
    static std::vector<double> DAB_absorb = {25.50,53.55,73.95};

    // cytoplasm colors
```

```
    double solid_fraction = 1.0 - pCell->phenotype.fluid_fraction;
    double cyto_stain_intensity = 0.1 * solid_fraction;

    std::vector<double> = color( 3, 255.0 ); // start with white light
    color = transmission(color,hematoxylin_absorb,20,cyto_stain_intensity);

    char szTempString [128];
    sprintf( szTempString , "rgb(%u,%u,%u)",
        (int)round(color[0]), (int)round(color[1]), (int)round(color[2]) );
    output[0].assign( szTempString );
    output[1].assign( szTempString );

    // nuclear colors

    // determine the index of the nuclear protein (assumed between 0 and 1)
    static int protein_index =
        pCell->custom_data.find_variable_index( "nuclear_protein" );
    double nuclear_DAB_stain_intensity = pCell->custom_data[protein_index];
    double nuclear_H_stain_intensity = solid_fraction;

    color = {255.0,255.0,255.0};
    color = transmission(color,hematoxylin_absorb,20,nuclear_H_stain_intensity);
    color = transmission(color,DAB_absorb,20,nuclear_DAB_stain_intensity);

    sprintf( szTempString , "rgb(%u,%u,%u)",
        (int)round(color[0]), (int)round(color[1]), (int)round(color[2]) );
    output[2].assign( szTempString );
    output[3].assign( szTempString );

    return output;
}
```

[Return to Table of Contents.]

13.2 MultiCellDS digital simulation snapshots

PhysiCell saves its data as specialized MultiCellDS digital snapshots [1]. These snapshots save key metadata (See Section 15.5), the microenvironment, and a compact cell readout in an XML file. See [1] and http://multicellds.org/Standards.php for detailed information on the data standard.

To save a MultiCellDS simulation snapshot, use:

```
void save_PhysiCell_to_MultiCellDS_xml_pugi( std::string filename_base,
     Microenvironment& M , double current_simulation_time);
```

Here, the `filebase` determines how all the snapshot sub-files will be named: `filebase.xml`, `filebase_cells.mat`, `filebase_mesh0.mat`, etc.

Example:

```
double t = 0.0;
double dt = 0.01;
double t_max = 30.0 * 24.0 * 60.0; // 30 days
double tolerance = 0.01 * dt;

// other initialization code

// save the initial data
save_PhysiCell_to_MultiCellDS_xml_pugi( "initial" , microenvironment , t );

double next_save_time = t;
double save_interval = 60.0; // save every 60 minutes
output_index = 0;

while( t < t_max + tolerance )
{
    // save if it's time

    if( fabs( t - next_save_time ) < tolerance )
    {
        // make an appropriate file name

        char filename[1024];
        sprintf( filename , "output%08u" , output_index );

        // save

        save_PhysiCell_to_MultiCellDS_xml_pugi( filename , microenvironment , t );

        next_save_time += save_interval;
        output_index++;
    }

    // more simulation steps

    t += dt;
}
```

Here is the overall structure of PhysiCell snapshot

1. XML headers in the `<XML>` tag.
2. MultiCellDS tag, indicating a digital simulation snapshot:
   `<MultiCellDS version="0.5" type="snapshot/simulation">`.
   (a) Metadata in a `<metadata>` tag, including simulation provenance (who ran it, with what software, citation information, etc.), and other notable elements including:
      i. The current simulation time, saved in a tag like
         `<current_time units="min">0.000000</current_time>`.

(b) The BioFVM microenvironment, in a `<microenvironment>` tag structured as:

i. `<domain>` (MultiCellDS can support multiple domains, although we only use one.)

A. `<mesh>` describes the BioFVM mesh. The spatial units are given as an XML attribute of this element. Here is the overall (truncated) structure

```
<mesh type="Cartesian" uniform="true" regular="true" units="micron">
    <bounding_box type="axis-aligned" units="micron" />
    <x_coordinates delimiter=" " />
    <y_coordinates delimiter=" " />
    <z_coordinates delimiter=" " />
    <voxels type="matlab">
        <filename>initial_mesh0.mat</filename> <!-- compact storage -->
    </voxels>
</mesh>
```

The voxel information (in this case, in `initial_mesh0.mat`) is stored with one voxel per column. The first three rows give the x-, y-, and z-coordinates of each voxel's center, respectively. The fourth row gives each voxel's volume.

B. `<variables>` gives a list of variables, including the name (XML attributes), units (XML attributes), and some physical parameters. Here is a (truncated) example:

```
<variables>
    <variable name="oxygen" units="mmHg" ID="0">
        <physical_parameter_set>
            <conditions />
            <diffusion_coefficient units="micron^2/min" />
            <decay_rate units="1/min" />
        </physical_parameter_set>
    </variable>
</variables>
```

C. Microenvironment data, typically stored compactly in a MATLAB file, like this:

```
<data type="matlab">
    <filename>initial_microenvironment0.mat</filename>
</data>
```

The data file (here, `initialize_microenvironment0.mat`) stores the microenvironment in one column per voxel, and if there are n substrates, n+3 rows, where rows one to three are the (x,y,z) coordinates of the voxel, and each subsequent row is a variable value (as defined in `<variables>` above).

ii. Cellular information, here in a customized and compact MultiCellDS format:

```
<cellular_information>
   <cell_populations>
      <cell_population type="individual">
         <custom>
            <simplified_data type="matlab" source="BioFVM">
               <filename>initial_cells.mat</filename>
            </simplified_data>
            <simplified_data type="matlab" source="PhysiCell">
               <labels>
                  <label index="0" size="1">ID</label>
                  <label index="1" size="3">position</label>
```

```
                              <label index="4" size="1">total_volume</label>
                              <label index="5" size="1">cell_type</label>
                              <label index="6" size="1">cycle_model</label>
                              <label index="7" size="1">current_phase</label>
                              <label index="8" size="1">elapsed_time_in_phase</label>
                              <label index="9" size="1">nuclear_volume</label>
                              <label index="10" size="1">cytoplasmic_volume</label>
                              <label index="11" size="1">fluid_fraction</label>
                              <label index="12" size="1">calcified_fraction</label>
                              <label index="13" size="3">orientation</label>
                              <label index="16" size="1">polarity</label>
                          </labels>
                          <filename>initial_cells_physicell.mat</filename>
                      </simplified_data>
                  </custom>
              </cell_population>
          </cell_populations>
      </cellular_information>
```

In the indicated MATLAB file, each cell is stored in a separate column, with cell-scale data given in separate rows as defined in `<labels>` above. We anticipate improvements in this MultiCellDS output in future editions, but the data will always be structured as above with labels. We will also always preserve the ordering of the first 17 data elements for compatibility.

In the case above, the first row is the cell ID, the next three rows are the cell (x,y,z) position, the next row is the cell's (integer) type, the next row gives the cell's cycle model (as a PhysiCell::constants integer), and so forth.

[Return to Table of Contents.]

13.2.1 Reading PhysiCell snapshots in MATLAB

In the `./matlab` directory, we include a few MATLAB functions to parse the PhysiCell MultiCellDS XML files and associated .mat files. We recommend copying them to your path, or to the same directory as your data.

Here is an example of loading a snapshot (in MATLAB), plotting a microenvironment variable, and then plotting some cells.

```
MCDS = read_MultiCellDS_xml( 'initial.xml' );
% output looks like this
% Elapsed time is 0.522643 seconds.
%
% Summary for file initial.xml:
% Voxels: 40000
% Substrates: 1
%   oxygen (mmHg)
% Cells: 21845
```

```
%
contour( MCDS.mesh.X , MCDS.mesh.Y, MCDS.continuum_variables(1).data(:,:,1) )
axis ij
axis square
% label using MCDS metadata
xlabel( sprintf('x (%s)', MCDS.metadata.spatial_units) , 'fontsize', 13 )
ylabel( sprintf('y (%s)', MCDS.metadata.spatial_units) , 'fontsize', 13 )
% more labeling
title( sprintf('%s at time %3.2f %s', MCDS.continuum_variables(1).name , ...
MCDS.metadata.current_time , MCDS.metadata.time_units) , 'fontsize' , 14 );
% add a colorbar, and label
c = colorbar;
c.Label.String = sprintf( '%s (%s)', MCDS.continuum_variables(1).name , ...
MCDS.continuum_variables(1).units );
c.FontSize = 13;
%
%
% Now, plot the cell positions in 3D
figure
plot3( MCDS.discrete_cells.state.position(:,1) , ...
MCDS.discrete_cells.state.position(:,2), ...
MCDS.discrete_cells.state.position(:,3) , 'ko' );
% labeling
axis equal
xlabel( sprintf('x (%s)', MCDS.metadata.spatial_units) , 'fontsize', 13 )
ylabel( sprintf('y (%s)', MCDS.metadata.spatial_units) , 'fontsize', 13 )
zlabel( sprintf('z (%s)', MCDS.metadata.spatial_units) , 'fontsize', 13 )
```

We anticipate further refinements to MATLAB plotting, and improvements to importing this data beyond MATLAB. Please also note that the current matlab read script uses the reduced matlab file stored in <simplified_data type="matlab" source="BioFVM">. Future PhysiCell releases will use the more complete <simplified_data type="matlab" source="PhysiCell">.

Unfortunately, Octave does not yet ship with xmlread, so the example above will not fully run. You must get the xerces java binaries and xmlread (in the IO octave forge package). There are instructions there on how to get your xerxes JAR files in the path. Please note that xmlread is about one order of magnitude slower on Octave than in Matlab.

[Return to Table of Contents.]

13.3 Other outputs

PhysiCell also has limited POV-ray support (visualization by the open source POV-Ray raytracer), and some additional logging. This will be further documented soon.

14 Key initialization functions

The following initialization functions should be called (in this order):

1. **`omp_set_num_threads(omp_num_threads);`** to set the number of threads in the simulation.

2. **`SeedRandom();`** to initialize the random number generator. (It generates a random seed based on the current system time.) You can also use **`long SeedRandom( long input );`** to specify the random seed, such as to continue a simulation.

3. **`initialize_microenvironment();`** to initialize the microenvironment, after setting any options in `default_microenvironment_options`. See Section 8.2.

4. **`Cell_Container* cell_container = create_cell_container_for_microenvironment( microenvironment, mechanics_voxel_size );`**. Note that `microenvironment` is the default PhysiCell microenvironment. See Section 12.

5. **`initialize_default_cell_definition();`** sets up the default `Cell_Definition` and makes sure it is self-consistent. It also automatically runs setup functions to create standard cell cycle and death models. Users creating new cell definitions are encouraged to copy the default cell definition and then modify it.

All these initialization functions are included in the 2-D and 3-D project templates. See Section 6.

[Return to Table of Contents.]

---

15 Key global data structures

PhysiCell (and BioFVM) have numerous global data structures to help configure options. Here, we detail the most useful of these.

15.1 Global strings

The BioFVM and PhysiCell versions and strings can be accessed via

```
std::string BioFVM_version;
std::string BioFVM_URL;
std::string PhysiCell_version;
std::string PhysiCell_URL;
```

15.2 Default microenvironment

The name of the default microenvironment in PhysiCell is `microenvironment`, which is declared in `./core/PhysiCell_phenotype.cpp`. This strcture is initialized with the function

```
initialize_microenvironment()
```

based upon the settings in `default_microenvironment_options` (of type `Microenvironment_Options`). Here is the definition of that data structure:

```
class Microenvironment_Options
{
 private:

 public:
    Microenvironment* pMicroenvironment;
    std::string name;

    std::string time_units;
    std::string spatial_units;
    double dx;
    double dy;
    double dz;

    bool outer_Dirichlet_conditions;
    std::vector<double> Dirichlet_condition_vector;

    bool simulate_2D;
    std::vector<double> X_range;
    std::vector<double> Y_range;
    std::vector<double> Z_range;

    Microenvironment_Options();

    bool calculate_gradients;
};
```

See Section 8.2 for more details on these options.

[Return to Table of Contents.]

15.3 Default cell definition

In Section 9.4.5, we documented the data structure for a Cell Definition (similar to a MultiCellDS digital cell line [1]).

PhysiCell creates a default `Cell_Definition` called `cell_defaults`. Once the user calls `initialize_default_cell_definition()` (Section 14), this default definition is set to parameter values for the a generic breast epithelium line (calibrated to MCF-10A).

1. It uses the Ki-67 Advanced model. (See Section 16.1.3.)
2. It defaults to apoptosis and necrosis death models, with parameters for a generic breast epithelium line (calibrated to MCF-10A) as given in [3].

3. It uses general breast epithelial parameters (calibrated to MCF-10A) for volume, and the standard volume regulation model
`standard_volume_update_function`. (See Section 16.3.)

4. It sets the cell to be non-motile.

5. It sets the custom rule (`custom_cell_rule`) to `NULL` and update phenotype function (`update_phenotype`) to `update_cell_and_death_parameters_O2_based`, so the cell changes its cycle entry rate and necrosis rate according to its local oxygenation conditions. (See Section 16.6.)

6. It uses the default mechanics model in [3], by setting `update_velocity` equal to `standard_update_cell_velocity`. (See Section 16.4.)

When `create_cell()` or the default `Cell` constructor is called, this default definition is used. (See Section 9.3.)

Users can modify `cell_defaults` at any time; the changes will only apply to cells created after that point in time.

[Return to Table of Contents.]

15.4 A list of all cells

PhysiCell keeps a list of all current cells in the simulation:

```
std::vector<Cell*> *all_cells;
```

It is critical that users ***do not modify this data structure***, but it is available for peforming an operation on all cells.

Example: Dislay the cycle phase of all cells

```
Cell* pC = NULL;
for( int i=0; i < (*all_cells).size(); i++ )
{
    pC = (*all_cells)[i];
    std::cout << pC->ID << ": " << pC->phenotype.cycle.current_phase().name << std::endl;
}
```

[Return to Table of Contents.]

15.5 MultiCellDS options

MultiCellDS embeds program and user metadata each time it saves. You can modify this information. BioFVM (and hence PhysiCell) saves the default metadata in `BioFVM_metadata` (of type `MultiCellDS_Metadata`). Here is the full class definition:

```
class MultiCellDS_Metadata
{
 private:
 public:
    std::string MultiCellDS_type;

    Software_Metadata program;
    Citation_Metadata data_citation;

    // scientific information
    std::string spatial_units;
    std::string time_units;
    std::string runtime_units;
    double current_time;
    double current_runtime;

    std::string description; // any optional text -- not implemented

    MultiCellDS_Metadata();
    void display_information( std::ostream& os);
    void sync_to_microenvironment( Microenvironment& M );
    void restart_runtime( void );

    void add_to_open_xml_pugi( double current_simulation_time,
        pugi::xml_document& xml_dom );
};
```

The various member functions are used by BioFVM/PhysiCell when saving a simulation snapshot, and are not needed for users. We define `Software_Metadata`, `Citation_Metadata`, and `Person_Metadata` (currently unused) below. The other metadata elements (`spatial_units`, etc.) are as expected.

[Return to Table of Contents.]

15.5.1 Software metadata

```
class Software_Metadata
{
 private:
 public:
    // basic program information
    std::string program_name;
    std::string program_version;
    std::string program_URL;

    Person_Metadata creator;
    Person_Metadata user;
    Citation_Metadata citation;
```

```
    Software_Metadata();

    void display_information( std::ostream& os );
    void insert_in_open_xml_pugi( pugi::xml_node& insert_here );
};
```

[Return to Table of Contents.]

15.5.2 Citation metadata

```
class Citation_Metadata
{
 private:
 public:
    std::string DOI;
    std::string PMID;
    std::string PMCID;
    std::string text;
    std::string notes;
    std::string URL;

    Citation_Metadata();
    void display_information( std::ostream& os );
    void insert_in_open_xml_pugi( pugi::xml_node& insert_here );
};
```

[Return to Table of Contents.]

15.5.3 Person metadata

```
class Person_Metadata
{
 private:
    bool is_empty;
 public:
    std::string type; // author, creator, user, curator
    std::string surname;
    std::string given_names;
    std::string email;
    std::string URL;
    std::string organization;
    std::string department;
    std::string ORCID;

    Person_Metadata( );
    void display_information( std::ostream& os );
    void insert_in_open_xml_pugi( pugi::xml_node& insert_here );
};
```

[Return to Table of Contents.]

15.6 SVG options

PhysiCell keeps a simple data structure named `PhysiCell_SVG_options` (of type `PhysiCell_SVG_options_struct`) to easily set SVG plotting options (particularly in digital pathology; see Section 13.1). Here is how that data structure is defined (with its default values):

```
struct PhysiCell_SVG_options_struct
{
    bool plot_nuclei = true;

    std::string simulation_time_units = "min";
    std::string mu = "&#956;";
    std::string simulation_space_units = "&#956;m";

    std::string label_time_units = "days";

    double font_size = 200;
    std::string font_color = "black";
    std::string font = "Arial";

    double length_bar = 100;
};
```

All plots are performed in the simulation units, so a font of height 200 is 200 μm tall.

[Return to Table of Contents.]

15.7 PhysiCell Constants

The structure `PhysiCell_constants` (in `./core/PhysiCell_constants.h`) defines many standardized constants and integer identifiers. They can be accessed with syntax like this:

```
std::cout << "Here is the code for the Ki67 Advanced model: "
          << PhysiCell_constants::advanced_Ki67_cycle_model << std::endl;

Cell* pC = NULL;
for( int i=0; i < (*all_cells).size(); i++ )
{
   pC = (*all_cells)[i];
   if( pC->phenotype.cycle.current_phase().code ==
       PhysiCell_constants::Ki67_negative )
   {
      std::cout << "Cell is Ki67 negative" << std::endl;
```

```
   }
}
```

Here is the list of all current constants (in Version 1.2.0):

```
class PhysiCell_constants
{
 public:
    static constexpr double pi=3.1415926535897932384626433832795;

    static constexpr double cell_removal_threshold_volume = 20;
        // 20 cubic microns -- about 1% of typical cell
    static const int keep_pushed_out_cells_in_outer_voxel=1;
    static const int solid_boundary = 2;
    static const int default_boundary_condition_for_pushed_out_agents
        = keep_pushed_out_cells_in_outer_voxel;

    static const int deterministic_necrosis = 0;
    static const int stochastic_necrosis = 1;

    static const int oxygen_index = 0; // deprecate
    static const int glucose_index = 1; // deprecate

    static const int TUMOR_TYPE=0; // deprecate
    static const int VESSEL_TYPE=1; // deprecate

    static const int mesh_min_x_index=0;
    static const int mesh_min_y_index=1;
    static const int mesh_min_z_index=2;
    static const int mesh_max_x_index=3;
    static const int mesh_max_y_index=4;
    static const int mesh_max_z_index=5;

    static const int mesh_lx_face_index=0;
    static const int mesh_ly_face_index=1;
    static const int mesh_lz_face_index=2;
    static const int mesh_ux_face_index=3;
    static const int mesh_uy_face_index=4;
    static const int mesh_uz_face_index=5;

    // currently recognized cell cycle models
    static const int advanced_Ki67_cycle_model= 0;
    static const int basic_Ki67_cycle_model=1;
    static const int flow_cytometry_cycle_model=2;
    static const int live_apoptotic_cycle_model=3;
    static const int total_cells_cycle_model=4;
    static const int live_cells_cycle_model = 5;
```

```
    // currently recognized death models
    static const int apoptosis_death_model = 6;
    static const int necrosis_death_model = 7;
    static const int autophagy_death_model = 8;

    static const int custom_cycle_model=9999;

    // currently recognized cell cycle and death phases
    // cycle phases
    static const int Ki67_positive_premitotic=0;
    static const int Ki67_positive_postmitotic=1;
    static const int Ki67_positive=2;
    static const int Ki67_negative=3;
    static const int G0G1_phase=4;
    static const int G0_phase=5;
    static const int G1_phase=6;
    static const int G1a_phase=7;
    static const int G1b_phase=8;
    static const int G1c_phase=9;
    static const int S_phase=10;
    static const int G2M_phase=11;
    static const int G2_phase=12;
    static const int M_phase=13;
    static const int live=14;

    static const int custom_phase = 9999;
    // death phases
    static const int apoptotic=100;
    static const int necrotic_swelling=101;
    static const int necrotic_lysed=102;
    static const int necrotic=103;
    static const int debris=104;
};

static std::string time_units = "min";
static std::string space_units = "micron";
static double diffusion_dt = 0.01;
static double mechanics_dt = 0.1;
static double phenotype_dt = 6.0;
```

[Return to Table of Contents.]

16 Standard models

PhysiCell includes several models for easier out-of-the-box simulation. We document here the underlying mathematics of these models, as also presented in [3].

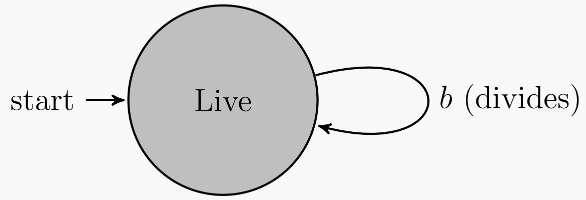

Figure 1: The graph structure of the Live cell cycle model. The default phase is marked "start."

16.1 Cell Cycle Models

PhysiCell includes several pre-built cell cycle models. More models will be added in future releases.

16.1.1 Live (`live`) code: `PhysiCell_constants::live_cells_cycle_model`)

This `Cycle_Mode` has the following `Phases`:

1. **Phase 0:** Named `live` with code `PhysiCell_constants::live`.

In this model, live cells can divide into two live cells, with birth rate b. See Fig. 1. The population-scale model is given by:

$$\frac{dL}{dt} = bL. \tag{20}$$

In PhysiCell, the transition rate from the Live state to the Live state is

$$\texttt{transition\_rate}(0,0) = b. \tag{21}$$

Further details on the *biology* of this model (including details the placement of daughter cells and changes in cell volume) and reference parameter values can be found in [3].

[Return to Table of Contents.]

16.1.2 Ki-67 Basic (`Ki67_basic`) (Code: `PhysiCell_constants::basic_Ki67_cycle_model`)

This `Cycle_Mode` has the following `Phases`:

1. **Phase 0:** Named `Ki67-` with code `PhysiCell_constants::Ki67_negative`.
2. **Phase 1:** Named `Ki67+` with code `PhysiCell_constants::Ki67_positive`.

In this model, Ki67- cells (those staining negative for the cell proliferation marker Ki67) can enter the cell cycle to become Ki67+ cells, at transition rate r_{01}. Ki67+ cells divide into two Ki67- cells at rate r_{10}. See

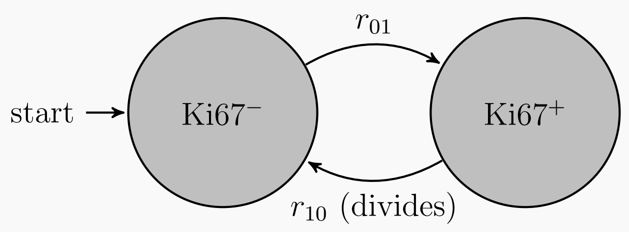

Figure 2: The graph structure of the Ki67 Basic cell cycle model. The default phase is marked "start."

Fig. 2. The population-scale model is given by:

$$\frac{d\,[\mathrm{Ki67}-]}{dt} = -r_{01}\,[\mathrm{Ki67}-] + 2r_{10}\,[\mathrm{Ki67}+] \tag{22}$$

$$\frac{d\,[\mathrm{Ki67}+]}{dt} = r_{01}\,[\mathrm{Ki67}-] - r_{10}\,[\mathrm{Ki67}+] \tag{23}$$

Further details on the *biology* of this model (including details the placement of daughter cells and changes in cell volume) and reference parameter values can be found in [3].

[Return to Table of Contents.]

16.1.3 Ki-67 Advanced (`Ki67_advanced`) (code: `PhysiCell_constants::advanced_Ki67_cycle_model`)

This `Cycle_Mode` has the following `Phases`:

1. **Phase 0:** Named `Ki67-` with code `PhysiCell_constants::Ki67_negative`. This is the default phase of the model.

2. **Phase 1:** Named `Ki67+ (premitotic)` with code `PhysiCell_constants::Ki67_positive_premitotic`.

3. **Phase 2:** Named `Ki67+ (postmitotic)` with code `PhysiCell_constants::Ki67_positive_postmitotic`.

In this model, Ki67- cells (those staining negative for the cell proliferation marker Ki67) can enter the cell cycle to become premitotic Ki67+ cells, at transition rate r_{01}. Ki67+ premitotic cells divide into two Ki67+ postmitotic cells at rate r_{12}. Postmitotic Ki67+ cells become Ki67- cells at rate r_{20}. See Fig. 3. The population-scale model is given by:

$$\frac{d\,[\mathrm{Ki67}-]}{dt} = -r_{01}\,[\mathrm{Ki67}-] + r_{10}\,[\mathrm{Ki67}+(\mathrm{post})] \tag{24}$$

$$\frac{d\,[\mathrm{Ki67}+(\mathrm{pre})]}{dt} = r_{01}\,[\mathrm{Ki67}-] - r_{12}\,[\mathrm{Ki67}+(\mathrm{pre})] \tag{25}$$

$$\frac{d\,[\mathrm{Ki67}+(\mathrm{post})]}{dt} = 2r_{12}\,[\mathrm{Ki67}+(\mathrm{pre})] - r_{20}\,[\mathrm{Ki67}+(\mathrm{post})] \tag{26}$$

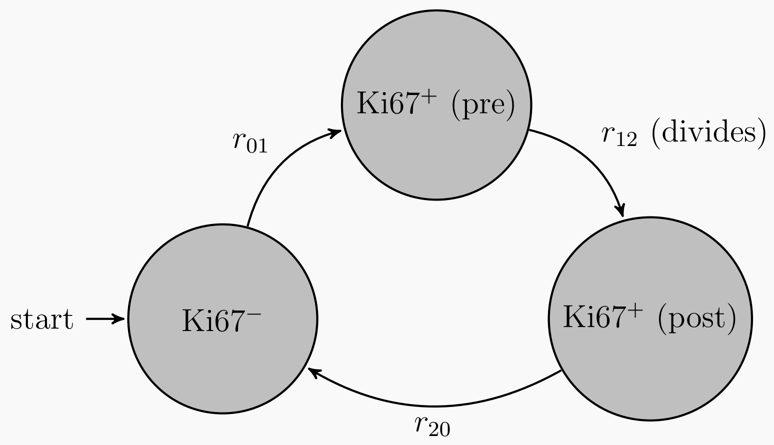

Figure 3: The graph structure of the Ki67 Advanced cell cycle model. The default phase is marked "start."

Further details on the *biology* of this model (including details the placement of daughter cells and changes in cell volume) and reference parameter values can be found in [3].

[Return to Table of Contents.]

16.2 Death Cycle Models

PhysiCell currently includes two pre-built death cycle models. More models may be added in future releases, such as autophagy.

16.2.1 Apoptosis (`apoptosis`) (code: `PhysiCell_constants::apoptosis_death_model`)

This `Cycle_Mode` has the following `Phases`:

1. **Phase 0:** Named `Apoptotic` with code `PhysiCell_constants::apoptotic`.

In this model, apoptotic cells shrink and exit the phase at rate r_{01} (with fixed duration $1/r_{01}$). Cells are removed from the simulation at the end of the apoptotic phase. (PhysiCell currently uses a dummy "debris" phase to avoid coding a phase transition from the apoptotic phase to the apoptotic phase; this may be removed in future releases.) See Fig. 4. The population-scale model is given by:

$$\frac{d\,[\text{Apoptotic}]}{dt} = -r_{01}\,[\text{Apoptotic}]\,. \tag{27}$$

Further details on the *biology* of this model (including details on changes in cell volume) and reference parameter values can be found in [3].

[Return to Table of Contents.]

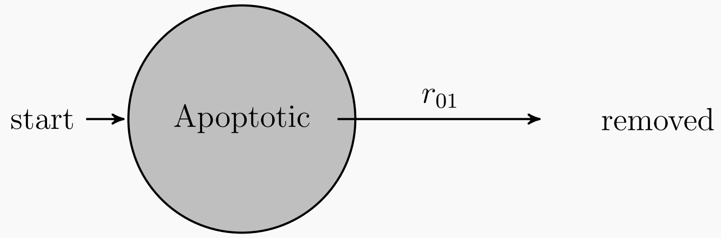

Figure 4: The graph structure of the Apoptosis death cycle model. The default phase is marked "start," and the cell is removed as marked.'

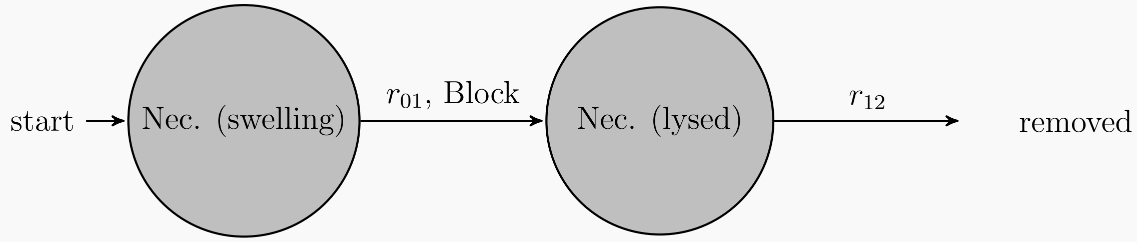

Figure 5: The graph structure of the Necrosis death cycle model. The default phase is marked "start," and the cell is removed as marked. The transition from the unlysed to lysed state is blocked until the cell volume reaches sufficient volume.

16.2.2 Necrosis (`necrosis`) (code: `PhysiCell_constants::necrosis_death_model`)

This `Cycle_Mode` has the following `Phases`:

1. **Phase 0:** Named `Necrotic (swelling)` with code `PhysiCell_constants::necrotic_swelling`.
2. **Phase 1:** Named `Necrotic (lysed)` with code `PhysiCell_constants::necrotic_lysed`.

In this model, unlysed necrotic cells swell and attempt to transition to the lysed necrotic phase. There is a block on the transition until the cell reaches a sufficient total volume. Lysed cells gradually shrink (and calcify, if enable) and exit the phase at rate r_{12}. Cells are removed from the simulation at the end of the lysed necrotic phase. (PhysiCell currently uses a dummy "debris" phase to avoid coding a phase transition from the lysed necrotic phase phase to another necrotic phase; this may be removed in future releases.) See Fig. 5.

Further details on the *biology* of this model (including details on changes in cell volume) and reference parameter values can be found in [3].

[Return to Table of Contents.]

16.3 Volume model (standard_volume_update_function)

The standard volume model separately evolves the total fluid volume, cytoplasmic solid volume, and nuclear solid volume, as a system of ODEs (given in [3]). The standard model also updates cell calcification (but the default rate parameter is zero). See Section 11.3 for more information on the Volume class in the Phenotype, and [3] for the biological details of this model.

[Return to Table of Contents.]

16.4 Cell velocity model (standard_update_cell_velocity)

In this model, the cell uses the mechanics interaction data structure to find nearby cells, adds the contributions from adhesion and "repulsion,", adds the effects of basement membrane interactions (by calling functions.add_cell_basement_membrane_interactions), then computes the contribution to cell velocity by the motility model (by calling update_motility_vector, which in turn calls functions.update_migration_bias). See Section 11.6 for details on motility, Section 11.5 for key cell mechanics phenotype parameters, and Section 9 for more information on Cells and their member functions.

[Return to Table of Contents.]

16.5 Up orientation model (up_orientation)

By default 3-D cells have no preferred orientation, and 2-D cells have an "up" orientation (set to [0,0,1]) to ensure they stay in the $z = 0$ plane. This supplied function is up_orientation.

16.6 Oxygen-dependent phenotype (update_cell_and_death_parameters_O2_based)

Oxygen-dependent cell proliferation and death rates are so commonly needed in cancer models that we include a model function as standard: update_cell_and_death_parameters_O2_based. Here is the overall model for proliferation:

1. Sample the microenvironment for the oxygen concentration σ at the cell center.
2. If $\sigma \geq$ [parameters.o2_proliferation_saturation], then the cycle entry rate is set to 100% of the entry rate in the pReference_live_phenotype reference phenotype.
3. If $\sigma \leq$ [parameters.o2_proliferation_threshold], then the cycle entry rate is set to 0% of the entry rate in the pReference_live_phenotype reference phenotype.
4. Otherwise, the reference cycle entry rate is scaled by

$$\frac{\sigma - [\texttt{parameters.o2\_proliferation\_threshold}]}{[\texttt{parameters.o2\_proliferation\_saturation}] - [\texttt{parameters.o2\_proliferation\_threshold}]} \tag{28}$$

Here is the overall model for the necrosis death rate:

1. Sample the microenvironment for the oxygen concentration σ at the cell center.
2. If $\sigma \geq$ [parameters.o2_necrosis_threshold], then the necrotic death rate is zero.
3. If $\sigma \leq$ [parameters.o2_necrosis_max], then the necrosis death rate is set to 100% of `parameters.max_necrosis_rate`.
4. Otherwise, the necrotic death rate is scaled by

$$\frac{[\texttt{parameters.o2\_necrosis\_threshold}] - \sigma}{[\texttt{parameters.o2\_necrosis\_threshold}] - [\texttt{parameters.o2\_necrosis\_max}]} \tag{29}$$

This function currently works for the following cell cycle models:

1. The Ki67 advanced model (`PhysiCell_constants::advanced_Ki67_cycle_model`). See Section 16.1.3.
2. The Ki67 basic model (`PhysiCell_constants::basic_Ki67_cycle_model`). See Section 16.1.2.
3. The Live model (`PhysiCell_constants::live_cells_cycle_model`). See Section 16.1.1.

See Section 11.1 for more on the cell cycle portion of the phenotype, Section refsec:Death for more information on the death phenotype elements, Section 16.2 on standard death models, and Section 16.1 on standard cell cycle models. See [3] for more on the biology of these models.

[Return to Table of Contents.]

---

17 Examples

17.1 Working with `Cell` functions

17.1.1 Example: a custom volume model

Here, we will define a simpler volume model that only upates the total volume. We use the mathematical model:

$$\frac{d}{dt}[\texttt{volume.total}] = r\left(V^* - [\texttt{volume.total}]\right), \tag{30}$$

where

$$V^* = \left(\frac{1 + [\texttt{target\_cytoplasmic\_to\_nuclear\_ratio}]}{1 - [\texttt{volume.fluid\_fraction}]}\right) \cdot [\texttt{volume.target\_solid\_nuclear}] \tag{31}$$

$$r = \ln\left(\frac{1 - 0.95}{0.5}\right)[\texttt{phenotype.cycle.model.transition\_rate(0,0)}] \tag{32}$$

(This uses the built-in phenotype to get the "target" total volume in terms of the built-in targets, and it sets r so that a divided cell reaches 95% of its target within the mean duration of the cell's interdivision time.)

```
void simple_volume_function( Cell* pCell, Phenotype& phenotype, double dt )
{
    double V_target = phenotype.volume.target_solid_nuclear *
        (1.0 + phenotype.volume.target_cytoplasmic_to_nuclear_ratio) /
        (1-phenotype.volume.fluid_fraction);
    double rate = phenotype.cycle.model().transition_rate(0,0) * log(0.1);

    double addme = V_target;
    addme -= phenotype.volume.total;
    addme *= rate;
    addme *= dt; // dt*rate*( V_target - V )

    phenotype.volume.total += addme;

    return;
}
```

[Return to Table of Contents.]

17.1.2 Example: a custom migration bias

In this example, cells migrate along oxygen gradients. The migration speed is fastest in regions of low oxygen, and the migration is least stochastic (most biased) as the oxygenation increases.

```
void chemotaxis_bias_function( Cell* pCell, Phenotype& phenotype , double dt )
{
    // quickly find O2
    static int O2_index = microenvironment.find_density_index( "oxygen" );

    // sample O2
    double o2 = pCell->nearest_density_vector()[O2_index];

    // set direction along O2 gradients
    phenotype.motility.migration_bias_direction = pCell->nearest_gradient(O2_index);
    normalize( &( phenotype.motility.migration_bias_direction ) );

    // set speed proportional to O2, scaled by normoxic O2 ( 160 mmHg);
    // with a maximum of 1.2 micron per minute
    double theta = o2 / 160.0;
    phenotype.motility.migration_speed = 1.2*(1.0-theta);
    if( phenotype.motility.migration_speed > 1.2 )
    { phenotype.motility.migration_speed = 1.2; }

    // the greater the oxygen, the more biased the motion
    phenotype.motility.migration_bias = theta;

    return;
}
```

17.1.3 Example: a custom cell rule

In this example, an immune cell tests nearby cells first for contact, and then for expression of an antigen (a custom variable). The immune cell initiates apoptosis in the target cell with probability that scales with the antigen expression.

```
void immune_cell_rule( Cell* pCell, Phenotype& phenotype, double dt )
{
    static int antigen_index =
        pCell->custom_data.find_variable_index( "antigen" );
    static int activation_index =
        pCell->custom_data.find_variable_index( "activation" );

    static int apoptosis_model_index =
        pCell->phenotype.death.find_death_model_index( "apoptosis" );

    // exit if immune function is not yet stimulated, or dead
    if( pCell->custom_data[activation_index] < 1e-3
        || pCell->phenotype.death.dead == true )
    { return; }

    std::vector<Cell*> nearby = pCell->cells_in_my_container();

    // test antigen on nearby cells. stop if you find one
    // Don't try to kill dead cells.
    // Don't kill yourself
    Cell* pC = NULL;
    bool stop = false;
    int i=0;

    while( !stop && i < nearby.size() )
    {
        pC = nearby[i];
        if( pC->custom_data[antigen_index] > 1e-3 &&
            pC->phenotype.death.dead == false &&
            pC != pCell )
        { stop = true; }
        i++;
        if( stop == false )
        { pC = NULL; }
    }

    // if we found a cell, attempt to kill it
    if( pC )
    {
```

```
        double probability = 1.0; // dt*pC->custom_data[antigen_index];
        if( UniformRandom() < probability )
        {
            std::cout << "death!" << std::endl;
            system("pause");
            pC->start_death( apoptosis_model_index );
        }
    }
    return;
}
```

Here is the code to add the custom variables to a specific cell:

```
pCell->custom_data.add_variable( "antigen", "dimensionless", 0.0 );
pCell->custom_data.add_variable( "activation", "dimensionless", 0.0 );
```

Better still, you could make cell definitions for tumor cells and immune cells:

```
Cell_Definition immune_cell;

// operations to define this type

immune_cell.functions.custom_cell_rule = immune_cell_rule;
immune_cell.custom_data.add_variable( "activation", "dimensionless", 0.0 );

Cell_Definition MCF7 = cell_defaults;
MCF7.custom_data.add_variable( "antigen", "dimensionless", 0.0 );
```

[Return to Table of Contents.]

17.1.4 Example: a custom phenotype update function

Let's create a custom phenotype update rule that uses the the standard oxygen-based cell birth and death rates, but also increases cell motility and decreases cell-cel adhesion in low oxygen conditions.

Note that these effects gradually turn on and then saturate based on oxygen values in the cell's `parameters`, and we use the cell's `pReference_live_phenotype`.

```
void custom_o2_phenotype_rule( Cell* pCell, Phenotype& phenotype, double dt )
{
   // don't bother if you're dead
   if( pCell->phenotype.death.dead == true )
   { return; }

   // first, call the standard function
   update_cell_and_death_parameters_O2_based(pCell,phenotype,dt);
```

```
   // next, let's evaluate the oxygen
   static int o2_index = microenvironment.find_density_index("oxygen");

   double o2 = pCell->nearest_density_vector()[o2_index];

   if( o2 > pCell->parameters.o2_hypoxic_response )
   { return; }

   // interpolation variable
   double theta = ( pCell->parameters.o2_hypoxic_response  - o2 )/
      (pCell->parameters.o2_hypoxic_response - pCell->parameters.o2_hypoxic_saturation);
   if( theta > 1.0 )
   { theta = 1.0; }

   // increase the speed of motiltiy up to a max of 1.5 micron/min
   phenotype.motility.is_motile = true;
   phenotype.motility.migration_speed = 1.5;
   phenotype.motility.migration_speed *= theta;

   phenotype.mechanics.cell_cell_adhesion_strength = (1.0-theta);
   phenotype.mechanics.cell_cell_adhesion_strength *=
      pCell->parameters.pReference_live_phenotype->mechanics.cell_cell_adhesion_strength;
   return;
}
```

[Return to Table of Contents.]

17.1.5 Example: a custom velocity update function

We'll add an example soon. For now, use the default, along with the default motility functions (which allow considerable customization!).

17.1.6 Example: analytical basement membrane functions

This space held in reserve.

17.1.7 Example: a custom cell orientation function

This space held in reserve.

17.2 Cell cycle models

17.2.1 Creating a custom cell cycle model

Let's create a simple cycle model, with three phases: Red, Green, and Blue. Red cells become Green. Green cells become Blue. Blue cells divide into two Red cells. Here's how we accomplish this using the

Cycle_Model class. Notice that we're saving the indices of the newly-created phases for easier reference.

```
Cycle_Model model;

// set up name information
model.code = PhysiCell_constants::custom_cycle_model;
mode.name = "Red Green Blue";

// add the phases
int red_index = model.add_phase( 0 , "Red" );
int green_index = model.add_phase( 1 , "Green" );
int blue_index = model.add_phase( 2 , "Blue" );

// set the Blue phase to have division at its end
model.phases[blue_index].division_at_phase_exit = true;

// set up the phase links
model.add_phase_link( red_index , green_index , NULL ); // red -> green
model.add_phase_link( green_index , blue_index , NULL ); // green -> blue
model.add_phase_link( blue_index , red_index , NULL ); // blue -> red

// set the transition rates
model.transition_rate(red_index,green_index) = 1.0/( 60.0 * 5.0 );
     // mean duration: 5 hours
model.transition_rate(green_index,blue_index) = 1.0/( 60.0 * 8.0 );
     // mean duration: 8 hours
model.transition_rate(blue_index,red_index) = 1.0/( 60.0 * 2.0 );
     // mean duration: 2 hours

// display the model
model.display( std::cout );
```

And this is how we would register that model in a cell definition:

```
immune_cell.phenotype.cycle.sync_to_cycle_model( rgb_model );
```

Or in a single cell:

```
pCell->phenotype.cycle.sync_to_cycle_model( rgb_model );
```

[Return to Table of Contents.]

17.2.2 Adding an arrest function

Suppose now that we only want to allow Blue cells to proceed to Red and divide if they have a fluid fraction over 50%. (See Section 11.3.) Then we can define an arrest function:

```
bool fluid_arrest_function(  Cell* pCell, Phenotype& phenotype, double dt )
{
     if( phenotype.volume.fluid_fraction < 0.5 )
     { return true; }
     return false;
}
```

We then assign this arrest function to the transition from the Blue phase to the Red phase:

```
rgb_model.phase_link(blue_index,red_index).arrest_function = fluid_arrest_function;
```

[Return to Table of Contents.]

17.2.3 Adding a custom phase entry function

Suppose now that we want the cell to "mutate" its transition rates whenever it re-enters the Red phase. We can do this with an entry function:

```
void my_mutation_function( Cell* pCell, Phenotype& phenotype, double dt )
{
     // mutate all transition rates by a uniformly-distributed
     // number within 10\% of the current value
     double multiplier = 0.9 + 0.2*uniform_random();
     phenotype.cycle.data.transition_rate(0,1) *= multiplier;

     multiplier = 0.9 + 0.2*uniform_random();
     phenotype.cycle.data.transition_rate(1,2) *= multiplier;

     multiplier = 0.9 + 0.2*uniform_random();
     phenotype.cycle.data.transition_rate(2,0) *= multiplier;

     return;
}
```

Then, we assign this as the entry function for the Red phase:

```
rgb_model.phases[red_index].entry_function = my_mutation_function;
```

Alternatively, we can assign this as the exit function for the Blue-Red transition:

```
rgb_model.phase_link(blue_index,red_index).exit_function = my_mutation_function;
```

[Return to Table of Contents.]

18 Future

Several features are planned for upcoming PhysiCell releases:

1. We will further refine the MultiCellDS output functions to capture more of the cell's state, including custom variable values.
2. We will add functions to read in a saved simulation state.
3. We will add an XML-based configuration file.
4. We will add a function like `void contact_interaction_function(Cell*,Phenotype&,double)` to allow cell contact-based signaling and behavior changes.
5. We plan to start actively tracking the cell's list of mechanically interacting neighbors in `cell.state.neighbors`
6. We will further develop `cell.state.simple_pressure` and provide better examples.
7. We will create a standard `standard_update_orientation` function to let cells rotate towards a preferred orientation (which will be based upon its neighbors).
8. We will generalize the cell-cell adhesion model to allow differing levels of adhesion between different types of cells.
9. We will merge the `Vector_Variable` and `Variable` types for a more unified `Custom_Cell_Data` struture.
10. We may update `motilty` and associated update functions to build in some hysteresis (bias towards the last directoin of the day).
11. We will consider partial SBML support for importing molecular-scale models.

19 Some notes on parameter values

We chose reference proliferation and apoptosis rates for the generic breast epithelium line (calibrated to MCF-10A) so that the apoptotic fraction is approximately 2% [11], and the net proliferation rate (for the total cell population) is on the order of 0.04 hr^{-1} [1, 8, 13].

19.1 Live cycle model

For the Live cell model (Section 16.1.1), we fit the system of ODEs

$$\frac{d}{dt}[\texttt{Live}] = (b-d)[\texttt{Live}] \tag{33}$$

$$\frac{d}{dt}[\texttt{Apoptotic}] = d[\texttt{Live}] - \frac{1}{T_A}[\texttt{Apoptotic}], \tag{34}$$

and we note that

$$\begin{aligned}\frac{d}{dt}[\texttt{Total}] &= b[\texttt{Live}] - \frac{1}{T_A}[\texttt{Apoptotic}] \\ &= \left(b(1-\mathrm{AI}) + \frac{\mathrm{AI}}{T_A}\right)[\texttt{Total}] \\ &= r[\texttt{Total}] \end{aligned} \tag{35}$$

Here, $r = 0.04\ \mathrm{hr}^{-1}$, $\mathrm{AI} = 0.02$ is the apoptotic index, and $T_A = 8.6$ hour (Section 16.2.1). Thus,

$$b = \frac{r + \frac{1}{T_A}\mathrm{AI}}{1-\mathrm{AI}} \sim 0.0432\ \mathrm{hr}^{-1}. \tag{36}$$

To get the death rate d, we use a simple iterative fitting method (see `./documentation/matlab/tune_death_in_live_model`) to get $d \sim 0.00319\ \mathrm{hr}^{-1}$.

19.2 Ki67 Basic model

For the Ki67 Basic model (Section 16.1.2), we fit the system of ODEs

$$\frac{d}{dt}[\texttt{Ki67-}] = -\left(\frac{1}{T_Q} + d\right)[\texttt{Ki67-}] + \frac{2}{T_K}[\texttt{Ki67+}] \tag{37}$$

$$\frac{d}{dt}[\texttt{Ki67+}] = \frac{1}{T_Q}[\texttt{Ki67-}] - \left(\frac{1}{T_K} + d\right)[\texttt{Ki67+}] \tag{38}$$

$$\frac{d}{dt}[\texttt{Apoptotic}] = d\left([\texttt{Ki67-}] + [\texttt{Ki67+}]\right) - \frac{1}{T_A}[\texttt{Apoptotic}], \tag{39}$$

and we note that

$$\begin{aligned}\frac{d}{dt}[\texttt{Total}] &= \frac{1}{t_K}[\texttt{Ki67+}] - \frac{1}{T_A}[\texttt{Apoptotic}] \\ &= \left(\frac{\mathrm{KI}}{T_K} + \frac{\mathrm{AI}}{T_A}\right)[\texttt{Total}] \\ &= r[\texttt{Total}] \end{aligned} \tag{40}$$

We set $T_K = 13 + 2.5$ hr (the duration of the two phases in the Ki67 Advanced model), $r = 0.04\ \mathrm{hr}^{-1}$, and we set $\mathrm{AI} = 0.02$ as before and keep $d = 0.00319\ \mathrm{hr}^{-1}$ from the prior estimate. We thus need to fit T_Q. We use a simple iterative fitting method (see `./documentation/matlab/tune_Ki67_basic`) to get $T_Q \sim 4.59$ hr.

19.3 Ki67 Advanced model

For the Ki67 Advanced model (Section 16.1.3), we fit the system of ODEs

$$\frac{d}{dt}[\texttt{Ki67-}] = -\left(\frac{1}{T_Q}+d\right)[\texttt{Ki67-}] + \frac{1}{T_{K2}}[\texttt{Ki67+}]_2 \quad (41)$$

$$\frac{d}{dt}[\texttt{Ki67+}]_1 = \frac{1}{T_Q}[\texttt{Ki67-}] - \left(\frac{1}{T_{K1}}+d\right)[\texttt{Ki67+}]_1 \quad (42)$$

$$\frac{d}{dt}[\texttt{Ki67+}]_2 = \frac{2}{T_{K1}}[\texttt{Ki67+}]_1 - \left(\frac{1}{T_{K2}}+d\right)[\texttt{Ki67+}]_2 \quad (43)$$

$$\frac{d}{dt}[\texttt{Apoptotic}] = d\left([\texttt{Ki67-}]+[\texttt{Ki67+}]_1+[\texttt{Ki67+}]_2\right) - \frac{1}{T_A}[\texttt{Apoptotic}], \quad (44)$$

and we note that

$$\begin{aligned}\frac{d}{dt}[\texttt{Total}] &= \frac{1}{T_{K1}}[\texttt{Ki67+}]_1 - \frac{1}{T_A}[\texttt{Apoptotic}] \\ &= \left(\frac{\mathrm{KI}_1}{T_{K1}}+\frac{\mathrm{AI}}{T_A}\right)[\texttt{Total}] \\ &= r[\texttt{Total}] \quad (45)\end{aligned}$$

We set $T_{K1} = 13$ hr, $T_{K2} = 2.5$ hr, $T_A = 8.6$ hr, and $r = 0.04$ hr^{-1}, and we set AI $= 0.02$ as before and keep $d = 0.00319$ hr^{-1} from the prior estimate. We thus need to fit T_Q. We use a simple iterative fitting method (see `./documentation/matlab/tune_Ki67_advanced`) to get $T_Q \sim 3.62$ hr for this model. [12].

20 Acknowledgements

We thank the Breast Cancer Research Foundation, the Jayne Koskinas Ted Giovanis Foundation for Health and Policy, and the National Cancer Institute for past and present funding for PhysiCell. We gratefully acknowledge the encouragement and suport of the multiscale modeling community as we developed and refined MultiCellDS. We hope the community finds this software useful!

Paul Macklin thanks the Chaste, CompuCell3D, COPASI, and Morpheus communities and developers for Open Source leadership and inspiration.

[Return to Table of Contents.]

Bibliography

[1] S. H. Friedman, A. R. Anderson, D. M. Bortz, A. G. Fletcher, H. B. Frieboes, A. Ghaffarizadeh, D. R. Grimes, A. Hawkins-Daarud, S. Hoehme, E. F. Juarez, C. Kesselman, R. M. Merks, S. M. Mumenthaler, P. K. Newton, K.-A. Norton, R. Rawat, R. C. Rockne, D. Ruderman, J. Scott, S. S. Sindi, J. L. Sparks, K. Swanson, D. B. Agus, and P. Macklin. MultiCellDS: a standard and a community for sharing multicellular data. *PLoS Biol.*, 2017. doi: 10.1101/090696. URL http://dx.doi.org/10.1101/090696/. in revision.

[2] A. Ghaffarizadeh, S. H. Friedman, and P. Macklin. BioFVM: an efficient, parallelized diffusive transport solver for 3-D biological simulations. *Bioinformatics*, 32(8):1256–8, 2016. doi: 10.1093/bioinformatics/btv730. URL http://dx.doi.org/10.1093/bioinformatics/btv730.

[3] A. Ghaffarizadeh, S. H. Friedman, S. M. Mumenthaler, and P. Macklin. PhysiCell: an open source physics-based cell simulator for 3-D multicellular systems. *PLoS Comput. Biol.*, 2017. doi: 10.1101/088773. URL http://dx.doi.org/10.1101/088773. (in revision).

[4] A. Z. Hyun and P. Macklin. Improved patient-specific calibration for agent-based cancer modeling. *J. Theor. Biol.*, 317:422–4, 2013. doi: 10.1016/j.jtbi.2012.10.017. URL http://dx.doi.org/10.1016/j.jtbi.2012.10.017.

[5] B. Jähne. *Practical Handbook on Image Processing for Scientific Applications.* CRC Press, Boca Raton, FL USA, 1997. ISBN 978-0849389061.

[6] A. Kapoulkine. pugixml: Light-weight, simple and fast XML parser for C++ with XPath support, 2016. URL https://github.com/zeux/pugixml.

[7] P. Macklin, M. E. Edgerton, A. M. Thompson, and V. Cristini. Patient-calibrated agent-based modelling of ductal carcinoma in situ (DCIS): From microscopic measurements to macroscopic predictions of clinical progression. *J. Theor. Biol.*, 301:122–40, 2012. doi: 10.1016/j.jtbi.2012.02.002. URL http://dx.doi.org/10.1016/j.jtbi.2012.02.002.

[8] NCI PS-OC Bioresource Core Facility. Thawing, propagating, and cryopreserving protocol: MCF10A-JSB breast epithelium (NCI-PBCF-1000), 2012. URL http://physics.cancer.gov/docs/bioresource/breast/NCI-PBCF-1000-SOP-052814_508.pdf.

[9] Persistence of Vision Pty. Ltd. Persistence of vision raytracer, 2004. URL http://www.povray.org.

[10] A. C. Ruifrok and D. A. Johnston. Quantification of histochemical staining by color deconvolution. *Anal. Quant. Cytol. Histol.*, 23(4):291–9, 2001. URL https://www.ncbi.nlm.nih.gov/pubmed/11531144.

[11] A. Sadlonova, Z. Novak, M. R. Johnson, D. B. Bowe, S. R. Gault, G. P. Page, J. V. Thottassery, D. R. Welch, and A. R. Frost. Breast fibroblasts modulate epithelial cell proliferation in three-dimensional in vitro co-culture. *Br. Canc. Res.*, 7(1):R46, Nov 2004. ISSN 1465-542X. doi: 10.1186/bcr949. URL https://doi.org/10.1186/bcr949.

[12] M. Vaapil, K. Helczynska, R. Villadsen, O. W. Petersen, E. Johansson, S. Beckman, C. Larsson, S. Phlman, and A. Jgi. Hypoxic conditions induce a cancer-like phenotype in human breast epithelial cells. *PLoS One*, 7(9):1–12, 09 2012. doi: 10.1371/journal.pone.0046543. URL https://doi.org/10.1371/journal.pone.0046543.

[13] N. D. Zantek, J. Walker-Daniels, J. Stewart, R. K. Hansen, D. Robinson, H. Miao, B. Wang, H.-J. Kung, M. J. Bissell, and M. S. Kinch. MCF-10A-NeoST: A new cell system for studying cell-ecm and cell-cell interactions in breast cancer. *Clinical Cancer Research*, 7(11):3640–3648, 2001. ISSN 1078-0432. URL http://clincancerres.aacrjournals.org/content/7/11/3640.
