# Supplementary material for: PhysiCell: An open source physics-based cell simulator for 3-D multicellular systems
Source: PLoS Comput Biol. 2018 Feb 23;14(2):e1005991. doi: 10.1371/journal.pcbi.1005991 (PMC5841829; doi:10.1371/journal.pcbi.1005991)
Supplement: S3 Text — A fast guide to building, running, and visualizing your first PhysiCell simulations. We recommend that new users start with this guide. (PDF) [file pcbi.1005991.s003.pdf]

MathCancer / **PhysiCell**

Branch: master PhysiCell / documentation / Quickstart.md Find file Copy path

rheiland Update Quickstart.md bf2d776 26 minutes ago

1 contributor

178 lines (131 sloc) 8.23 KB

Quickstart Guide to PhysiCell

Download

We currently provide 2 options for downloading PhysiCell:

1. Downloaded source code as a .zip file from http://physicell.mathcancer.org/ (the link there takes you to the latest release on sourceforge).
2. We have recently begun migrating our code to GitHub. So, you can also download from https://github.com/MathCancer/PhysiCell/releases.

For more detailed information, see Section 3 of the User Guide (in `/documentation`) and/or also http://www.mathcancer.org/blog/physicell-tutorials/.

Build: the basics

PhysiCell is written in C++ and should build on any of the three major operating systems. The one **requirement is that your compiler support OpenMP**. If, during your build (make) process, you get a message like: `error: unsupported option '-fopenmp'`, you'll know your Makefile is trying to use a compiler that doesn't support OpenMP. You may need to install an OpenMP-supported compiler and/or edit the Makefile to use it.

Windows

The currently preferred way to use PhysiCell on Windows is to install MinGW-w64 which will let you use a version of GCC that supports OpenMP. For more detailed information, see our blog post.

(Sometime in the near future, we plan to provide an option to use native Windows and a recent Microsoft Visual C++ compiler, using CMake to build PhysiCell. If you want to be a beta-tester, contact us).

OSX

Unfortunately, the C++ compiler provided by the latest version of XCode on OSX does not support OpenMP. To resolve this, we recommend using the `brew` package manager to install a recent version of `gcc`. Follow the brew installation instructions.

After installing brew, type `brew install gcc` from a Terminal command line. This should install a recent version of gcc/g++ (supporting OpenMP) into `/usr/local/bin`. You can verify this with:

```
$ ls -l /usr/local/bin/g++*
lrwxr-xr-x  1 heiland  admin  29 Oct  3 14:38 /usr/local/bin/g++-7@ -> ../Cellar/gcc/7.2.0/bin/g++-7
```

Set the following environment variable in your Terminal's shell, e.g., in the bash shell:

```
$ export PHYSICELL_CPP=/usr/local/bin/g++-7
```

and the Makefile will use it. You should permanently set this in your environment via:

`$ echo export PHYSICELL_CPP=g++-7 >> ~/.bash_profile`.

Linux

If you're a Linux user, you probably already have or know how to install/use a proper g++ environment for building PhysiCell. If not, contact us!

Build: sample projects

In the current release of PhysiCell, we provide four sample projects to help you get started. Three of the projects are 2D models (*biorobots, anti-cancer biorobots, and cancer heterogeneity*); the fourth project is a 3D model (*cancer immunology*). The procedure to build and execute each of the sample projects follows the same pattern. For example, from your Terminal, in the root PhysiCell directory/folder:

```
$ make biorobots-sample     # populate the biorobots sample project
$ make                      # compile the project
```

Assuming the project builds without errors, you should now have an executable called `biorobots` which you can run, e.g.:

```
$ ./biorobots
```

This will begin the simulation, write information to your terminal, and generate output files of types `.svg`, `.xml`, and `.mat`. More about those below. You can `Control-c` to kill the simulation early, if you want.

For the remaining three example projects provided with PhysiCell, you would follow similar steps, but first, you may want to clean out the previous simulation's output and prepare for the new one:

```
$ make data-cleanup   # Delete output data. (Optionally, manually move it to another directory to keep it)
$ make reset          # clear out the sample project / clean slate
$ make cancer-biorobots-sample    # populate the new project
$ make                            # compile the project
$ ./cancer_biorobots
```

and

```
$ make data-cleanup
$ make reset
$ make heterogeneity-sample
$ make
$ ./heterogeneity
```

and

```
$ make data-cleanup
$ make reset
$ make cancer-immune-sample
$ make
$ ./cancer_immune_3D
```

Visualizing Output

PhysiCell does not currently provide a GUI for visualizing output results. Our approach, at least for now, is to suggest and offer guidance on using other tools, e.g. your browser, ImageMagick, MATLAB, Octave, ParaView, and more.

Browser

At a bare minimum, you should be able to use your browser to `File -> Open` any .svg (scalable vector graphics) file that your simulation generates. PhysiCell simulates transmitted light microscopy to create virtual pathology images for the .svg files. Even for 3D models, 2D cross-section images (.svg files) are generated, by default, using a slice through the Z=0 plane, as depicted in the following image (from the cancer-immune-sample project).

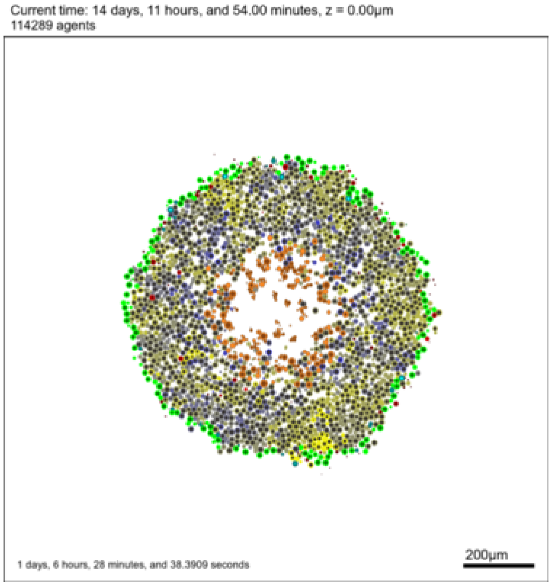

MATLAB/Octave

If you have access to MATLAB (or Octave), we already have a detailed tutorial on how to visualize the MultiCellDS digital snapshots (.xml and .mat files) at http://www.mathcancer.org/blog/working-with-physicell-snapshots-in-matlab/.

ImageMagick

If you are able to install ImageMagick (with SVG support) on your computer, you will have access to several image processing command line tools that will let you filter and arrange images. For example, the following commands:

```
$ montage -geometry +0+0 -tile 4x1 snapshot00000100.svg snapshot00000200.svg snapshot00000300.svg snapshot00000400.svg tmp.png
$ convert -resize 15% tmp.png out.png
```

will generate a tiled horizontal sequence of images:

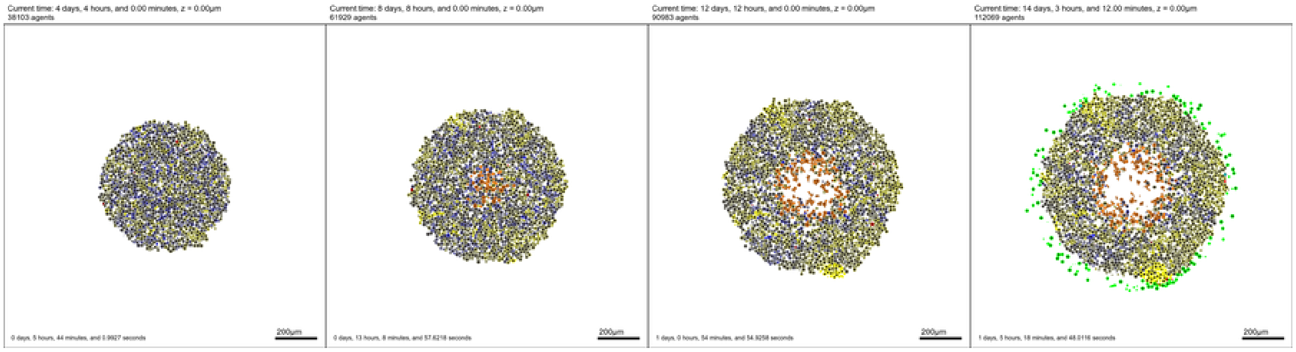

ImageMagick will also let you generate an animated gif of your results, e.g.:

```
$ convert snapshot000034*.svg foo.gif
$ magick animate foo.gif    # may be huge, if original SVGs were; downsize in following steps
$ convert foo.gif -coalesce tmp.gif
$ identify snapshot00003471.svg    # get size of a single image (e.g. 1500x1605)
$ convert -size 1500x1605 tmp.gif -resize 20% small.gif
$ magick animate small.gif
```

ParaView

If you install ParaView, you can visualize and interact with output from 3D models (the `.mat` files). We will be providing a detailed tutorial for this. Until then, please ask questions.

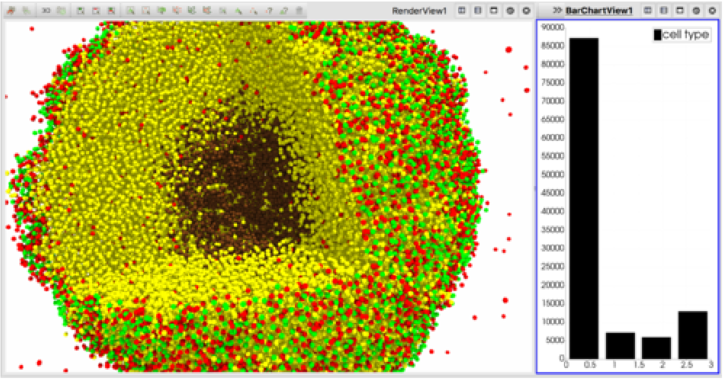

Support

Until we set up a mailing list, please email us directly (macklinp@iu.edu, heiland@iu.edu) and follow us on Twitter (https://twitter.com/MathCancer).

References

- http://physicell.mathcancer.org/
- http://www.mathcancer.org/blog/setting-up-a-64-bit-gcc-environment-on-windows
- http://www.mathcancer.org/blog/setting-up-gcc-openmp-on-osx-homebrew-edition/
- http://www.mathcancer.org/blog/physicell-tutorials/
- http://www.mathcancer.org/blog/working-with-physicell-snapshots-in-matlab/
- https://github.com/MathCancer/PhysiCell/blob/master/documentation/User_Guide.pdf
